# Supplementary material for: A scoping review on biomedical journal peer review guides for reviewers
Source: PLoS One. 2021 May 20;16(5):e0251440. doi: 10.1371/journal.pone.0251440 (PMC8136639; doi:10.1371/journal.pone.0251440)
Supplement: S2 File — (DOCX) [file pone.0251440.s002.docx]

**S2 File. Search strategy**

Databases: PubMed, Embase, AMED

Search date: Until 19 February 2021

**PubMed**

(reviewing[Text Word] OR reviewer[Text Word] OR peer review[Text Word] OR peer reviewer[Text Word] OR peer-review[Text Word] OR peer-reviewer[Text Word] OR referee*[Text Word] OR peer review[MeSH terms]) AND (journal[Text Word] OR journals[Text Word] OR manuscript[Text Word] OR manuscripts[Text Word]) AND (guide[Text Word] OR guideline[Text Word] OR guidelines[Text Word] OR how to[Text Word] OR lesson[Text Word] OR tips[Text Word] OR advice*[Text Word] OR recommendation*[Text Word] OR report form[Text Word] OR checklist[Text Word])

**Embase**

ab(reviewing OR reviewer OR peer review OR peer reviewer OR peer-review OR peer-reviewer OR referee*) AND ab(journal OR journals OR manuscript OR manuscripts) AND ab(guide OR guideline OR guidelines OR how to OR lesson OR tips OR advice* OR recommendation* OR report form OR checklist)

**AMED**

(reviewing OR reviewer OR peer review OR peer reviewer OR peer-review OR peer-reviewer OR referee*) AND (journal OR journals OR manuscript OR manuscripts) AND (guide OR guideline OR guidelines OR how to OR lesson OR tips OR advice* OR recommendation* OR report form OR checklist)
