# Supplementary material for: A scoping review on biomedical journal peer review guides for reviewers
Source: PLoS One. 2021 May 20;16(5):e0251440. doi: 10.1371/journal.pone.0251440 (PMC8136639; doi:10.1371/journal.pone.0251440)
Supplement: S3 File — (DOCX) [file pone.0251440.s003.docx]

**S3 File. Included review checklist items.**

**S3A.** List of checklist items from included articles (1,077 items)

| Title | | |
| --- | --- | --- |
| 1 | Accurate/adequate title | Alexander (2005) |
| 2 | Does the title clearly indicate the study design, i.e. observational study, randomized trial, meta-analysis, other? | Allen (2014) |
| 3 | Is the title specific and does it reflect the content of the manuscript? | Benos et al. (2003) |
| 4 | Is the qualitative focus identified (e.g., phenomenology, ethnographic, grounded theory, historical, case study) in the title or introduction, and does the manuscript reflect an appropriate example of the identified focus? | Christenbery (2011) |
| 5 | Is the title appropriate? | Heddle and Ness (2009) |
| 6 | Title is appropriate. | Lippi (2018) |
| 7 | Does the title accurately reflect the content? | Marušic et al. (2005) |
| 8 | Does the title specify the type and the setting of the study? | Marušic et al. (2005) |
| 9 | Does the title reflect the contents? | Paice (2001) |
| 10 | Adequate relevance of title to research (Is all relevant information contained in the title so the reader gets the right impression of the article from the title?) | Schuttpelz-Brauns et al. (2010) |
| 11 | Does the title accurately reflect the purpose, design, results, and conclusions of the study? | Seals and Tanaka (2000) |
| 12 | Title is concise, understandable and representative of manuscript’s content. | Simpson (2008) |
| 13 | Does the title accurately describe the manuscript? | Smith et al. (2018) |
| 14 | Is the title worded in a way that would catch a reader’s attention? | Smith et al. (2018) |
| 15 | Is the title informative? Is it too long? | Smolčić and Simundić (2014) |
| 16 | Does the title relate to the content of the article? | Smolčić and Simundić (2014) |
| 17 | Does it “stand alone” as an accurate representation of the study? | Sucato and Holland-Hall (2018) |
| 18 | Is the title appropriately descriptive of the study? | Sucato and Holland-Hall (2018) |
| 19 | Is the title specific and does it reflect the content of the manuscript? | Tandon (2014) |
| 20 | The referee should provide recommendations on how to improve the title and abstract, as appropriate – many working titles are too generic and not reflective of the study design and manuscript content | Stahel and Moore (2016) |
| 21 | Whether appropriate, clear, concise and precise. | Tullu and Karande (2020) |
| 22 | All essential components (population, intervention, control, and outcome i.e., PICO) are mentioned (may vary with the type of the study).Related to the topic/content of the paper and does not (usually) reveal the result. | Tullu and Karande (2020) |
| 23 | Is the title appropriate? Does it accurately represent the study? | Pai (2020) |
| Abstract | | |
| 24 | Is the abstract concise? | Marušic et al. (2005) |
| 25 | Does the abstract specify outcome measures? | Marušic et al. (2005) |
| 26 | Are numerical data presented in the abstract? | Marušic et al. (2005) |
| 27 | Does the conclusion relate directly to the results of the study in the abstract? | Marušic et al. (2005) |
| 28 | Summary addresses study purpose. | Alexander (2005) |
| 29 | Accurate/adequate abstract. | Alexander (2005) |
| 30 | Does the abstract correctly reflects the full content of the manuscript? | Alexandrov et al. (2009) |
| 31 | Will the abstract garner the reader’s attention and encourage the reader to read on? | Allen (2014) |
| 32 | Does the abstract include all the necessary components, e.g. hypothesis/objective, methods, results, conclusion? | Allen (2014) |
| 33 | Are the main points of the paper adequately summarized? | Allen (2014) |
| 34 | Is the abstract an optimal summary? The conclusion of an abstract is probably the most important sentence in a manuscript—make sure it is a clear and reasonable reflection of the work. | Allen and Ho (2017) |
| 35 | Is the abstract brief and does it indicate the purpose of the work, what was done, what was found, and the significance? | Benos et al. (2003) |
| 36 | Does the abstract accurately reflect the manuscript? | Heddle and Ness (2009) |
| 37 | Does the abstract stand alone, able to be understood without reading the full manuscript? | Hill (2016) |
| 38 | Is the abstract focused on data and conclusions? | Lippi (2018) |
| 39 | Is the abstract structured? | Marušic et al. (2005) |
| 40 | Is the abstract a fair summary, properly structured? | Paice (2001) |
| 41 | Does the Abstract appropriately summarize the manuscript? | Provenzale and Stanley (2005) |
| 42 | Are there discrepancies between the Abstract and the remainder of the manuscript? | Provenzale and Stanley (2005) |
| 43 | Can the Abstract be understood without reading the manuscript? | Provenzale and Stanley (2005) |
| 44 | Are objectives stated clearly in the abstract? | Rosenfeld (2010) |
| 45 | Is the sample size stated clearly in the abstract and text? | Rosenfeld (2010) |
| 46 | Abstract: How participants were allocated to interventions | Rostami et al. (2011) |
| 47 | Summary - short overview of results (Is all relevant information contained in the summary so the reader gets an accurate overview over the article?) | Schuttpelz-Brauns et al. (2010) |
| 48 | Abstract/Summary: Is this a succinct, clear, and comprehensive summary of the main text of the paper? | Seals and Tanaka (2000) |
| 49 | Abstract/Summary: Is the content (data, conclusions, etc.) consistent with that presented in the main text? | Seals and Tanaka (2000) |
| 50 | Abstract/Summary: Are data or other key information presented here but not in the main text (or vice versa)? | Seals and Tanaka (2000) |
| 51 | Abstract: Is concise, approximately within word limit. Enough detail for the reader to understand purpose of study, what was measured and how measures were obtained. Enough results to know data outcomes. Some interpretation of the data exist so that the “what does it mean and why is it important” is evident. | Simpson (2008) |
| 52 | Abstract: Is the abstract succinct? | Smith et al. (2018) |
| 53 | Abstract: Does the abstract accurately convey the purpose, research aim, results, and conclusion of the manuscript? | Smith et al. (2018) |
| 54 | Abstract: Is the *Abstract* structured? | Smolčić and Simundić (2014) |
| 55 | Abstract: Do the authors list the number of patients/groups and study design? | Smolčić and Simundić (2014) |
| 56 | Abstract: Do the authors provide their key results (with numbers and P values)? | Smolčić and Simundić (2014) |
| 57 | Abstract: Is the conclusion based on the results of the study? | Smolčić and Simundić (2014) |
| 58 | Abstract: Is the conclusion in the *Abstract* identical to the conclusion at the end of the *Discussion*? | Smolčić and Simundić (2014) |
| 59 | Abstract: Is there an aim and a hypothesis? | Smolčić and Simundić (2014) |
| 60 | Is any critical information missing from the abstract? | Sucato and Holland-Hall (2018) |
| 61 | Abstract should provide a quick summary of the case and that a comprehensive literature review and discussion about the entity will be provided. | Talanow (2014) |
| 62 | Abstract: (a) Does it meet the word limits of the Journal? | Tandon (2014) |
| 63 | Abstract: (b) Does it appropriately summarize the manuscript? | Tandon (2014) |
| 64 | Abstract: (c) Are there discrepancies between the abstract and the remainder of the manuscript? | Tandon (2014) |
| 65 | Abstract: (d) Can the abstract be understood without reading the remainder of the manuscript? | Tandon (2014) |
| 66 | Abstract: (e) Whether structured or unstructured (should meet Journal guidelines), does it have information about the following 4 elements | Tandon (2014) |
| 67 | Does the exposition of the paper help to clarify our understanding of this area of research or application? Does the paper hold our interest and make us want to give the paper the careful reading that we give to important papers in our area of specialization? | Wilson (2002) |
| 68 | Concise and follows guidelines to authors stated by the journal. | Tullu and Karande (2020) |
| 69 | Usually structured- Background/introduction, aims, methods, results, conclusions (or similar subsections). | Tullu and Karande (2020) |
| 70 | Whether it is independent, complete and stand-alone (i.e., give a fair idea of the contents of the paper without reading the complete manuscript). | Tullu and Karande (2020) |
| 71 | Reflects the content of the article accurately (especially after a revision). | Tullu and Karande (2020) |
| 72 | Is it consistent with the content of the main text? | Tullu and Karande (2020) |
| 73 | Word limit stated by the journal is adhered to (usually about 250 words). | Tullu and Karande (2020) |
| 74 | Keywords from MeSH terms (Pubmed website) are correctly mentioned at the end of the abstract | Tullu and Karande (2020) |
| 75 | Do the abstract faithfully represent the study? | Pai (2020) |
| Keywords | | |
| 76 | Keywords: Useful search terms. Keywords should not replicate any words in the title. | Simpson (2008) |
| 77 | Key words: Are the keywords appropriate? Do they reflect the content of the article? | Smolčić and Simundić (2014) |
| 78 | Appropriate keywords | Duchesne (2008) |
| 79 | Keywords should include the diagnosis and also its eponyms and alternative names | Talanow (2014) |
| 80 | Are the keywords appropriate? | Pai (2020) |
| Introduction | | |
| 81 | Introduction: - Does the introduction clearly state the purpose and hypothesis of the manuscript? | Allen (2014) |
| 82 | Introduction: - Does the author(s) provide adequate context for the topic, e.g. why is the topic important and timely? | Allen (2014) |
| 83 | Introduction: Clinical or methodological context | Duchesne (2008) |
| 84 | Introduction: Positioning with respect to literature | Duchesne (2008) |
| 85 | Introduction: Study objectives | Duchesne (2008) |
| 86 | Introduction: Study hypotheses | Duchesne (2008) |
| 87 | Does the introduction inform the hypothesis? | Heddle and Ness (2009) |
| 88 | Does the Introduction lay out the rationale for the study and explain its goals? The Introduction should not provide a lengthy and detailed review of the field. | Hill (2017) |
| 89 | Was a thorough review of pre-existing research conducted? | Kotsis and Chung (2014) |
| 90 | Were other research studies properly referenced? | Kotsis and Chung (2015) |
| 91 | Is the rationale for the question provided? | Kotsis and Chung (2016) |
| 92 | Are the purpose and/or hypothesis clearly stated? | Kotsis and Chung (2017) |
| 93 | Clarity of study purpose/goals/hypothesis | Alexander (2005) |
| 94 | Introduction: 1. Is the rationale (based on cited critical observations, rigorous data, or accepted opinion) for the questions or purposes provided? Is the logic of that rationale clear? | Brand (2012) |
| 95 | Introduction: 2. Do the authors pose clear questions, hypotheses, or purposes (goals, objectives, aims)? Clear questions and unambiguous hypotheses most efficiently advance science. | Brand (2012) |
| 96 | Introduction: 3. Once posed, is the question novel? Do the authors document novelty by proper citation? If the question is not novel, have others reported similar work, whether or not cited? If the work essentially repeats that of others, does it confirm previously unconfirmed or inadequately confirmed observations or does it address explicit controversies? | Brand (2012) |
| 97 | Introduction: 4. Are the questions important? Do the authors explore and explicitly state the implications of the answers? | Brand (2012) |
| 98 | Introduction: 5. Are the questions or hypotheses described in terms of independent and dependent variables? | Brand (2012) |
| 99 | Theoretical foundations: Is there complete articulation of a theory or conceptual framework? | Christenbery (2011) |
| 100 | Theoretical foundations: Is there congruence between the theoretical or conceptual framework and the problem? | Christenbery (2011) |
| 101 | Purpose: Has the author identified the phenomenon of interest and stated why a qualitative approach was use? | Christenbery (2011) |
| 102 | Is the study aimed at solving a practical clinical issue? | England and Cheng (2019) |
| 103 | Is the purpose of the study clearly stated? | England and Cheng (2019) |
| 104 | Is the hypothesis and research question clearly defined? | Heddle and Ness (2009) |
| 105 | Provide exhaustive comments: Introduction centred on topic and aims of the study | Lippi (2018) |
| 106 | Introduction: does it justify performing the study? | Marušic et al. (2005) |
| 107 | Introduction: does it end with the hypothesis? | Marušic et al. (2005) |
| 108 | Introduction: does the hypothesis arise logically from the theoretical framework? | Marušic et al. (2005) |
| 109 | Did the authors give a clear idea of the target readership and why the research was carried out? | Oerther and Watson (2019) |
| 110 | Was the problem easy to identify? | Oerther and Watson (2019) |
| 111 | Was the literature review up‐to‐date and based mainly on primary sources? | Oerther and Watson (2019) |
| 112 | Did the authors provide state‐of‐the‐art synthesis of the literature? Alternatively, did the authors describe a few previous studies and forgo synthesis? | Oerther and Watson (2019) |
| 113 | Did the authors provide a strong rationale for the study? | Oerther and Watson (2019) |
| 114 | Did the authors highlight gaps in current understanding or conflicts in current knowledge? | Oerther and Watson (2019) |
| 115 | Did the authors define key concepts adequately? | Oerther and Watson (2019) |
| 116 | Was a conceptual/theoretical framework articulated? If not, is the absence justified? | Oerther and Watson (2019) |
| 117 | Introduction: Does this set the work in context? | Paice (2001) |
| 118 | Is the purpose of the study clearly defined? | Provenzale and Stanley (2005) |
| 119 | Do the authors provide a rationale for performing the study based on a review of the medical literature? If | Provenzale and Stanley (2005) |
| 120 | Is the introduction of the appropriate length? | Provenzale and Stanley (2005) |
| 121 | Do the authors define terms used in the remainder of the manuscript? | Provenzale and Stanley (2005) |
| 122 | If this manuscript is Original Research, is there a well-defined hypothesis? | Provenzale and Stanley (2005) |
| 123 | Is the Introduction concise? | Provenzale and Stanley (2005) |
| 124 | Are the objectives and hypotheses appropriate? | Rosenfeld (2010) |
| 125 | Objectives and hypothesis: Specific, measurable, achievable, realistic and time framed (SMART) | Rostami et al. (2011) |
| 126 | Scientific background and explanation of rationale | Rostami et al. (2011) |
| 127 | Introduction - Background, definition of problem, question hypothesis and aims (does the intro present a clear conceptual framework? Is the current state of research adequately backed up by the literature? Are knowledge gaps identified which the present work aims to deal with? Is a clear research question/hypothesis formulated or the project aim set out?) | Schuttpelz-Brauns et al. (2010) |
| 128 | Introduction: Does the introduction succinctly state what is known and unknown about the topic? | Seals and Tanaka (2000) |
| 129 | Introduction: Are any important findings from previous studies omitted or misrepresented? | Seals and Tanaka (2000) |
| 130 | Introduction: Is the specific experimental question, goal, or aim to be addressed stated? | Seals and Tanaka (2000) |
| 131 | Introduction: Are previous experimental observations linked together to establish a formally stated and testable working hypothesis? Does the hypothesis clearly indicate the direction of the postulated effect? | Seals and Tanaka (2000) |
| 132 | Introduction: If previous reports have addressed the same topic: *1*) are their strengths and limitations described such that the need for further study is established? and *2*) is it clear how the experimental approach to be used in the present study is likely to yield more definitive or unique insight than these previous studies? | Seals and Tanaka (2000) |
| 133 | Is the problem investigated important and original? Is it is just a rehash of previous work in the area? | Simpson (2008) |
| 134 | Purpose(s) or aim(s) clearly stated and fit well with the problem being investigated. | Simpson (2008) |
| 135 | What does the author surmise will happen? Is there explanation/justification for the expected outcomes that are based on scientific principles and/or scientifically logical? | Simpson (2008) |
| 136 | Are relevant trials included in the author’s assessment of the background literature? | Smith et al. (2018) |
| 137 | Are there any recently published studies that should be included in the author’s background, but are not? | Smith et al. (2018) |
| 138 | Is the research question clearly stated? | Smith et al. (2018) |
| 139 | Do the authors list specific reasons for completing the study? | Smith et al. (2018) |
| 140 | Is there a clear and unambiguous aim at the end of the *Introduction*? | Smolčić and Simundić (2014) |
| 141 | Do the authors explain the background of the problem? | Smolčić and Simundić (2014) |
| 142 | Do the authors clearly elaborate their hypothesis? | Smolčić and Simundić (2014) |
| 143 | Introduction/hypothesis - Why was the study performed?: Do the authors define a relevant knowledge gap? Have they given appropriate credit to previous work in the field? Is the hypothesis clinically relevant and of scientific merit? Does the study address an important unresolved problem in the field? Will it contribute to improvement in the quality of the clinical care delivered to patients or help resolve a previously unknown basic experimental question? | Stahel and Moore (2016) |
| 144 | Coherence and comprehensiveness of the background section; this section should end with the specific hypothesis or stated goal of the study | Stahel and Moore (2016) |
| 145 | Does the introduction easily lead the reader to the research question and hypothesis? | Stone et al. (2018) |
| 146 | Do the authors clearly state the purpose of the study and/or a hypothesis? | Sucato and Holland-Hall (2018) |
| 147 | Is a contextual framework provided, explaining why the topic is worth studying? | Sucato and Holland-Hall (2018) |
| 148 | Is it clear how this study would add to the field? | Sucato and Holland-Hall (2018) |
| 149 | Does the hypothesis address a trivial issue or one that has the potential to be clinically relevant? Has this hypothesis been investigated before? If so, has the author justified the reexamination? | Sylvia and Herbel (2001) |
| 150 | Is scientifically up-to-date information provided? | Talanow (2014) |
| 151 | Introduction: (a) Is it concise? | Tandon (2014) |
| 152 | Introduction: (b) Is the purpose of the study clearly laid out? | Tandon (2014) |
| 153 | Introduction: (c) Is a rationale for the study provided on the basis of a succinct review of the literature (‘‘what gap in the existing literature does this study seeking to address’’)? | Tandon (2014) |
| 154 | Introduction: (d) Are ‘‘unusual’’ or idiosyncratic terms defined? | Tandon (2014) |
| 155 | Introduction: (e) What is the specific hypothesis being tested? | Tandon (2014) |
| 156 | Aim: a clearly formulated research question | Hunter (2020) |
| 157 | Gives the rationale in brief. | Tullu and Karande (2020) |
| 158 | Gives adequate background information and context. | Tullu and Karande (2020) |
| 159 | Gives only the relevant literature and few (four to eight) appropriate references. | Tullu and Karande (2020) |
| 160 | Is the research question important? | Tullu and Karande (2020) |
| 161 | Whether the objectives and purpose of the study are clearly stated. | Tullu and Karande (2020) |
| 162 | Is there any major flaw in the hypothesis or aim? | Pai (2020) |
| 163 | Does the introduction make it clear as to why the authors set out to perform the study? | Pai (2020) |
| 164 | Is the introduction too long? | Pai (2020) |
| 165 | Is the rationale for including a HRQOL assessment reasonable? | Lapin (2020) |
| 166 | Does the chosen HRQOL measure/domain align with the study objective(s)? | Lapin (2020) |
| 167 | Briefly addresses: scope of the problem - gap in knowledge | Brown et al (2017) |
| 168 | Briefly addresses:prior research - flaws in prior research | Brown et al (2017) |
| 169 | How does this study fill the knowledge gap? | Brown et al (2017) |
| 170 | Primary aim of the study / hypothesis | Brown et al (2017) |
| Methods | | |
| 171 | Target population: Has the target patient population for decision been clearly defined? | Ades et al. (2013) |
| 172 | Comparators: Have all the appropriate treatments in the decision been identified? | Ades et al. (2013) |
| 173 | Comparators: Are there additional treatments in the synthesis comparator set that are not in the decision comparator set? If so, is this adequately justified? | Ades et al. (2013) |
| 174 | Trial inclusion/exclusion: Is the search strategy technically adequate and appropriately reported? | Ades et al. (2013) |
| 175 | Trial inclusion/exclusion: Have all trials involving at least 2 of the treatments in the synthesis comparator set been included? | Ades et al. (2013) |
| 176 | Trial inclusion/exclusion: Have all trials reporting relevant outcomes been included? | Ades et al. (2013) |
| 177 | Trial inclusion/exclusion: Have additional trials been included? If so, is this adequately justified? | Ades et al. (2013) |
| 178 | Treatment definition: Are all the treatment options restricted to specific doses and co-treatments, or have different doses and co-treatments been ‘‘lumped’’ together? If the latter, is it adequately justified? | Ades et al. (2013) |
| 179 | Treatment definition: Are there any additional modeling assumptions? | Ades et al. (2013) |
| 180 | Trial outcomes and scale of measurement chosen for the synthesis: Where alternative outcomes are available, has the choice of outcome measure used in the synthesis been justified? | Ades et al. (2013) |
| 181 | Have the assumptions behind the choice of scale been justified? | Ades et al. (2013) |
| 182 | Do some trials include patients outside the target population? If so, is this adequately justified? | Ades et al. (2013) |
| 183 | What assumptions are made about the impact or lack of impact this may have on the relative treatment effects? Are they adequately justified? | Ades et al. (2013) |
| 184 | Has an adjustment been made to account for these differences? If so, comment on the adequacy of the evidence presented in support of this adjustment and on the need for a sensitivity analysis. | Ades et al. (2013) |
| 185 | Patient Population Heterogeneity: Have potential modifiers of treatment effect been considered? | Ades et al. (2013) |
| 186 | Patient Population Heterogeneity: Are there apparent or potential differences between trials in their patient populations, albeit within the target population? If so, has this been adequately taken into account? | Ades et al. (2013) |
| 187 | Meta-Analytic Methods: Has the software implementation been documented? | Ades et al. (2013) |
| 188 | Adequacy of Information on Model Specification and Software Implementation | Ades et al. (2013) |
| 189 | (1) methodological validity | Alexandrov et al. (2009) |
| 190 | Are the methods appropriate for the question asked? | Allen and Ho (2017) |
| 191 | If it is an observational study, did the analysis attempt to account for confounders using appropriate statistical techniques? | Allen and Ho (2017) |
| 192 | Methods: - Is the research design strong? | Allen (2014) |
| 193 | Methods: - Are the methods sufficiently described so that the study could be replicated by another researcher? | Allen (2014) |
| 194 | Clarity/validity of study design & data selection | Alexander (2005) |
| 195 | Are the methods appropriate and presented in sufficient detail to allow the results to be repeated? | Benos et al. (2003) |
| 196 | Is the design of the study clear? | Brand (2012) |
| 197 | Is the design (including methods) appropriate and adequately rigorous to answer the question or test the hypothesis? Is the question answerable or the hypothesis addressable with the study design? | Brand (2012) |
| 198 | In clinical studies, are all inclusion and exclusion criteria described and appropriate to the question(s)? In therapeutic studies (including surgery), are indications and contraindications for the treatments clearly described? | Brand (2012) |
| 199 | Do the authors have adequate numbers of patients, animals, or specimens to address the questions or purposes? In the case of rare conditions or rare events where a single study cannot likely address the questions, can the study materially contribute to some future systematic review or meta-analysis? Have they performed an a priori power analysis where there are multiple cohorts or groups? | Brand (2012) |
| 200 | In clinical studies with more than one group, are the groups demonstrably comparable (with use of proper statistics where appropriate)? | Brand (2012) |
| 201 | Are the controls adequately described? Are they appropriate controls? | Brand (2012) |
| 202 | Are all critical methods of assessment of dependent variables valid and reproducible? Are they described in adequate detail? Could another observer reproduce the data based on the description? Do the authors fully disclose sources and methods? | Brand (2012) |
| 203 | Do the authors justify the choice of statistical tests (considering assumptions of each)? | Brand (2012) |
| 204 | Could the reader use the methods section as a guide to replicate the study? | Christenbery (2011) |
| 205 | How clear is the study design? | Christenbery (2011) |
| 206 | Is there an adequate description of the setting and subjects? | Christenbery (2011) |
| 207 | Has the author provided rationale for the methodological choices (i.e., design, recruitment strategies, data analyses)? | Christenbery (2011) |
| 208 | Are the operational definitions of the variables clear? | Christenbery (2011) |
| 209 | Is the method used to collect data appropriate for the identified qualitative focus? | Christenbery (2011) |
| 210 | Is the type of sampling method identified (e.g., snowballing, purposive, convenience) and clearly described? | Christenbery (2011) |
| 211 | Was the type of data collection procedure identified (e.g., unstructured or semistructured interviews, oral histories, focus groups) and clearly described? | Christenbery (2011) |
| 212 | What types of observational and field notes were used? | Christenbery (2011) |
| 213 | Was data saturation discussed? | Christenbery (2011) |
| 214 | Were appropriate analytic procedures clearly described for the qualitative tradition (e.g., phenomenology, ethnographic, grounded theory) that was used? | Christenbery (2011) |
| 215 | Are steps taken to ensure: *Credibility* (e.g., researcher’s credentials); *Confirmability* (e.g., bracketing of researcher bias); *Dependability* (e.g., detailed description of research methods) | Christenbery (2011) |
| 216 | Sufficient details to allow the results to be repeated | Duchesne (2008) |
| 217 | Study type (retrospective or prospective) | Duchesne (2008) |
| 218 | Data collection setting(s) and location(s) | Duchesne (2008) |
| 219 | Information on participant recruitment, sampling, and allocation | Duchesne (2008) |
| 220 | Description of study population, inclusion and exclusion criteria | Duchesne (2008) |
| 221 | Clinical and demographic characteristics | Duchesne (2008) |
| 222 | Flow of participants through each stage | Duchesne (2008) |
| 223 | Final group numbers for analysis | Duchesne (2008) |
| 224 | Methods-Study population: Reference standard | Duchesne (2008) |
| 225 | Clinical assumptions on participants and data sets | Duchesne (2008) |
| 226 | Sequence and image parameters | Duchesne (2008) |
| 227 | Scanner type, make, manufacturer | Duchesne (2008) |
| 228 | Sources of imaging and data noise | Duchesne (2008) |
| 229 | Other materials and devices | Duchesne (2008) |
| 230 | Input parameters to the method | Duchesne (2008) |
| 231 | Units, cutoffs, parameters | Duchesne (2008) |
| 232 | Formulas and algorithms | Duchesne (2008) |
| 233 | Implemented hardware and software | Duchesne (2008) |
| 234 | Number, training and expertise of raters | Duchesne (2008) |
| 235 | Outcome measures and methods | Duchesne (2008) |
| 236 | Sample size | Duchesne (2008) |
| 237 | Methods - Methods: Expected result or model, considering sample size | Duchesne (2008) |
| 238 | Validation criterion | Duchesne (2008) |
| 239 | Validation objective | Duchesne (2008) |
| 240 | Methods - Methods: Type, number and characteristics of validation data sets | Duchesne (2008) |
| 241 | Methods - Methods: Sensitivity analysis to parameters | Duchesne (2008) |
| 242 | Do the authors clearly explain data collection, processing, and division methods? | England and Cheng (2019) |
| 243 | Do the data appropriately represent the range of possible patients and disease manifestations? | England and Cheng (2019) |
| 244 | Are the data labels (if applicable) of sufficient quality to support the claimed performance of the algorithm or algorithms? | England and Cheng (2019) |
| 245 | Do the authors report a sufficient number and type of performance measures to accurately represent strengths and weaknesses of the algorithms? | England and Cheng (2019) |
| 246 | Are performance measures reported with confidence intervals? | England and Cheng (2019) |
| 247 | If expert-level performance is claimed, does the standard of comparison meet an appropriate level of expertise? | England and Cheng (2019) |
| 248 | Are the methods and analysis valid and clear? | Estrada et al. (2006) |
| 249 | Do the experiments test the hypothesis? | Heddle and Ness (2009) |
| 250 | Suggest additional experiments only if they are necessary to provide additional support for the conclusions | Heddle and Ness (2009) |
| 251 | Are the methods clearly stated and appropriate? | Heddle and Ness (2009) |
| 252 | Does the Methods section provide sufficient detail that the experiments could be repeated? Are the statistical analyses appropriate? Nowadays, much, even most, of the Methods is relegated to online status, and it is very easy for reviewers to simply skip over that critical component of the paper. | Hill (2018) |
| 253 | Is the study design clear? | Kotsis and Chung (2018) |
| 254 | Was it the appropriate study design for the research objectives? | Kotsis and Chung (2018) |
| 255 | Are the methods sufficiently detailed so that someone else could repeat what was done? | Kotsis and Chung (2018) |
| 256 | Was the source of subjects discussed? | Kotsis and Chung (2018) |
| 257 | Are inclusion and exclusion criteria of the sample discussed? | Kotsis and Chung (2018) |
| 258 | Did the authors conduct an a priori power analysis? | Kotsis and Chung (2018) |
| 259 | If more than one sample is used (ie. cases and controls), were they similar? Was this tested statistically? | Kotsis and Chung (2018) |
| 260 | Were subjects randomized? If so, was the method of randomization described? | Kotsis and Chung (2018) |
| 261 | Are the results generalizable based on the sample? | Kotsis and Chung (2018) |
| 262 | Was blinding used and discussed? | Kotsis and Chung (2018) |
| 263 | Were the treatments well defined? | Kotsis and Chung (2018) |
| 264 | Were the correct measurements used? (ie. Objective functional measurements) Is there great measurement error? | Kotsis and Chung (2018) |
| 265 | Were the outcome measures appropriate? (ie. Validated questionnaires to assess subjective measurements such as pain) | Kotsis and Chung (2018) |
| 266 | Was the duration of follow-up discussed? | Kotsis and Chung (2018) |
| 267 | Were the proper statistical tests conducted? Are they clearly stated? | Kotsis and Chung (2018) |
| 268 | Were p-values and/or confidence intervals reported? | Kotsis and Chung (2018) |
| 269 | Are losses to follow-up/missing data/low response rate reported and discussed? | Kotsis and Chung (2018) |
| 270 | Who is it about? (Population/Patients/Participants): Subject Recruitment (Dates, places) | Kyrgidis and Triaridis (2010) |
| 271 | Exclusion-inclusion criteria (description of patients) | Kyrgidis and Triaridis (2010) |
| 272 | Generalizibility to “real life” (reference population of the study sample) | Kyrgidis and Triaridis (2010) |
| 273 | Take notes of subjects, groups and places. | Kyrgidis and Triaridis (2010) |
| 274 | Was the design of the study sensible? (Study design): Study design (descriptive: case reports, case series, analytic: case-control, cohorts, clinical trials) | Kyrgidis and Triaridis (2010) |
| 275 | Intervention (observation? Therapeutic?) | Kyrgidis and Triaridis (2010) |
| 276 | Outcome measured, how? (primary and secondary outcomes) | Kyrgidis and Triaridis (2010) |
| 277 | Take notes of design, intervention, outcome. | Kyrgidis and Triaridis (2010) |
| 278 | Was the study adequately controlled?: Randomisation truly random? (quasi-random?, random?, sequential allocation?) | Kyrgidis and Triaridis (2010) |
| 279 | If non randomized, were controls appropriate? (Matched?, cohort?) | Kyrgidis and Triaridis (2010) |
| 280 | Were the groups comparable? (Age, sex, baseline condition, therapeutic interventions) | Kyrgidis and Triaridis (2010) |
| 281 | Avoidance of potential sources of bias. | Kyrgidis and Triaridis (2010) |
| 282 | Take notes for control matching and possible biases. | Kyrgidis and Triaridis (2010) |
| 283 | Provide exhaustive comments: Materials and methods accurately described | Lippi (2018) |
| 284 | Sufficient sample size? | Lippi (2018) |
| 285 | Accurate methods and appropriate statistical tests? | Lippi (2018) |
| 286 | Study reproducible? | Lippi (2018) |
| 287 | Is the sample and its formation described in detail? | Marušic et al. (2005) |
| 288 | Are inclusion and exclusion criteria stated? | Marušic et al. (2005) |
| 289 | Is there a study flowchart? | Marušic et al. (2005) |
| 290 | Are the methods supported by references? | Marušic et al. (2005) |
| 291 | Did the research question match the method used? | Oerther and Watson (2019) |
| 292 | Did the authors use appropriate procedures used to safeguard the rights of study participants? | Oerther and Watson (2019) |
| 293 | Was the study externally reviewed and approved by an IRB/ethics review board? | Oerther and Watson (2019) |
| 294 | If the study involved an intervention with human participants, was it appropriately prospectively registered in accord with AllTrials principles or WHO requirements (Noyes, 2018)? | Oerther and Watson (2019) |
| 295 | Were the data collection points appropriate? | Oerther and Watson (2019) |
| 296 | Did the design minimize biases and threats to the validity of the study? | Oerther and Watson (2019) |
| 297 | Was the population described in adequate detail? | Oerther and Watson (2019) |
| 298 | Did the authors try to minimize sampling biases? | Oerther and Watson (2019) |
| 299 | Was the sample size based on a power analysis? | Oerther and Watson (2019) |
| 300 | Was there congruency between the operational and conceptual definitions? | Oerther and Watson (2019) |
| 301 | Did the authors use appropriate methods to measure key variables? | Oerther and Watson (2019) |
| 302 | Did the authors adequately described specific instruments, and were they good choices, given the study population and the variables being studied? | Oerther and Watson (2019) |
| 303 | Did the authors provide evidence that the data collection methods yielded data that were reliable and valid? | Oerther and Watson (2019) |
| 304 | Did the authors provide evidence that the staff who collected the data were adequately trained? | Oerther and Watson (2019) |
| 305 | Is methodology appropriate? Is it adequately described? | Paice (2001) |
| 306 | Is there in-built bias? Is the sample large enough? | Paice (2001) |
| 307 | Could another investigator reproduce the study using the methods as outlined or are the methods unclear? | Provenzale and Stanley (2005) |
| 308 | Do the authors justify any choices available to them in their study design (e.g., choices of imaging techniques, analytic tools, or statistical methods)? | Provenzale and Stanley (2005) |
| 309 | If the authors have stated a hypothesis, have they designed methods that could reasonably allow their hypothesis to be tested? | Provenzale and Stanley (2005) |
| 310 | Are the inclusion and exclusion criteria stated clearly? | Rosenfeld (2010) |
| 311 | Do the inclusion criteria fairly represent the intended target population? | Rosenfeld (2010) |
| 312 | Do the exclusion criteria fairly represent subjects to whom results should not apply? | Rosenfeld (2010) |
| 313 | Is the sampling method and recruitment period (start and end) stated clearly? | Rosenfeld (2010) |
| 314 | If the study sample is not consecutive or systematic, do the investigators deal with bias that may result from convenience or judgmental sampling? | Rosenfeld (2010) |
| 315 | Are interventions described with enough detail for repetition by the reader? | Rosenfeld (2010) |
| 316 | Does achieving results similar to the investigators’ require a level of expertise, experience, or technology that is unavailable to most readers? | Rosenfeld (2010) |
| 317 | Have all adjunctive therapies or interventions been accounted for so the reader may distinguish their effects from those of the primary intervention? | Rosenfeld (2010) |
| 318 | Is the study design appropriate for the stated research objectives? | Rosenfeld (2010) |
| 319 | If the study is observational, what precautions were taken to reduce bias? | Rosenfeld (2010) |
| 320 | Was a control or comparison group used, and, if not, should it have been? | Rosenfeld (2010) |
| 321 | Are the tests, surveys, and outcome measures appropriate, valid, and unbiased? | Rosenfeld (2010) |
| 322 | Are specific methods described in adequate detail? | Rosenfeld (2010) |
| 323 | If appropriate, do the authors include a sample size calculation? | Rosenfeld (2010) |
| 324 | For “significant” findings, do the authors also describe effect size (e.g., odds ratio, relative risk, correlation coefficient) and discuss clinical importance? | Rosenfeld (2010) |
| 325 | If there are less than 20 observations per group, do the authors check the data distribution for asymmetry or outliers that warrant nonparametric or exact tests? | Rosenfeld (2010) |
| 326 | When 3 or more groups are compared, do the authors first test for a global difference (e.g., analysis of variance) before making pairwise comparisons? | Rosenfeld (2010) |
| 327 | When multiple factors are related to an outcome, do the authors use regression analysis to avoid the false positive problem of multiple individual tests? | Rosenfeld (2010) |
| 328 | Are the study design, conduct, and analysis described in a manner that is unbiased, appropriate, and reproducible? | Rosenfeld (2010) |
| 329 | Was the study sample chosen appropriately and described in adequate detail for the results to be generalized? | Rosenfeld (2010) |
| 330 | Eligibility criteria for participants | Rostami et al. (2011) |
| 331 | How sample size was determined | Rostami et al. (2011) |
| 332 | Dates defining the periods of recruitment and follow-up | Rostami et al. (2011) |
| 333 | Baseline demographic and clinical characteristics of each group | Rostami et al. (2011) |
| 334 | Method used to generate the random allocation sequence | Rostami et al. (2011) |
| 335 | Precise details of the interventions intended for each group and how and when they were actually administered | Rostami et al. (2011) |
| 336 | Clearly defined primary and secondary outcome measures | Rostami et al. (2011) |
| 337 | Research question: appropriately answered? | Rostami et al. (2011) |
| 338 | Overall design of study: Adequate, relevant | Rostami et al. (2011) |
| 339 | Participants studied: Adequately described and their conditions defined? | Rostami et al. (2011) |
| 340 | Methods: Adequately described? Ethical issues discussed? | Rostami et al. (2011) |
| 341 | Methodology - study design and methodology (are the quantitative/qualitative methods adequately used? Has the study design been described precisely? Is the design suitable for answering the research query? Are sample size/selection adequate? Are appropriate instruments/methods of analysis used?) | Schuttpelz-Brauns et al. (2010) |
| 342 | Methods: Are the subjects adequately described (i.e. do you know everything you need to for proper interpretation of the results)? | Seals and Tanaka (2000) |
| 343 | Methods: Is the subject population appropriate for the question posed? | Seals and Tanaka (2000) |
| 344 | Methods: Is the number of subjects sufficiently large to provide the necessary statistical power to show a difference if it is really present (i.e., minimize the likelihood of producing a type II error)? | Seals and Tanaka (2000) |
| 345 | Methods: Will the subject population allow extensive or rather limited generalizability? | Seals and Tanaka (2000) |
| 346 | Methods: Was the assignment of subjects to conditions randomized? | Seals and Tanaka (2000) |
| 347 | Methods: Are proper control groups and/or conditions included? | Seals and Tanaka (2000) |
| 348 | Methods: Does the experimental design allow the hypothesis to be tested in a rigorous scientific manner? Is there a better experimental approach that could have been employed? | Seals and Tanaka (2000) |
| 349 | Methods: Does the experimental design and the protocols employed control for all potential confounding factors? Stated another way, does the experimental approach effectively isolate the mechanism or factor of interest? | Seals and Tanaka (2000) |
| 350 | Methods: Was each methodology described in sufficient detail for others to repeat the study? If not, do the authors provide a proper (i.e., peer reviewed) references that would provide such details? | Seals and Tanaka (2000) |
| 351 | Methods: Are the measurement techniques used sufficiently reliable, precise, and valid? | Seals and Tanaka (2000) |
| 352 | Methods: Is the rationale for making each measurement either obvious or explained? | Seals and Tanaka (2000) |
| 353 | Methods: Have the data been analyzed in the most appropriate manner? Were the investigators properly "blinded" in the analysis to eliminate possible bias? | Seals and Tanaka (2000) |
| 354 | Methods: Are the details as to how data were derived (calculated) adequately explained so that they can be confirmed by the reviewer and reproduced by future investigators? | Seals and Tanaka (2000) |
| 355 | Methods: Is it clear how the data will be interpreted to either support or refute the hypotheses? | Seals and Tanaka (2000) |
| 356 | Is the information provided sufficient to determine if the right methodology/protocol was followed? | Simpson (2008) |
| 357 | Was the testing order appropriate to avoid biasing due to the order that the conditions were tested in? | Simpson (2008) |
| 358 | Were appropriate research design methods used? | Simpson (2008) |
| 359 | Were the data appropriately reduced/calculated/analyzed? Correct variables selected to test the anticipated outcomes? | Simpson (2008) |
| 360 | Bottom line: Considering all of the methods used, are the validity and reliability of the data acceptable? | Simpson (2008) |
| 361 | For nonsignificant findings, do authors explore reasons? | Simpson (2008) |
| 362 | Is the methodology well thought out and appropriate for the study design? | Smith et al. (2018) |
| 363 | Is each step in the research specifically described in an understandable way? | Smith et al. (2018) |
| 364 | Are the inclusion/exclusion criteria specifically stated and appropriate? | Smith et al. (2018) |
| 365 | Are any items missing? | Smith et al. (2018) |
| 366 | Could I replicate the study based solely upon this description? | Smith et al. (2018) |
| 367 | Is the *Materials and methods* section structured? | Smolčić and Simundić (2014) |
| 368 | Are the following subheading used (if applicable): *Study design, Subjects, Blood sampling, Methods, Statistical analysis*? | Smolčić and Simundić (2014) |
| 369 | Do the authors correctly indicate the type of study (e.g. observational, prospective, retrospective, diagnostic accuracy, analytical validation)? | Smolčić and Simundić (2014) |
| 370 | Do the authors indicate the number of groups and patients within groups? | Smolčić and Simundić (2014) |
| 371 | Are precise inclusion and exclusion criteria provided? | Smolčić and Simundić (2014) |
| 372 | Are criteria for diseases and conditions clearly defined and referenced (if applicable)? | Smolčić and Simundić (2014) |
| 373 | Is the control group described in sufficient detail? | Smolčić and Simundić (2014) |
| 374 | Is the method of recruitment described adequately? | Smolčić and Simundić (2014) |
| 375 | Do the authors clearly state how they determined the absence of disease in control individuals? | Smolčić and Simundić (2014) |
| 376 | Was blood sampling performed in the fasting state? | Smolčić and Simundić (2014) |
| 377 | What tubes, additives and volumes were used for blood sampling? | Smolčić and Simundić (2014) |
| 378 | How many tubes were used for blood sampling? | Smolčić and Simundić (2014) |
| 379 | Was blood testing performed immediately or were samples aliquoted and stored? | Smolčić and Simundić (2014) |
| 380 | Is manufacturer information provided for all reagents and equipment used? | Smolčić and Simundić (2014) |
| 381 | Do the authors list all tests performed in the study? Were all the tests listed in the *Materials and Methods* section actually performed, and are results provided in the *Results* section? | Smolčić and Simundić (2014) |
| 382 | Are the methods explained in sufficient detail? | Smolčić and Simundić (2014) |
| 383 | Are any non-standard methods described in sufficient detail? | Smolčić and Simundić (2014) |
| 384 | Is CV provided for non-standard methods, such as ELISA assays, new biomarkers, or assays for specific proteins? Do the authors clearly state whether the CV they report is based on manufacturer specifications or their own measurements? | Smolčić and Simundić (2014) |
| 385 | Are QC measures explained (if applicable)? | Smolčić and Simundić (2014) |
| 386 | Adequacy of study design and methodology, including appropriate rating of the level of evidence | Stahel and Moore (2016) |
| 387 | How was the study performed?: What is the study design? | Stahel and Moore (2016) |
| 388 | Are the outcome measures and analytical methods appropriate? | Stahel and Moore (2016) |
| 389 | Are the methods and statistical analyses designed to answer the research question(s)? | Stone et al. (2018) |
| 390 | Were there any fatal flaws with the methodology? Fatal flaw may be described as an error that could not be remedied through a writing revision. | Stone et al. (2018) |
| 391 | Are primary and secondary outcomes clearly stated? | Sucato and Holland-Hall (2018) |
| 392 | Are confounding variables considered and accounted for? | Sucato and Holland-Hall (2018) |
| 393 | Does the primary outcome appropriately address the aim of the study? | Sucato and Holland-Hall (2018) |
| 394 | For hypothesis-driven studies, was a power analysis performed to determine the necessary sample size to answer the question? | Sucato and Holland-Hall (2018) |
| 395 | Are the statistical tests used appropriate? | Sucato and Holland-Hall (2018) |
| 396 | Are inclusion and exclusion criteria clearly stated? | Sucato and Holland-Hall (2018) |
| 397 | Are the methods sufficiently described that the study could be replicated? | Sucato and Holland-Hall (2018) |
| 398 | For retrospective studies or database queries, how were cases identified and collected? | Sucato and Holland-Hall (2018) |
| 399 | How were subjects recruited? | Sucato and Holland-Hall (2018) |
| 400 | Is the research design clear and appropriate to answer the authors' question(s)? | Sucato and Holland-Hall (2018) |
| 401 | Is the study population clearly described? | Sucato and Holland-Hall (2018) |
| 402 | Was a control group used? Were they appropriately similar to the intervention group(s)? | Sucato and Holland-Hall (2018) |
| 403 | Were reliable and validated measurement instruments (surveys, screening tests, etc) used? | Sucato and Holland-Hall (2018) |
| 404 | Overall: (c) Quality of the work. | Tandon (2014) |
| 405 | Is the type of study design specified? | Tandon (2014) |
| 406 | Are the methods clearly described in terms of inclusion/exclusion criteria, procedures or tests used, measurements utilized, primary and secondary outcomes or independent and dependent variables, statistical analysis utilized? The methods should be stated in a way that would allow another investigator to precisely reproduce the study. | Tandon (2014) |
| 407 | If the authors have stated a hypothesis, are the designed methods appropriate to reasonably test the hypothesis? | Tandon (2014) |
| 408 | Was the assignment of patients to treatment randomized? | Walker (1997) |
| 409 | Was the research design fundamentally sound? | Walker (1997) |
| 410 | Were all patients entered into the trial properly accounted for and attributed at its conclusion?: Was patient selection appropriate? | Walker (1997) |
| 411 | Was follow up complete? (Patients) | Walker (1997) |
| 412 | Were patients analyzed in the groups to which they were assigned? | Walker (1997) |
| 413 | Were the patients blinded to which treatment they received? | Walker (1997) |
| 414 | Were clinicians blinded to treatment given? | Walker (1997) |
| 415 | Were other key study personnel blinded as well? | Walker (1997) |
| 416 | Were both groups similar at baseline?: Demographic data (table?) | Walker (1997) |
| 417 | Prognostic factors (stratified?) (Group difference) | Walker (1997) |
| 418 | General health questions? (Group difference) | Walker (1997) |
| 419 | If not, were the statistics adjusted to account for this? | Walker (1997) |
| 420 | Was the eligibility criteria for the entry to the trial appropriate? | Walker (1997) |
| 421 | Was the sample size calculated before trial commencement? | Walker (1997) |
| 422 | Were all spectrums of disease represented in the sample? | Walker (1997) |
| 423 | Were the interventions used appropriate?: Were they sensible and described adequately? | Walker (1997) |
| 424 | Are they affordable? (intervention) | Walker (1997) |
| 425 | Are they available? (intervention) | Walker (1997) |
| 426 | Was there a placebo used? (intervention) | Walker (1997) |
| 427 | Was there a non-treatment group (natural history)? (intervention) | Walker (1997) |
| 428 | Were the groups “treated equally”? | Walker (1997) |
| 429 | Was there any co-intervention? | Walker (1997) |
| 430 | Was there any contamination? | Walker (1997) |
| 431 | Did patients comply with treatment regimens and instructions? | Walker (1997) |
| 432 | Were the instruments of measurement used a reasonable and adequate for all clinically important outcomes? | Walker (1997) |
| 433 | Were the instruments of measurement used reliable and valid? | Walker (1997) |
| 434 | Was normal defined? | Walker (1997) |
| 435 | Was there sufficient follow up? | Walker (1997) |
| 436 | Were adverse effects documented? | Walker (1997) |
| 437 | How large was the treatment effect?: Was it statistically significant? | Walker (1997) |
| 438 | Was it clinically significant? (treatment effect) | Walker (1997) |
| 439 | Was the difference biologically plausible? | Walker (1997) |
| 440 | How precise was the estimate of effect?: Was sufficient sample size used? | Walker (1997) |
| 441 | Were confidence intervals given? (precision of effect estimate) | Walker (1997) |
| 442 | Was there any data dredging? (precision of effect estimate) | Walker (1997) |
| 443 | Were there any other biases operating, and if so in what direction? | Walker (1997) |
| 444 | Are the methods of solution new? Can the proposed solution methods be used to solve other problems of interest? | Wilson (2002) |
| 445 | Protocol | Hunter (2020) |
| 446 | Inclusion & exclusion criteria | Hunter (2020) |
| 447 | Search strategy | Hunter (2020) |
| 448 | Screening | Hunter (2020) |
| 449 | Data extraction | Hunter (2020) |
| 450 | Quality appraisal & certainty of the evidence | Hunter (2020) |
| 451 | Did the review look for the right type of papers? | Hunter (2020) |
| 452 | Did the review's authors do enough to assess the quality of the included studies? | Hunter (2020) |
| 453 | Dependability: The extent to which the research could be replicated in similar conditions | Stenfors et al (2020) |
| 454 | Reflexibity: A continual process of engaging with and articulating the place of the researcher and the context of the research | Stenfors et al (2020) |
| 455 | Whether ethical clearance and informed consent/assent have been mentioned. | Tullu and Karande (2020) |
| 456 | Appropriate sampling procedures have been used. | Tullu and Karande (2020) |
| 457 | Whether the study participant selection (and description ), study design, randomization, blinding, data collection procedures and instruments, precise details of interventions, study treatments/procedures, study endpoints, and primary/secondary outcomes have been stated. | Tullu and Karande (2020) |
| 458 | Is the sample size adequate to provide the necessary statistical power to the study (minimize type II error)? | Tullu and Karande (2020) |
| 459 | Statistical test(s) are described and appropriate. | Tullu and Karande (2020) |
| 460 | Are trade names and symbols used properly? | Tullu and Karande (2020) |
| 461 | If diagnostic kits or statistical packages or questionnaires are used- whether details like owner/producer/trademark-/patent-/copyright-holder stated, and whether the city, year and permission to use is stated. | Tullu and Karande (2020) |
| 462 | Are the selection criteria and definitions appropriate? | Pai (2020) |
| 463 | Are the details sufficient? | Pai (2020) |
| 464 | Are the dates correct? | Pai (2020) |
| 465 | Are the hypothesis and the primary and secondary outcomes clear? | Pai (2020) |
| 466 | Is there a protocol to compare the manuscript with, to ensure these have not been changed since the research was conducted? | Pai (2020) |
| 467 | If the research is a clinical trial, was it registered? | Pai (2020) |
| 468 | Is the population studied appropriate to address the hypothesis? Or is there any selection bias? | Pai (2020) |
| 469 | Is there evidence for validity of the HRQOL measure in the applicable patient population? | Lapin (2020) |
| 470 | Were appropriate methods used to address missing data and compliance? | Lapin (2020) |
| 471 | Study design | Brown et al (2017) |
| 472 | Inclusion and exclusion criteria and time period (cohort) | Brown et al (2017) |
| 473 | define predictor variables | Brown et al (2017) |
| 474 | define primary and secondary outcomes | Brown et al (2017) |
| 475 | statistical analyses | Brown et al (2017) |
| 476 | institutional review board statement | Brown et al (2017) |
| Results | | |
| 477 | Risk of Bias: If a bias risk was identified, was any adjustment made to the analysis and was this adequately justified? | Ades et al. (2013) |
| 478 | Have numerical estimates been provided of the degree of heterogeneity in the relative treatment effects? | Ades et al. (2013) |
| 479 | Has a justification been given for choice of random or fixed effect models? Should sensitivity analyses be considered? | Ades et al. (2013) |
| 480 | Has there been an adequate response to heterogeneity? | Ades et al. (2013) |
| 481 | Does the extent of unexplained variation in relative treatment effects threaten the robustness of conclusions? | Ades et al. (2013) |
| 482 | Baseline Model for Trial Outcomes: Are baseline effects and relative effects estimated in the same model? If so, has this been justified? | Ades et al. (2013) |
| 483 | Baseline Model for Trial Outcomes: Has the choice of studies used to inform the baseline model been explained? | Ades et al. (2013) |
| 484 | Presentation of Results of Analyses of Trial Data: Are the relative treatment effects (relative to a placebo or ‘‘standard’’ comparator) tabulated, alongside measures of between-study heterogeneity if an RE model is used? | Ades et al. (2013) |
| 485 | Presentation of Results of Analyses of Trial Data: Are the absolute effects on each treatment, as they are used in the CEA, reported? | Ades et al. (2013) |
| 486 | Synthesis in Other Parts of the Natural History Model: Is the choice of data sources to inform the other parameters in the natural history model adequately described and justified? | Ades et al. (2013) |
| 487 | Synthesis in Other Parts of the Natural History Model: In the natural history model, can the longer-term differences between treatments be explained by their differences on randomized trial outcomes? | Ades et al. (2013) |
| 488 | Multiarm Trials: If there are multiarm trials, have the correlations between the relative treatment effects been taken into account? | Ades et al. (2013) |
| 489 | Connected and Disconnected Networks: Is the network of evidence based on randomized trials connected? | Ades et al. (2013) |
| 490 | Inconsistency: How many inconsistencies could there be in the network? | Ades et al. (2013) |
| 491 | Inconsistency: Are there any a priori reasons for concern that inconsistency might exist, due to systematic clinical differences between the patients in trials comparing treatments A and B, the patients in trials comparing treatments A and C, and so on? | Ades et al. (2013) |
| 492 | Inconsistency: Have adequate checks for inconsistency been made? | Ades et al. (2013) |
| 493 | Inconsistency: If inconsistency was detected, what adjustments were made to the analysis, and how was this justified? | Ades et al. (2013) |
| 494 | Uncertainty Propagation: Has the uncertainty in parameter estimates been propagated through the Cost Effectiveness Analysis model? | Ades et al. (2013) |
| 495 | Correlations: Are there correlations between parameters? If so, have the correlations been propagated through the CEA model? | Ades et al. (2013) |
| 496 | Adequacy of sample & data set | Alexander (2005) |
| 497 | Clarity/validity of measurement of key variables | Alexander (2005) |
| 498 | (6) if results support claims or conclusions | Alexandrov et al. (2009) |
| 499 | 3. Methodology—is it true?: c. Assess the external validity of the study findings. How generalizable are the study findings to other populations? | Allen and Ho (2017) |
| 500 | Results: - Do the results contain all outcome measures described in the methods? | Allen (2014) |
| 501 | Results: - Are raw data provided (not just summaries or percentages)? | Allen (2014) |
| 502 | 1. Scientific quality of the work_ Are the data adequate to support the conclusions? | Benos et al. (2003) |
| 503 | Results: 1. Are the observations clearly presented and in order of the questions, purposes, or hypotheses? (In good writing, key findings will be apparent by reading only each sentence in the first paragraph of the Results section.) | Brand (2012) |
| 504 | Results: 2. Does the presentation of the findings persuade the reader the data have been rigorously obtained and reported? Do the authors report variability of the data in an appropriate fashion? | Brand (2012) |
| 505 | Results: 3. Are all essential observations arising from the experimental design reported (ie, do the authors omit or inadequately present potentially critical data)? On the other hand, do the authors present only essential data for answering the questions? (In the case of vaguely worded purposes, many findings can be - and often are - presented which do not directly lead the reader to some coherent view or explanation or conclusion.) | Brand (2012) |
| 506 | Results: 4. Do the findings unambiguously answer the question(s) or address the purpose(s) or hypothesis(es)? | Brand (2012) |
| 507 | Reporting the findings _ Are the study’s findings clearly articulated? | Christenbery (2011) |
| 508 | Raw data reported as mean (standard deviation) or median (range) - | Duchesne (2008) |
| 509 | Results: Participants inclusion/exclusions | Duchesne (2008) |
| 510 | Results: Cross tabulation of test results by reference results | Duchesne (2008) |
| 511 | Results: Estimates of accuracy and measures of statistical uncertainty | Duchesne (2008) |
| 512 | Results: Measures of test reproducibility, if done | Duchesne (2008) |
| 513 | Results: Ancillary analyses, including sensitivity testing | Duchesne (2008) |
| 514 | If performance measures require an explicit threshold, do the authors provide convincing support for the threshold used? | England and Cheng (2019) |
| 515 | The Results section is the piece that arguably warrants the greatest amount of attention. The reviewer must determine whether the data are reliable and bolstered by requisite controls; whether appropriate scientific redundancy and rigor are applied throughout. Can some of the text be eliminated or condensed by judicious use of tables or figures? Are statistically significant findings also biologically significant? | Hill (2019) |
| 516 | Are the results clearly presented? | Kotsis and Chung (2035) |
| 517 | Is variability of the data discussed? | Kotsis and Chung (2036) |
| 518 | Do the tables and figures stand on their own? | Kotsis and Chung (2037) |
| 519 | Do the findings unambiguously answer the question or address the purpose or hypothesis? | Kotsis and Chung (2038) |
| 520 | Were side effects/complications reported? | Kotsis and Chung (2039) |
| 521 | Large enough, long enough, follow-up complete enough for adequate, credible results? | Kyrgidis and Triaridis (2010) |
| 522 | If negative results are presented (H0 accepted), ensure that study power is reported. | Kyrgidis and Triaridis (2010) |
| 523 | Provide exhaustive comments: Results section limited to relevant findings | Lippi (2018) |
| 524 | Clear description of results? | Lippi (2018) |
| 525 | Results: are they clear and convincing? Each table and figure has to be self-sufficient and carry a single message. | Marušic et al. (2005) |
| 526 | What was the outcome of the analysis? | Oerther and Watson (2019) |
| 527 | What was confirmed or discovered? | Oerther and Watson (2019) |
| 528 | Were the findings adequately summarized, with good use of tables and figures? | Oerther and Watson (2019) |
| 529 | Results Are they credible? Is the response rate adequate? | Paice (2001) |
| 530 | Are the results clearly explained? | Provenzale and Stanley (2005) |
| 531 | Does the order of presentation of the results parallel the order of presentation of the methods? | Provenzale and Stanley (2005) |
| 532 | Are the results reasonable and expected, or are they unexpected? | Provenzale and Stanley (2005) |
| 533 | Are there results that are introduced that are not preceded by an appropriate discussion in the Methods section? | Provenzale and Stanley (2005) |
| 534 | Clinical significance Do the authors account for uncertainty by providing a 95% confidence interval (CI) that gives a range of values considered plausible for the target population? | Rosenfeld (2010) |
| 535 | When the authors report “significant” or “positive” findings, is the lower limit of the 95% CI large enough to exclude a trivial or clinically unimportant outcome? | Rosenfeld (2010) |
| 536 | When the authors report “nonsignificant” or “negative” findings, is the upper limit of the 95% CI small enough to ensure a clinically important effect was not missed? | Rosenfeld (2010) |
| 537 | Adverse events If relevant, do the authors explicitly describe adverse events? | Rosenfeld (2010) |
| 538 | Are adverse events described by frequency and severity? | Rosenfeld (2010) |
| 539 | Does an uncontrolled study make unjustified claims of efficacy or effectiveness? | Rosenfeld (2010) |
| 540 | Do any problems exist with duration of follow-up, response rates, or dropouts? | Rosenfeld (2010) |
| 541 | Is survival analysis used for prospective studies with loss to follow-up or when events may not have occurred by study end (e.g., survival, recurrence)? | Rosenfeld (2010) |
| 542 | Inferential statistics Are claims of significant or important findings supported by statistical analysis? | Rosenfeld (2010) |
| 543 | Are paired or matched data (e.g., before and after) analyzed appropriately? | Rosenfeld (2010) |
| 544 | Results: Flow of participants through each stage | Rostami et al. (2011) |
| 545 | Results - Relevance, completeness, intelligibility, appropriate presentation of data, e.g. tables/graphics incl. legend (is the relevant data presented adequately? Do the graphics/tables agree with the text? Is the data appropriate to answering the question? Is the data presented in a comprehensible way and is it complete?) | Schuttpelz-Brauns et al. (2010) |
| 546 | Are the data reported in a clear, concise, and well-organized manner? | Seals and Tanaka (2000) |
| 547 | Are data presented on any measurement that was not described in the Methods? Alternatively, are the data on all measurements described in the Methods presented? | Seals and Tanaka (2000) |
| 548 | Have the data been presented in the appropriate units (e.g., absolute unit changes vs. percentage changes) or properly adjusted statistically (e.g., when there are differences in the baseline values of variables that could confound interpretation of the results)? | Seals and Tanaka (2000) |
| 549 | Do the data seem reasonable from a physiological perspective? | Seals and Tanaka (2000) |
| 550 | How do the group differences or responses shown compare with the measurement variability? | Seals and Tanaka (2000) |
| 551 | Are the data reported so that you can see for yourself the values and a corresponding measure of variability (e.g., standard deviation)? Except for judicious selection, values are not repeated in both text and in tables/figures? | Simpson (2008) |
| 552 | Are the data that you believe are important to answer the research question reported or should other outcomes also be reported? | Simpson (2008) |
| 553 | Do the data MAKE SENSE?? Are the values reasonable? Are the units correct (and in metric system)? Do the table values, graphs and/or text match? Are the degrees of freedom correct if reported? | Simpson (2008) |
| 554 | Was the study conducted appropriately per the author’s methodology? | Smith et al. (2018) |
| 555 | Are all patients that were enrolled accounted for? (e.g. patients screened vs. enrolled, withdrawn, or lost to follow-up) | Smith et al. (2018) |
| 556 | Are results included for all primary and secondary outcomes? | Smith et al. (2018) |
| 557 | Can the reviewer draw the same conclusions as the author? | Smith et al. (2018) |
| 558 | Results: Are the tests mentioned in the *Results* the same as those listed in the *Statistical analysis* section of *Materials and Methods*? | Smolčić and Simundić (2014) |
| 559 | Results: Do the authors explain any missing values? | Smolčić and Simundić (2014) |
| 560 | Results: Do the authors provide P values for all tested differences? | Smolčić and Simundić (2014) |
| 561 | Results: Do the authors refrain from using percentage if there are fewer than 100 subjects? | Smolčić and Simundić (2014) |
| 562 | Results: Do the authors refrain from using expressions like “effect” and “cause” if they have not performed an experiment? In the case of an observational study without intervention, do the authors limit themselves to talking only about associations? | Smolčić and Simundić (2014) |
| 563 | Results: Do the authors refrain from using expressions like “decline” and “increase” to describe the differences in concentrations between groups, and instead use those terms only to describe changes of one group through multiple measurements over a period of time? If the study is observational (whether case-control or cross-sectional), do the authors limit themselves to indicating only whether there is a difference between groups? | Smolčić and Simundić (2014) |
| 564 | The results should be presented in a logical, systematic fashion, with the presented data mirroring the same sequence as in the preceding methods section | Stahel and Moore (2016) |
| 565 | Analysis:, How were missing data handled? | Sucato and Holland-Hall (2018) |
| 566 | Analysis:, Was assessment of outcome variables subject to bias? | Sucato and Holland-Hall (2018) |
| 567 | Analysis, Should the manuscript be reviewed by a statistician? | Sucato and Holland-Hall (2018) |
| 568 | Results, Are the findings clinically and statistically significant? | Sucato and Holland-Hall (2018) |
| 569 | Results, Is there internal consistency between the data presented in the tables and that described in the text? | Sucato and Holland-Hall (2018) |
| 570 | Results, Were all recruited subjects accounted for at the end of the study? | Sucato and Holland-Hall (2018) |
| 571 | Results, Are measures of statistical significance reported (when indicated) for quantitative outcomes? | Sucato and Holland-Hall (2018) |
| 572 | Results, Is there excessive repetition between the text and the tables? | Sucato and Holland-Hall (2018) |
| 573 | Results, Are all analyses reported relevant to the study question(s)? | Sucato and Holland-Hall (2018) |
| 574 | Results, Do the authors refrain from interpreting their findings in this section? | Sucato and Holland-Hall (2018) |
| 575 | Has the author provided citations to support statements of fact? (check references) | Sylvia and Herbel (2001) |
| 576 | Has a critical finding been overlooked? (e.g., Is one of the safety and effectiveness features overemphasized? Are all patients accounted for in the study results?) | Sylvia and Herbel (2001) |
| 577 | Results, tables, and figures: (a) Are the results clearly explained? | Tandon (2014) |
| 578 | Results, tables, and figures: (b) Does the order of presentation of the results parallel the order of presentation of the methods? | Tandon (2014) |
| 579 | Can the results be applied to my patient care? | Walker (1997) |
| 580 | Search flow results | Hunter (2020) |
| 581 | Were all the important, relevant studies included? | Hunter (2020) |
| 582 | If different types of evidence, including indirect evidence are combined, or a meta-analysis was conducted, was it reasonable to do so? | Hunter (2020) |
| 583 | Credibility: The research findings are plausible and trustworthy | Stenfors et al (2020) |
| 584 | Whether well presented, clear, precise, and concise. | Tullu and Karande (2020) |
| 585 | All points raised in the methods are answered and results for all endpoints are stated in a logical sequence. | Tullu and Karande (2020) |
| 586 | Whether actual *P* values and 95% confidence intervals (CI) are reported. | Tullu and Karande (2020) |
| 587 | Whether the tables, figures, graphs, charts, and photographs are used appropriately and improve the readability of the manuscript. Have appropriate legends been given for these? Whether some tables can be simplified, condensed, or omitted. | Tullu and Karande (2020) |
| 588 | Whether appropriate units are used to describe the data. | Tullu and Karande (2020) |
| 589 | Whether the results are reported in relation to the specified aims and objectives. | Tullu and Karande (2020) |
| 590 | Whether the statistical analysis is adequate and appropriate. | Tullu and Karande (2020) |
| 591 | If supplementary files/materials are supplied, these also need to be reviewed by the reviewer. | Tullu and Karande (2020) |
| 592 | Is there any major flaw in the results? | Pai (2020) |
| 593 | Do the numbers match? Do the percentages match the numbers? Do the dates (if stated) and data match? | Pai (2020) |
| 594 | Have all relevant results been stated? | Pai (2020) |
| 595 | Are there lacunae in the data? | Pai (2020) |
| 596 | Are there supplemental files that you need to review? | Pai (2020) |
| 597 | Are the study subject characteristics well described? | Lapin (2020) |
| 598 | Is the magnitude of the HRQOL effect reported? | Lapin (2020) |
| 599 | Is the magnitude of the HRQOL effect clinically significant or biologically important? | Lapin (2020) |
| 600 | Are the implications of the HRQOL results on clinical practice properly interpreted and discussed? | Lapin (2020) |
| 601 | Cohort is assembled and data are abstracted after the primary outcome has already occurred (retrospective); Cohort is assembled and followed prospectively until the primary outcome occurs (prospective) | Brown et al (2017) |
| 602 | Results presented in same order as aims | Brown et al (2017) |
| 603 | Summarize results presented in tables without repeating them | Brown et al (2017) |
| Discussion | | |
| 604 | Risk of Bias: Is there a discussion of the biases to which these trials, or this ensemble of trials, are vulnerable? | Ades et al. (2013) |
| 605 | MCH program/policy implications addressed | Alexander (2005) |
| 606 | Is the discussion relevant? | Allen (2014) |
| 607 | Do the authors discuss their findings in the context of existing research? | Allen (2014) |
| 608 | Discussion (interpretation and synthesis of the results): 1. Are the assumptions, limitations, and source of bias adequately described? When describing the limitations, do the authors persuade the reader they do not seriously jeopardize the conclusions? | Brand (2012) |
| 609 | Discussion: 2. Do the authors adequately synthesize the observations with those in the literature? Are representative relevant past observations included in the Discussion, or do the authors selectively cite only a few and perhaps biased range of papers? Have the authors overlooked critical references? | Brand (2012) |
| 610 | Discussion: 3. Do the data support past observations or compare appropriately with published data? If not, do the authors adequately explore and explain any contradictions? | Brand (2012) |
| 611 | Discussion: 4. Does the synthesis of the authors’ data and that in the literature support all conclusions made by the authors? | Brand (2012) |
| 612 | Reporting the findings _ Does the author comment on the study’s findings in light of previous studies? | Christenbery (2011) |
| 613 | Reporting the findings _ Does the author explain any unexpected results? | Christenbery (2011) |
| 614 | Reporting the findings _ Are the findings linked to the theoretical or conceptual framework for the study? | Christenbery (2011) |
| 615 | Relevance to advanced practice nursing _ Does the author discuss how the study’s findings will improve patient care? | Christenbery (2011) |
| 616 | Implications for future studies _ Are there appropriate recommendations for future studies? | Christenbery (2011) |
| 617 | Implications for future studies _ Are suggestions for future studies provided? | Christenbery (2011) |
| 618 | Discussion - Claims: Clinical realism | Duchesne (2008) |
| 619 | Discussion - Claims: Claims supported by data | Duchesne (2008) |
| 620 | Discussion - Claims:Possible or actual bias, discrepancies | Duchesne (2008) |
| 621 | Discussion - Claims:Interpretation of results given hypotheses and sources of bias | Duchesne (2008) |
| 622 | Discussion - Claims:Generalization of findings, alternative explanation | Duchesne (2008) |
| 623 | Discussion - Innovation and impact:Innovation: transformational, translational, incremental | Duchesne (2008) |
| 624 | Discussion - Innovation and impact:Clinical applicability | Duchesne (2008) |
| 625 | Do the authors appropriately qualify the strength of their conclusions and discuss limitations in their methods? | England and Cheng (2019) |
| 626 | Do the authors discuss directions for future research? | England and Cheng (2019) |
| 627 | Is the discussion insightful? | Estrada et al. (2006) |
| 628 | Does the discussion: relate to the hypothesis? | Heddle and Ness (2009) |
| 629 | Does the discussion: : Over interpret or extrapolate inappropriately | Heddle and Ness (2009) |
| 630 | Does the Discussion put the findings in perspective, rather than simply rehashing what was described in the Results? Does it compare the reported findings with prior ones in the literature, highlighting and discussing commonalities and discrepancies? Does it provide an honest assessment of the inevitable limitations of the study? | Hill (2020) |
| 631 | Discussion does not duplicate previous information, appropriately discusses findings, conclusions are supported by biological explanations, study limitations are highlighted | Lippi (2018) |
| 632 | Discussion: does it begin with the most important finding? | Marušic et al. (2005) |
| 633 | Discussion: does it relate exclusively to the results of the study? | Marušic et al. (2005) |
| 634 | Was the issue of clinical significance discussed? | Oerther and Watson (2019) |
| 635 | How generalizable are the findings? | Oerther and Watson (2019) |
| 636 | Did the researchers discuss the implications of the study for clinical practice or further research? | Oerther and Watson (2019) |
| 637 | Discussion Are weaknesses and limitations explored? | Paice (2001) |
| 638 | Is the discussion concise? If not, how should it be shortened? | Provenzale and Stanley (2005) |
| 639 | If a hypothesis was proposed, do the authors state whether it was verified or falsified? Alternatively, if no hypothesis was proposed, do the authors state whether their research question was answered? | Provenzale and Stanley (2005) |
| 640 | Are the authors’ conclusions justified by the results found in the study? | Provenzale and Stanley (2005) |
| 641 | If there are unexpected results, do the authors adequately account for them? | Provenzale and Stanley (2005) |
| 642 | Do the authors note limitations of the study? Are there additional limitations that should be noted? | Provenzale and Stanley (2005) |
| 643 | Do the authors discuss clinical importance, not just statistical significance? | Rosenfeld (2010) |
| 644 | Do the authors discuss the relationship of benefit versus harm and adverse events? | Rosenfeld (2010) |
| 645 | If data were collected during routine clinical care, do the authors discuss the consistency, accuracy, availability, and completeness of source records? | Rosenfeld (2010) |
| 646 | If appropriate, is natural history or spontaneous improvement discussed? | Rosenfeld (2010) |
| 647 | Discussion - adequate interpretation of results, strengths/weaknesses analysis and limitations, relevance of results in relation to the question, plausibility of conclusions, relevance to conceptual framework (are the results interpreted adequately and are strengths and weaknesses considered sufficiently? Is the relevance of the results presented adequately? Are the conclusions plausible? Is there reference to the conceptual framework? Is the question/hypothesis/aim answered?) | Schuttpelz-Brauns et al. (2010) |
| 648 | Are the major new findings of the study clearly described and properly emphasized? | Seals and Tanaka (2000) |
| 649 | Is there any other way to interpret and/or explain the data other than that suggested by the authors? | Seals and Tanaka (2000) |
| 650 | Are important experimental observations from previous reports described in the context of the present results? | Seals and Tanaka (2000) |
| 651 | Do the authors support their statements with appropriate references? x Do the authors discuss their data in a manner that provides insight beyond that presented in previous sections? | Seals and Tanaka (2000) |
| 652 | Are the unique aspects and other experimental strengths of the study properly highlighted? | Seals and Tanaka (2000) |
| 653 | Do the authors make suggestions as to how the results of their study need to be extended in the future to learn more about the issue in question? | Seals and Tanaka (2000) |
| 654 | Does the author use the results of the study to support whether the expected outcomes occurred or not? | Simpson (2008) |
| 655 | Does the author use in the Discussion the justifications for the anticipated outcomes presented in the Introduction? Are other explanations of the outcomes present that should be explored? | Simpson (2008) |
| 656 | Are the interpretations that are presented supported by the data and/or previous studies and scientific concepts? | Simpson (2008) |
| 657 | If other relevant studies exist in this area, does author contrasts those findings to those of current study? | Simpson (2008) |
| 658 | Discussion: Does the author assess the results in context of pertinent existing literature? | Smith et al. (2018) |
| 659 | Discussion: Is the discussion focused and succinct? | Smith et al. (2018) |
| 660 | Discussion: Are the discussion points complementary to the results or do they simply reiterate the findings? | Smith et al. (2018) |
| 661 | Discussion: Does the author definitively state an answer to the original research question? | Smith et al. (2018) |
| 662 | Discussion: Does the *Discussion* start by listing the key results of the study? | Smolčić and Simundić (2014) |
| 663 | Discussion: Do the authors comment on their results and how they support or fail to support their hypothesis? | Smolčić and Simundić (2014) |
| 664 | Discussion: Do the authors discuss other studies and how they relate to their findings? | Smolčić and Simundić (2014) |
| 665 | Discussion: Do the authors discuss causal relationship only if their study was interventional and otherwise limit themselves to talking only about associations? | Smolčić and Simundić (2014) |
| 666 | Discussion: Do the authors indicate the added value of their work? Do they indicate what is new in their study and why this study is important? | Smolčić and Simundić (2014) |
| 667 | Discussion: Do the authors draw clear and unambiguous conclusions based solely on their results? | Smolčić and Simundić (2014) |
| 668 | Discussion: Do the authors clearly describe the limitations of their study? | Smolčić and Simundić (2014) |
| 669 | Discussion and Overall Writing Quality: Do the authors make sound judgments based on the data available? | Stone et al. (2018) |
| 670 | Discussion and Overall Writing Quality: If speculations are made, are they reasonable? | Stone et al. (2018) |
| 671 | Discussion and Overall Writing Quality: Did the authors compare and refute the current data to previously published works? | Stone et al. (2018) |
| 672 | Discussion and Overall Writing Quality: Note any major issues with writing mechanics, length of the submission (too brief or verbose), and readability. | Stone et al. (2018) |
| 673 | Discussion, Are areas for future research discussed? | Sucato and Holland-Hall (2018) |
| 674 | Discussion, Do the authors compare and contrast their findings with the work of others? Do they offer possible explanations of conflicting findings? | Sucato and Holland-Hall (2018) |
| 675 | Discussion, Do the authors refrain from overgeneralizing their findings (eg, to populations other than the one studied)? | Sucato and Holland-Hall (2018) |
| 676 | Differential diagnoses need to be separated by a sub-subheader in the differential diagnosis section. | Talanow (2014) |
| 677 | Discussion: (a) Is the discussion concise and clear? | Tandon (2014) |
| 678 | Discussion: (b) Is there a clear statement about the principal study findings? | Tandon (2014) |
| 679 | Discussion: (c) Is it clear what new knowledge the study has provided? | Tandon (2014) |
| 680 | Discussion: (d) Is it clear how the study findings ‘‘fit’’ or ‘‘don’t fit’’ with the existing literature? | Tandon (2014) |
| 681 | Discussion: (e) How are discrepant findings explained? | Tandon (2014) |
| 682 | Discussion: (f) Are the strengths and weaknesses of the study noted? | Tandon (2014) |
| 683 | Are there gaps in the discussion of the experimental methods or results? If there are such gaps, can the closing of these gaps be considered (i) essential, (ii) desirable, or (iii) interesting? Are the experimental methods described in sufficient detail so that other investigators can reproduce the experiments? | Wilson (2002) |
| 684 | Study limitations addressed | Alexander (2005) |
| 685 | Have the study’s limitations and weaknesses been identified? | Allen (2014) |
| 686 | Reporting the findings _ Has the author noted the study’s limitations? | Christenbery (2011) |
| 687 | Are the limitations discussed in enough detail? | Estrada et al. (2006) |
| 688 | Does the discussion: include limitations? | Heddle and Ness (2009) |
| 689 | Discussion: are the limitations of the study clearly stated? | Marušic et al. (2005) |
| 690 | Are the important experimental limitations of the study described so that the reader will be able to interpret the findings appropriately? | Seals and Tanaka (2000) |
| 691 | Does the author recognize limitations to the study? | Simpson (2008) |
| 692 | Discussion: Does the author include study limitations and unexpected results? | Smith et al. (2018) |
| 693 | Discussion, Are limitations of the study acknowledged? | Sucato and Holland-Hall (2018) |
| 694 | Were the limitations of the study discussed adequately? | Walker (1997) |
| 695 | Have the authors explicitly addressed the limitations of their study? | Wilson (2002) |
| 696 | Did the review address a clearly focused question? | Hunter (2020) |
| 697 | Are the evidence statements/recommendations in the Brief Overview, Verdict and Clinical Significance supported by the results? | Hunter (2020) |
| 698 | Confirmability: There is a clear link or relationship between the data and the findings | Stenfors et al (2020) |
| 699 | Transferability: Findings may be transferred to another setting, context or group | Stenfors et al (2020) |
| 700 | Whether it summarizes findings and explains the meaning of the main result. | Tullu and Karande (2020) |
| 701 | Emphasizes the new and important aspects of the study. | Tullu and Karande (2020) |
| 702 | Is in relation to the objectives/hypothesis. | Tullu and Karande (2020) |
| 703 | Answers “how” and “why” of the manuscript. | Tullu and Karande (2020) |
| 704 | Whether the literature search is adequate, and comparison is appropriately done. | Tullu and Karande (2020) |
| 705 | Whether the interpretation of results has been given. | Tullu and Karande (2020) |
| 706 | Is the result clinically important? | Tullu and Karande (2020) |
| 707 | Whether the main conclusion/s are explained and supported by the results. | Tullu and Karande (2020) |
| 708 | Whether it explores plausible explanation/s for conflicting results. | Tullu and Karande (2020) |
| 709 | Whether generalizability of results has been stated. | Tullu and Karande (2020) |
| 710 | Practical implications, strengths of the study as well as the limitations and biases have been discussed. | Tullu and Karande (2020) |
| 711 | Directions for future research have been stated. | Tullu and Karande (2020) |
| 712 | Whether the conclusion is rational and take-home/key message has been given. | Tullu and Karande (2020) |
| 713 | Clinical relevance to be rated good/fair or adequate/poor. | Tullu and Karande (2020) |
| 714 | Is there any major flaw in the discussion? | Pai (2020) |
| 715 | Are there loose statements in the discussion? | Pai (2020) |
| 716 | Is the discussion appropriate? | Pai (2020) |
| 717 | Is it too long? | Pai (2020) |
| 718 | Are all issues mentioned in the discussion relevant to the data presented? | Pai (2020) |
| 719 | Are there too many generalisations? | Pai (2020) |
| 720 | Does the discussion flow well? Are the arguments made in the discussion logical? | Pai (2020) |
| 721 | Are the limitations discussed? | Pai (2020) |
| 722 | Are there data in the results section, which are not referred to in the discussion? | Pai (2020) |
| 723 | Are sources of potential bias discussed? | Lapin (2020) |
| 724 | Begin with summary of hypothesis and aims and primary and secondary findings | Brown et al (2017) |
| 725 | Compare and contrast with prior research | Brown et al (2017) |
| 726 | Relevance and context of findings - external generalizability | Brown et al (2017) |
| 727 | Strengths and limitations | Brown et al (2017) |
| 728 | Future directions | Brown et al (2017) |
| Conclusion | | |
| 729 | Conclusions clear, sound & supported by data | Alexander (2005) |
| 730 | Is the conclusion succinct? | Allen (2014) |
| 731 | Conclusion: - Do the data justify the conclusions? | Allen (2014) |
| 732 | Are the conclusions valid? | Estrada et al. (2006) |
| 733 | Do results justify the conclusion? | Heddle and Ness (2009) |
| 734 | Are the conclusions supported by the data? | Kotsis and Chung (2040) |
| 735 | Are important discussion points missing? | Kotsis and Chung (2041) |
| 736 | Do the authors suggest future research on this topic? | Kotsis and Chung (2042) |
| 737 | Does previous research on this topic support or refute the findings? Do the authors discuss these other studies? | Kotsis and Chung (2043) |
| 738 | Do the authors discuss assumptions, limitations and sources of bias? | Kotsis and Chung (2044) |
| 739 | Have the authors overlooked critical references and/or only selected a biased range of papers? | Kotsis and Chung (2045) |
| 740 | Is new information being presented? | Oerther and Watson (2019) |
| 741 | Provide recommendations for practice, education or research. | Oerther and Watson (2019) |
| 742 | For studies that conclude “no difference” or “no adverse effects,” does the sample size give adequate statistical power to make such a conclusion credible? | Rosenfeld (2010) |
| 743 | Interpretation and conclusions: Warranted by and sufficiently derived from/focused on the data? Message clear? | Rostami et al. (2011) |
| 744 | Are the key conclusions adequately supported by the experimental data? | Seals and Tanaka (2000) |
| 745 | In a conclusion statement (at end of Discussion unless journal has a separate, Conclusion section), does author generalize results within boundaries of the outcomes reported and subject sample? | Simpson (2008) |
| 746 | Conclusion: Do the research findings justify the author’s conclusions? | Smith et al. (2018) |
| 747 | Conclusion: Are the findings consistent with other literature in this area? (if applicable) | Smith et al. (2018) |
| 748 | Conclusion: Is the conclusion effective and succinct? | Smith et al. (2018) |
| 749 | Discussion: Do the conclusions go beyond the results of the study? | Smolčić and Simundić (2014) |
| 750 | Discussion: Is the *Conclusion* is identical to the *Conclusion* part of the *Abstract*? | Smolčić and Simundić (2014) |
| 751 | Are the conclusions supported by the data shown? | Stahel and Moore (2016) |
| 752 | The reported findings should be balanced relative to the context of the stated hypothesis and their scientific value placed into perspective with regards to their clinical or experimental implications | Stahel and Moore (2016) |
| 753 | Discussion, Are the conclusions drawn supported by the results presented? | Sucato and Holland-Hall (2018) |
| 754 | Discussion: (g) Is there a clear and concise conclusion about the implications of the study and next steps, if appropriate? | Tandon (2014) |
| 755 | Discussion: (h) Do the study conclusions clearly flow from the results and are NOT overstated or otherwise inappropriately stated? | Tandon (2014) |
| 756 | Is the treatment efficacious? | Walker (1997) |
| 757 | Is it effective? | Walker (1997) |
| 758 | Is the work technically correct? Are the main conclusions justified by the experimental data and by logically valid arguments? Are the theorems stated and proved correctly given the assumptions? In practical applications of the theoretical results, do the authors check the validity of the underlying assumptions? | Wilson (2002) |
| 759 | Conclusions supported by data? | Lippi (2018) |
| 760 | Conclusions: are they based only on the presented results? | Marušic et al. (2005) |
| 761 | Conclusions Are they justified? | Paice (2001) |
| 762 | Address conclusion | Brown et al (2017) |
| 763 | Do the results support the conclusions? | Pai (2020) |
| References | | |
| 764 | Appropriateness/accuracy of references | Alexander (2005) |
| 765 | Presentation—“Is it enjoyable and easy to read?”: d. Review references. Are they carefully selected and appropriate? A quick spot check for important and recent work is helpful. References should neither be too many nor too few. | Allen and Ho (2017) |
| 766 | References: - Is recent and pertinent scientific literature cited? | Allen (2014) |
| 767 | References: - Are original (not secondary) sources used? | Allen (2014) |
| 768 | References included in accordance with journal style - 0 + | Duchesne (2008) |
| 769 | Is the literature review current and does it place the study in appropriate context? | Estrada et al. (2006) |
| 770 | Are key papers quoted? | Heddle and Ness (2009) |
| 771 | Does the discussion: Include current literature? | Heddle and Ness (2009) |
| 772 | Are the references carefully selected and appropriate? Now, most reviewers will not take the time to double-check most of the references, but a quick, spot check is important, as this will sometimes point to flaws. | Hill (2023) |
| 773 | Provide exhaustive comments: Reference list fulfils journal’s guidelines, is appropriate and does not include many self-citations | Lippi (2018) |
| 774 | Suitable reference list? | Lippi (2018) |
| 775 | References: are they accurate and up-to-date? | Marušic et al. (2005) |
| 776 | References: are they written according to guidelines for authors? | Marušic et al. (2005) |
| 777 | References: are there any obvious mistakes? | Marušic et al. (2005) |
| 778 | References Are they accurate? Are important references missing? | Paice (2001) |
| 779 | Does the reference list follow the format for the journal? | Provenzale and Stanley (2005) |
| 780 | Does the reference list contain errors? | Provenzale and Stanley (2005) |
| 781 | Have the authors appropriately represented the salient points in the articles in the reference list? Alternatively, have the authors misquoted the references? | Provenzale and Stanley (2005) |
| 782 | Are there important references that are not mentioned that should be noted? | Provenzale and Stanley (2005) |
| 783 | Are there more references than are necessary? | Provenzale and Stanley (2005) |
| 784 | References: Up to date and relevant? Any glaring omissions? | Rostami et al. (2011) |
| 785 | Citations are provided when providing evidence-based information from outside sources. | Simpson (2008) |
| 786 | If manuscript likely will be accepted with only minor changes, check that the reference style is correct and consistently applied in text and in reference list. | Simpson (2008) |
| 787 | Check quickly to see if references used are likely up-to-date. | Simpson (2008) |
| 788 | References: Are references cited in an appropriate and accurate format? (will be specific to the target journal) | Smith et al. (2018) |
| 789 | References: Is the number of references appropriate for the type of research and length of the manuscript? | Smith et al. (2018) |
| 790 | References: Are the references up-to-date? | Smolčić and Simundić (2014) |
| 791 | References: Are the references formatted according to journal style? | Smolčić and Simundić (2014) |
| 792 | References: Are references numbered consecutively in the manuscript? | Smolčić and Simundić (2014) |
| 793 | Relevance, timeliness, and comprehensiveness of the cited bibliography; as a rule of thumb, about 80 % of all cited references should be representative of the peer reviewed literature from the preceding 3–5 years | Stahel and Moore (2016) |
| 794 | Introduction, Is the most relevant and up-to-date background literature cited? | Sucato and Holland-Hall (2018) |
| 795 | Are alternative sources of information available, and do they provide substantial information? | Sylvia and Herbel (2001) |
| 796 | If a book has been cited as reference, the format should be ISBN: XXXX, where XXXX is the respective ISBN number. | Talanow (2014) |
| 797 | References: (a) Does the reference list follow the Journal format? | Tandon (2014) |
| 798 | References: (b) Does the reference list contain errors? | Tandon (2014) |
| 799 | References: (c) Are important relevant references all included? Are there major omissions? | Tandon (2014) |
| 800 | References: (d) Are salient points of cited articles accurately quoted? | Tandon (2014) |
| 801 | References: (e) Are there more references than necessary? | Tandon (2014) |
| 802 | Does the manuscript contain an adequate set of references? Is adequate credit given to prior work in the field upon which the present paper is built? | Wilson (2002) |
| 803 | Vancouver style or as per journal instructions/requirement. | Tullu and Karande (2020) |
| 804 | Whether most of the references are recent (from the past 5-10 years). | Tullu and Karande (2020) |
| 805 | Whether the number of references is within the maximum limit prescribed by the journal. | Tullu and Karande (2020) |
| 806 | Whether the references are cited correctly in the text. | Tullu and Karande (2020) |
| 807 | Check the references for misinterpreted or misquoted paper. | Pai (2020) |
| 808 | Are the references up to date? | Pai (2020) |
| 809 | Are there too many references? | Pai (2020) |
| 810 | Do make sure that all the relevant papers have been quoted. | Pai (2020) |
| Statistics | | |
| 811 | Meta-Analytic Methods: Is the statistical model clearly described? | Ades et al. (2013) |
| 812 | Heterogeneity in the Relative Treatment Effects: Has the statistical heterogeneity between baseline arms been discussed? | Ades et al. (2013) |
| 813 | Clarity/validity of statistical methods | Alexander (2005) |
| 814 | Statistics: Are the statistical methods appropriate to the study? | Allen (2014) |
| 815 | Statistics reported with name of test, degrees of freedom, and exact P value - 0 + | Duchesne (2008) |
| 816 | Methods - Methods: Statistical test(s) | Duchesne (2008) |
| 817 | Results: Result of statistical test(s) - 0 + - 0 + - 0 + | Duchesne (2008) |
| 818 | Are comparisons with human performance reported with confidence intervals or p values? | England and Cheng (2019) |
| 819 | Statistical methodology Is this adequately described, appropriate? | Paice (2001) |
| 820 | Statistical analysis: is the test suitable, presentation appropriate, and interpretation correct? | Marušic et al. (2005) |
| 821 | Are the methods for statistical analysis described and referenced? | Rosenfeld (2010) |
| 822 | Descriptive statistics Are small samples or skewed data (e.g., follow-up time) described with median and interquartile range instead of mean and standard deviation? | Rosenfeld (2010) |
| 823 | Statistical methods: Statistical methods used to compare groups for primary outcome | Rostami et al. (2011) |
| 824 | Methods: Are the statistical techniques used appropriate for the experimental design? | Seals and Tanaka (2000) |
| 825 | Methods: Are any critical assumptions of the statistical techniques (e.g., independence, homogeneity, normality) violated? | Seals and Tanaka (2000) |
| 826 | Methods: Are the alpha-levels (or the significance level) used to determine statistical significance clearly stated? | Seals and Tanaka (2000) |
| 827 | Where necessary, are standard deviations or standard errors reported for each variable? Is there excessive variability in one or more of the measurements for a particular condition compared with the others? | Seals and Tanaka (2000) |
| 828 | Were appropriate statistical tests chosen? How does the reader know if the assumptions for using parametric tests were met? Posthoc tests appropriate? How was effect size calculated? | Simpson (2008) |
| 829 | Is there enough information to evaluate the statistics, e.g., the F- value is reported, along w/df? You also need to decide if some sort of other information regarding power or effect size is appropriate to request if not reported. | Simpson (2008) |
| 830 | Is the statistical analysis sound and appropriate for study methodology | Smith et al. (2018) |
| 831 | Statistical analysis: Have all data sets been tested for normality, ad is the name of the normality test provided? | Smolčić and Simundić (2014) |
| 832 | Statistical analysis: Do the authors list all the tests used? | Smolčić and Simundić (2014) |
| 833 | Statistical analysis: Do the authors explain their rationale for using different tests? | Smolčić and Simundić (2014) |
| 834 | Statistical analysis: Is the level of statistical significance provided? | Smolčić and Simundić (2014) |
| 835 | Statistical analysis: Are the name, version and manufacturer of statistical programs provided? | Smolčić and Simundić (2014) |
| 836 | Results: Is the statistical analysis appropriate? Are the correct statistical tests used? | Smolčić and Simundić (2014) |
| 837 | Results: Are summary data provided as mean } SD for normal distributions (if ≥30 subjects)? | Smolčić and Simundić (2014) |
| 838 | Results: Are summary data provided as median (Q1-Q3) for non-normal distributions (if <30 subjects)? | Smolčić and Simundić (2014) |
| 839 | Results: Is age provided as median (min-max)? | Smolčić and Simundić (2014) |
| 840 | Soundness of statistical analysis; consider a recommendation to request an additional review by a qualified biostatistician; values of measured variables must be shown with error limits (standard deviation) and statistical significance | Stahel and Moore (2016) |
| 841 | Are the statistical analyses correct? | Pai (2020) |
| Tables/Figures | | |
| 842 | Presentation of the Data: Is there a clear table or diagram showing which data have been included in the base-case analysis? | Ades et al. (2013) |
| 843 | Presentation of the Data: Is there a clear table or diagram showing which data have been excluded and why? | Ades et al. (2013) |
| 844 | Figures and tables: - Is the information in the tables and figures easy to interpret? Should they be simplified or expanded? | Allen (2014) |
| 845 | Figures and tables: - Are the tables and figures detailed enough to stand on their own without reference to the text? | Allen (2014) |
| 846 | Figures and tables: - Does information in the tables and figures match the information in the text, particularly data? | Allen (2014) |
| 847 | Figures: Are they justified? Are they sharp, with lettering proportionate to the size of the figure? Are there legends to explain the figures? | Benos et al. (2003) |
| 848 | Tables: Can they be simplified or condensed? Should any be omitted? | Benos et al. (2003) |
| 849 | Tables: 1. Do the titles briefly describe the data or the question or purpose? | Brand (2012) |
| 850 | Tables: 2. Are the data self-explanatory without reference to the text? | Brand (2012) |
| 851 | Tables: 3. Are data in the table consistent with those in the text? Do the data supplement and complement, or merely repeat data in the text? | Brand (2012) |
| 852 | Tables: 4. Are the tables clearly numbered? | Brand (2012) |
| 853 | Figures: 1. Are all figures and illustrations necessary to address the key questions or purposes? | Brand (2012) |
| 854 | Figures: 2. Are the figures clear and of publishable quality? | Brand (2012) |
| 855 | Figures: 3. Do the legends adequately describe the figures and in the case of data figures or tables, do they include the key point? | Brand (2012) |
| 856 | Figures: 4. Are the figures clearly numbered? | Brand (2012) |
| 857 | Reporting the findings _ Do the use of tables and figures summarize the data and make the data more understandable? | Christenbery (2011) |
| 858 | Appropriate figures of sufficient quality with proper footnotes - 0 + | Duchesne (2008) |
| 859 | Appropriate tables - 0 + | Duchesne (2008) |
| 860 | Do the authors provide graphics that show the algorithm is detecting the relevant regions of the images and not overfitting to unrelated features? | England and Cheng (2019) |
| 861 | Are the tables and figures clear? Is there good use of space in the tables? | Estrada et al. (2006) |
| 862 | Do figures correspond with text? Are they clear and necessary? | Heddle and Ness (2009) |
| 863 | Are the figures clear and clean, with appropriate labeling? Do the figure legends stand alone, illuminating and clarifying each figure? Could someone understand the entire study simply by perusing the figures and legends? | Hill (2021) |
| 864 | Do tables, if present, provide added value? | Hill (2022) |
| 865 | Are the figures and graphs appropriate and are they appropriately labeled? Would a different figure better illustrate the findings? | Provenzale and Stanley (2005) |
| 866 | Do the figures and graphs adequately show the important results? | Provenzale and Stanley (2005) |
| 867 | Do arrows need to be added to depict important or subtle findings? | Provenzale and Stanley (2005) |
| 868 | Do the figure legends provide a clear explanation that allows the figures and graphs to be understood without referring to the remainder of the manuscript? | Provenzale and Stanley (2005) |
| 869 | If there are tables, do they appropriately describe the results? Should one or more tables be added? | Provenzale and Stanley (2005) |
| 870 | Appropriateness of illustrations and tables | Schuttpelz-Brauns et al. (2010) |
| 871 | Have tables, figures, and text (the 3 tools used to present data) been used effectively? | Seals and Tanaka (2000) |
| 872 | Are all the figures and tables needed? | Seals and Tanaka (2000) |
| 873 | Are the tables and figures properly labeled with the correct units? | Seals and Tanaka (2000) |
| 874 | Is the scaling of the figures appropriate and unbiased? | Seals and Tanaka (2000) |
| 875 | Are the labels on both axes sufficiently large to be readable after the reduction in size for publication? | Seals and Tanaka (2000) |
| 876 | Are any data presented more than once in the same form (e.g., absolute unit values for glucose uptake are shown in the text or a table and also in a figure)? | Seals and Tanaka (2000) |
| 877 | Have the data been presented in the best manner, e.g., a good graph used rather than a hundred tedious tables? | Simpson (2008) |
| 878 | Are the table titles and/or figure captions clear and identify what is depicted in the table or figure? Abbreviations from the text should only be used in figures or tables if defined in the figure caption or table title (unless the abbreviations are fairly obvious or common). | Simpson (2008) |
| 879 | Check quickly to make sure that they are ‘readable’ and follow principles of good presentation of data in tables and/or figures, e.g., figures are labeled correctly, and are readable. | Simpson (2008) |
| 880 | Are the tables/figures used visually appealing? | Simpson (2008) |
| 881 | Some journals have limits on the number of tables/figures. | Simpson (2008) |
| 882 | Are titles of figures and tables appropriate for the information presented? | Smith et al. (2018) |
| 883 | Do footnotes accurately and completely orient the reader to the information conveyed? | Smith et al. (2018) |
| 884 | Do the X and Y axes of graphs accurate reflect the nature of the data? | Smith et al. (2018) |
| 885 | Figures and tables: Are figures and tables presented necessary and additive to the overall message of the manuscript? | Smith et al. (2018) |
| 886 | Figures and tables: Are any integral figures or tables missing? | Smith et al. (2018) |
| 887 | Results: Do the authors repeat their results in tables and in the text? | Smolčić and Simundić (2014) |
| 888 | Results: Are the tables informative? Are column and row titles logical and informative? | Smolčić and Simundić (2014) |
| 889 | Number and quality of figures, tables, and illustrations | Stahel and Moore (2016) |
| 890 | Are figures and tables clearly labeled and readable? Do they conform to the journal's guidelines? | Sylvia and Herbel (2001) |
| 891 | Does the information they provide add to the overall content of the manuscript or is it redunant? Are there too many tables or figures, and can some of the content be consolidated? If the author dose not have figures or tables, would either be of value? | Sylvia and Herbel (2001) |
| 892 | Does he graphic best represent the data or would another representation be better? | Sylvia and Herbel (2001) |
| 893 | Figure legends and stack legends need the following format: Age, gender and diagnosis in one sentence and Findings and Technique, each separated by a line break and preceded with "Findings" or "Technique". | Talanow (2014) |
| 894 | ALL figures need annotations (e.g. arrows, asterisk) and these need to be explained in the respective figure legend. | Talanow (2014) |
| 895 | Acceptable presentation (including tables and figures)? | Lippi (2018) |
| 896 | Clarity of tables & figures | Alexander (2005) |
| 897 | Tables and figures Are they helpful, accurate, clear, properly labelled? | Paice (2001) |
| 898 | Results, tables, and figures: (c) Are the tables, figures, and graphs appropriate and adequate? | Tandon (2014) |
| 899 | Results, tables, and figures: (d) Are the tables and figures appropriately labeled or titled and do they meaningfully add to the text? | Tandon (2014) |
| 900 | Summary tables | Hunter (2020) |
| 901 | Are the images and legends relevant? | Pai (2020) |
| 902 | Are the tables really needed? | Pai (2020) |
| 903 | Are these too many or too few? | Pai (2020) |
| 904 | Are the tables and figures appropriate and clearly labelled? | Pai (2020) |
| 905 | Tables and Figures present results in a clear, concise manner | Brown et al (2017) |
| 906 | Clearly labeled | Brown et al (2017) |
| 907 | Do not reiterate results in the text | Brown et al (2017) |
| Ethical Concerns | | |
| 908 | Research violations_ Are there violations of the Guiding Principles in the Care and Use of Laboratory Animals? | Benos et al. (2003) |
| 909 | Research violations_ If the research involved human subjects, were the studies performed in accordance with the Declaration of Helsinki? | Benos et al. (2003) |
| 910 | Research violations_If you have concerns about the welfare of animal or human subjects used by the authors, include written comments to the editor. | Benos et al. (2003) |
| 911 | Privileged document_ This manuscript is a privileged communication; the data and findings are the exclusive property of the author(s) and should not be disclosed to others who might use this information in their research. | Benos et al. (2003) |
| 912 | Privileged document_ The manuscript, illustrations, and tables should be destroyed upon completing the review or, if anticipating a revision, kept confidential until the review process is complete. | Benos et al. (2003) |
| 913 | Privileged document_ If you have shared responsibility for the review of this manuscript with a colleague, please provide that person’s name and institutional affiliation. | Benos et al. (2003) |
| 914 | Acknowledgements: 1. Do the authors fully disclose sources of financial support and/or other sources of potential conflict of interest or bias? | Brand (2012) |
| 915 | Acknowledgements: 2. Do the authors acknowledge individuals who might have contributed to the manuscript but might not meet criteria for authorship; and do the authors include every individual who would meet criteria for authorship? | Brand (2012) |
| 916 | Ethical considerations _ Is there discussion regarding the rights of the study’s participants? | Christenbery (2011) |
| 917 | Ethical consideration _ Is there evidence that the rights of participants have been protected? | Christenbery (2011) |
| 918 | No evidence of plagiarism | Duchesne (2008) |
| 919 | Ethics: Social, safety, ethical or economic issues | Duchesne (2008) |
| 920 | Ethics: Ethics committee approval and informed consent for subjects | Duchesne (2008) |
| 921 | Ethics: Human study - in accordance with the Declaration of Helsinki | Duchesne (2008) |
| 922 | Ethics: Animal study - in accordance with the Guiding Principles - | Duchesne (2008) |
| 923 | Ethics: Sources of funding and role of study sponsor(s) - | Duchesne (2008) |
| 924 | Ethics: Contributorship, guarantorship, medical writer involvement - | Duchesne (2008) |
| 925 | Ethics: Potential conflicts of interest | Duchesne (2008) |
| 926 | Are there any ethical issues that need to be addressed? | Estrada et al. (2006) |
| 927 | Was the study conducted ethically and with Institutional Review Board approval, if necessary? | Kotsis and Chung (2045) |
| 928 | Are there any potential biases in reviewing this manuscript? | Provenzale and Stanley (2005) |
| 929 | Has the manuscript been previously published? | Provenzale and Stanley (2005) |
| 930 | Authorship Did all authors contribute substantially to the research, draft, or revision of the manuscript, and approve the final version? | Rosenfeld (2010) |
| 931 | Are any ghostwriters or hidden authors suspected based on the tone and style? | Rosenfeld (2010) |
| 932 | Are there signs of plagiarism? | Rosenfeld (2010) |
| 933 | Is this an incremental manuscript that adds marginally to already published data (e.g., new subjects, outcomes, time points) without acknowledging the relationship? | Rosenfeld (2010) |
| 934 | Is the manuscript simply a translation of published work in another language? | Rosenfeld (2010) |
| 935 | If a review article, has it been submitted to more than one journal? | Rosenfeld (2010) |
| 936 | Research subjects Was the research approved, or explicitly exempted from approval, by an ethics panel or institutional review board? | Rosenfeld (2010) |
| 937 | Was informed consent obtained and documented, if appropriate? | Rosenfeld (2010) |
| 938 | Has consent been obtained to use identifiable images or photographs? | Rosenfeld (2010) |
| 939 | Conflict of interest Do the content or conclusions of the manuscript appear to be biased because of a relevant conflict of interest (even if fully disclosed by the authors)? | Rosenfeld (2010) |
| 940 | For sponsored research, did the funding source influence access to data, writing of the manuscript (e.g., employees as authors), or the decision to publish? | Rosenfeld (2010) |
| 941 | Are any undisclosed conflicts of interest suspected? | Rosenfeld (2010) |
| 942 | Is the manuscript original, approved by an institutional review board (if applicable), and unbiased with regards to conflicts of interest? | Rosenfeld (2010) |
| 943 | Methods: Are ethical issues such as informed consent and institutional review board approval described? | Seals and Tanaka (2000) |
| 944 | Is there a significant conflict of financial or scientific interest? | Seals and Tanaka (2000) |
| 945 | If human participants were involved, is there a statement that informed consent was obtained and that the study was approved by the institution’s human subject review board? | Simpson (2008) |
| 946 | Did study participants sign informed consent? | Smolčić and Simundić (2014) |
| 947 | Was the study approved by the relevant institutional ethical committee? | Smolčić and Simundić (2014) |
| 948 | Any additional perceived concern that requires clarification, such as a potential conflict of interest by the authors (e.g. by apparent promotion of a specific surgical product instead of a surgical technique, use of company trade names instead of generic product designations, etc.), should be addressed | Stahel and Moore (2016) |
| 949 | The methods section must provide a statement on study approval by the institution’s ethical review board (for clinical study) or by the animal care committee (for experimental studies), as appropriate | Stahel and Moore (2016) |
| 950 | Was the study approved by the institution's human subject committee, or other appropriate regulatory body? | Sucato and Holland-Hall (2018) |
| 951 | Has been any false or exaggerated information provided in the manuscript? If yes, which one? Mention this in the comment section. | Talanow (2014) |
| 952 | Is there a statement of Institutional Review Board review, approval and the informed consent process? | Tandon (2014) |
| 953 | Overall: (d) Ethical concerns, if any. | Tandon (2014) |
| 954 | Authors’ list reflects individual contribution | Lippi (2018) |
| 955 | Is there evidence of image manipulation? | Pai (2020) |
| 956 | Is there plagiarism of the text or images? | Pai (2020) |
| 957 | Are there any ethical issues? Has ethics committee consent been taken? | Pai (2020) |
| Significance/Relevance | | |
| 958 | Overall: (a) Importance of the central question (what important gap in the existing literature does the paper seek to fill?) | Tandon (2014) |
| 959 | Has been such a topic already published in the JRCR? | Talanow (2014) |
| 960 | Importance of topic to field | Alexander (2005) |
| 961 | Relevance to practice | Alexander (2005) |
| 962 | Relevance to science | Alexander (2005) |
| 963 | Significance of findings | Alexandrov et al. (2009) |
| 964 | Interest to readership | Alexandrov et al. (2009) |
| 965 | Significance—“Does anybody give a #*&%?”: a. State whether you find the study interesting or not. Does it have a meaningful hypothesis and rationale, with relevant implications? | Allen and Ho (2017) |
| 966 | Significance and originality _ Does the manuscript convey an important topic (i.e., frequency and severity of the problem, how will patients benefit)? | Christenbery (2011) |
| 967 | Significance and originality _ Does the research problem statement identify the critical issues and present a context for the study? | Christenbery (2011) |
| 968 | Significance and originality _ To answer these questions, has the author presented a concise yet critical review of the literature? | Christenbery (2011) |
| 969 | Relevance to advanced practice nursing _ Is it clear that the study adds to the APN’s body of knowledge? | Christenbery (2011) |
| 970 | Relevance to advanced practice nursing _ Can the findings be generalized beyond the study sample? If so, to what groups? | Christenbery (2011) |
| 971 | Relevance to advanced practice nursing _ Is transferability of findings to other settings disused? | Christenbery (2011) |
| 972 | Relevance to advanced practice nursing _ Are the relevance of the study’s findings discussed in relation to advanced practice nursing? | Christenbery (2011) |
| 973 | Manuscript appeal to journal readers | Duchesne (2008) |
| 974 | Innovation and impact: Original concepts, new technology usage | Duchesne (2008) |
| 975 | Innovation and impact: Scientific and/or practical value of findings and results | Duchesne (2008) |
| 976 | Is the paper appropriate for the JGIM audience? | Estrada et al. (2006) |
| 977 | What are the relevance and implications of the findings? | Estrada et al. (2006) |
| 978 | Is the research question relevant to the readers of the journal? | Kotsis and Chung (2045) |
| 979 | Does the study answer a question or contribute to the field? | Kotsis and Chung (2045) |
| 980 | Fit for the journal? | Lippi (2018) |
| 981 | Practical significance? | Lippi (2018) |
| 982 | Does the author's research contribute to any knowledge that is useful to the nursing discipline? | Oerther and Watson (2019) |
| 983 | Relevance Is it of interest to the journal’s readership? | Paice (2001) |
| 984 | Does the manuscript address an important problem? | Provenzale and Stanley (2005) |
| 985 | Is the information in this manuscript consistent with the journal’s mission and relevant to the readership? | Rosenfeld (2010) |
| 986 | Does this manuscript significantly improve the knowledge base beyond what is already published on this topic? | Rosenfeld (2010) |
| 987 | Overall evaluation of article (Current, original, relevance of article for reader; contribution to developing research area) | Schuttpelz-Brauns et al. (2010) |
| 988 | Introduction: Is the functional, biological, and/or clinical significance of the topic established? | Seals and Tanaka (2000) |
| 989 | Is the significance of the present results described? Is it clear how the findings extend previous knowledge in a meaningful way? | Seals and Tanaka (2000) |
| 990 | Introduction: Does the study have novelty? | Smolčić and Simundić (2014) |
| 991 | What is the overall significance of the study? | Stahel and Moore (2016) |
| 992 | Overall novelty and innovative aspects of the research question | Stahel and Moore (2016) |
| 993 | Overall value and relevance of the study (‘So what?’ question) | Stahel and Moore (2016) |
| 994 | Potential Impact of the Research: Do you believe the project adds to the literature in a meaningful way? | Stone et al. (2018) |
| 995 | Potential Impact of the Research: Are the data and results presented different from those already available in the literature? Will the readers’ time be well spent reviewing the work? | Stone et al. (2018) |
| 996 | General, Is the subject matter of interest to the journal's readers? | Sucato and Holland-Hall (2018) |
| 997 | How relevant or important is this paper? | Sylvia and Herbel (2001) |
| 998 | How complete is the author’s literature search of the topic? In particular, it is important to determine whether a related manuscript recently was published in the same journal. If so, did the author fail to address it? | Sylvia and Herbel (2001) |
| 999 | The level of interest and recommendation for publication urgency | Talanow (2014) |
| 1000 | Are the problems discussed in the paper of substantial interest? Would solutions of these problems materially advance knowledge of theory, methods, or applications? | Wilson (2002) |
| 1001 | Whether the paper contributes something new to the existing knowledge (novelty). | Tullu and Karande (2020) |
| 1002 | Will the paper be of interest to readers of the journal? | Pai (2020) |
| 1003 | Is there a new message? | Pai (2020) |
| 1004 | Will the readers be interested in the paper? Will the paper stimulate new thinking? Or change practice? | Pai (2020) |
| Originality | | |
| 1005 | Originality of topic/data/methods | Alexander (2005) |
| 1006 | Originality | Alexandrov et al. (2009) |
| 1007 | Novelty—“Is it new?”: a. Assess whether the study questions and findings are novel or not. Lack of originality is the most common reason for rejection in higher-tier journals. A well-conducted study that confirms prior work is often relegated to lower-tier journals. | Allen and Ho (2017) |
| 1008 | Novelty—“Is it new?”: b. Conduct a brief literature search of recent publications. A search for similar publications by the same authors may identify duplication and modification of previously published work. | Allen and Ho (2017) |
| 1009 | Significance and originality of the research question or problem _ Has the problem previously been addressed? | Christenbery (2011) |
| 1010 | Significance and originality _ Have prior studies failed to sufficiently address this problem? | Christenbery (2011) |
| 1011 | Is the paper a useful original contribution? | Estrada et al. (2006) |
| 1012 | Is the research question original? | Kotsis and Chung (2045) |
| 1013 | Originality (Plagiarism): Check abstract and methods through appropriate and free search engines. This check can never be adequate. Reviewers need to be well oriented in the research field of the manuscripts they accept to review. | Kyrgidis and Triaridis (2010) |
| 1014 | Novelty? | Lippi (2018) |
| 1015 | Originality Does this paper add anything new? Am I being open to a new idea? | Paice (2001) |
| 1016 | Originality Does the manuscript appear to duplicate already published work? | Rosenfeld (2010) |
| 1017 | Introduction: Do the authors list recent relevant studies? | Smolčić and Simundić (2014) |
| 1018 | General, Is the work original? Does it contribute new knowledge to the field? | Sucato and Holland-Hall (2018) |
| 1019 | Overall: (b) Originality of the work. | Tandon (2014) |
| 1020 | Does the author either solve these problems or else make a contribution toward a solution that improves substantially upon previous work? | Wilson (2002) |
| 1021 | Originality to be rated good/fair or adequate/poor. | Tullu and Karande (2020) |
| Reporting | | |
| 1022 | Did the authors follow the Standards for Reporting of Diagnostic Accuracy (STARD)/Consolidated Standards of Reporting Trials (CONSORT)/Preferred Reporting Items for Systematic Systematic Reviews and Meta-Analyses (PRISMA)/Strengthening the Reporting of Observational Studies in Epidemiology (STROBE) Statement? | Kotsis and Chung (2045) |
| 1023 | If an RCT, has CONSORT been followed? (If observational, STROBE) - reporting guidelines | Heddle and Ness (2009) |
| 1024 | Subjects: Do the authors follow the recommended reporting guidelines for their type of study (available at http://www.equator-network.org/)? | Smolčić and Simundić (2014) |
| 1025 | Does reporting adhere to the EQUATOR reporting recommendations for the particular study? | Pai (2020) |
| Presentation | | |
| 1026 | Presentation—“Is it enjoyable and easy to read?”: a. Provide a general impression of the writing and presentation. Do not attempt to rewrite a manuscript or give a lengthy list of edits. One can say that syntax/grammar should be significantly improved, supplemented by a few examples of errors. Many journals partner with writing consultants who can be suggested to assist with revisions. | Allen and Ho (2017) |
| 1027 | Presentation—“Is it enjoyable and easy to read?”: b. Consider length. A comment such as “This manuscript is too long” is less helpful; provide specific suggestions for eliminating parts, condensing others, and where additional detail would be helpful. | Allen and Ho (2017) |
| 1028 | Neutral & logical presentation of findings | Alexander (2005) |
| 1029 | Overall grammar, writing style & presentation | Alexander (2005) |
| 1030 | Style and clarity of presentation | Alexandrov et al. (2009) |
| 1031 | Well written? | Lippi (2018) |
| 1032 | Are subject headings optimal terms for the topic? (in terms of reviewing a systematic overview, meta-analysis, or evidence-based review). | Sylvia and Herbel (2001) |
| 1033 | Does the paper have a logical flow and reveal consistency of thought? | Sylvia and Herbel (2001) |
| 1034 | Is it well organized? | Sylvia and Herbel (2001) |
| 1035 | Presentations_ Writing: Is it clear, concise, and in good English? | Benos et al. (2003) |
| 1036 | Trade names, abbreviations, symbols: Are these misused? | Benos et al. (2003) |
| 1037 | Different manuscript sections of appropriate length | Duchesne (2008) |
| 1038 | Clarity of writing | Duchesne (2008) |
| 1039 | Spelling and grammar level | Duchesne (2008) |
| 1040 | Metric system units | Duchesne (2008) |
| 1041 | Error-free formulas and derivations | Duchesne (2008) |
| 1042 | Title page with relevant information | Duchesne (2008) |
| 1043 | Is the writing clear and concise? | Estrada et al. (2006) |
| 1044 | Presentation issues (spelling errors grammatical errors, inaccurate/incomplete references, organizational problems, wording changes) | Heddle and Ness (2009) |
| 1045 | Provide exhaustive comments: The style and language of the article are adequate | Lippi (2018) |
| 1046 | Is there too much detail that would be better suited for an appendix? | Rosenfeld (2010) |
| 1047 | Format - Legibility, style, grammar, orthography, structuring of text, intelligibility | Schuttpelz-Brauns et al. (2010) |
| 1048 | Is the manuscript concise (are there unnecessary sections that should be shortened or liminated)? | Seals and Tanaka (2000) |
| 1049 | Was the paper well written, properly organized, and easy to follow? | Seals and Tanaka (2000) |
| 1050 | Was the information presented in an open-minded and objective manner? | Seals and Tanaka (2000) |
| 1051 | Writing: Is the manuscript easy to follow, that is, has a logical progression and evident organization? | Simpson (2008) |
| 1052 | Is the manuscript concise and understandable? Any parts that should be reduced, eliminated/expanded/added? | Simpson (2008) |
| 1053 | Note if there are major problems with mechanics: grammar, punctuation, spelling. | Simpson (2008) |
| 1054 | Abbreviations: Used judiciously and are composed such that reader won’t have trouble remembering what an abbreviation represents. | Simpson (2008) |
| 1055 | Follows style, format and other rules of the journal. | Simpson (2008) |
| 1056 | Is presentation of the data clear, organized, and specific (e.g. lists patient demographics, treatment compliance, adverse events, etc.)? | Smith et al. (2018) |
| 1057 | Clarity of writing, organization of the paper, spelling and formatting errors, inconsistent or unnecessary use of abbreviations, etc. | Stahel and Moore (2016) |
| 1058 | General, Are there excessive grammatical errors? | Sucato and Holland-Hall (2018) |
| 1059 | General, Is the manuscript clear and well organized? | Sucato and Holland-Hall (2018) |
| 1060 | Overall: (e) Writing style and manuscript flow. | Tandon (2014) |
| 1061 | Are the clarity and readability of the manuscript acceptable? Is the writing grammatically correct? | Wilson (2002) |
| 1062 | Is the material appropriately organized into an effective mix of text, figures and tables? | Wilson (2002) |
| 1063 | Whether the paper gives clear and concise information. | Tullu and Karande (2020) |
| 1064 | Whether the paper follows a logical sequence and avoids repetitions. | Tullu and Karande (2020) |
| 1065 | The writing style is appropriate as per the journal’s instructions to authors. | Tullu and Karande (2020) |
| 1066 | Correct use of English grammar and spellings. | Tullu and Karande (2020) |
| 1067 | Is the paper in the style required by the journal? | Pai (2020) |
| 1068 | Is the English scientific? Are the spellings, grammar and syntax correct? | Pai (2020) |
| Other (Accessibility/Article Category/Data Availability/Reviewer Expertise/Journal Scope/Terminology/Software Availability) | | |
| 1069 | Is the writing accessible to physicians and biomedical researchers? | England and Cheng (2019) |
| 1070 | Use of generic drug names - 0 + | Duchesne (2008) |
| 1071 | Proper use of trade names, abbreviations and symbols - 0 + | Duchesne (2008) |
| 1072 | Manuscript alignment with journal aim and scope - 0 + | Duchesne (2008) |
| 1073 | To which manuscript category does this manuscript best conform? | Provenzale and Stanley (2005) |
| 1074 | Journal: Is the topic appropriate for the journal selected? Would another journal be more appropriate? | Seals and Tanaka (2000) |
| 1075 | Are terms sufficiently defined? | Sylvia and Herbel (2001) |
| 1076 | Are the topic and nature of this paper appropriate for this journal? Are the abstract and introduction accessible to a general reader of this journal? Is the rest of the paper accessible to a readily identified group of readers of this journal? | Wilson (2002) |
| 1077 | Appropriateness for the readership of the journal to be rated good/fair or adequate/poor. | Tullu and Karande (2020) |

**S3B.** List of checklist items from included websites (734 items)

| Title | | |
| --- | --- | --- |
| 1 | Does the title properly reflect the subject of the paper? | Wiley |
| 2 | Whether the title of the article is suitable or effective. | SAGE |
| 3 | Does it express clearly what the manuscript is about? | Taylor and Francis |
| 4 | Does it highlight the importance of the study? | Taylor and Francis |
| 5 | Does it contain any unnecessary description? | Taylor and Francis |
| 6 | The title is clear and informative. | Council of Science Editors |
| 7 | The title is representative of the content and breath of the study (not misleading). | Council of Science Editors |
| 8 | The title captures the importance of the study and the attention of the reader. | Council of Science Editors |
| 9 | Identification as a randomised trial in the title | EQUATOR |
| 10 | Identify the report as a systematic review, meta-analysis, or both. | EQUATOR |
| 11 | Indicate the study’s design with a commonly used term in the title or the abstract | EQUATOR |
| 12 | Descriptive title identifying the study design, population, interventions, and, if applicable, trial acronym | EQUATOR |
| 13 | Identification as a study of diagnostic accuracy using at least one measure of accuracy (such as sensitivity, specificity, predictive values, or AUC) | EQUATOR |
| 14 | The diagnosis or intervention of primary focus followed by the words “case report”. | EQUATOR |
| 15 | Indicate that the manuscript concerns an initiative to improve healthcare (broadly defined to include the quality, safety, effectiveness, patient-centeredness, timeliness, cost, efficiency, and equity of healthcare) | EQUATOR |
| 16 | Identify the study as an economic evaluation or use more specific terms such as “cost-effectiveness analysis”, and describe the interventions compared. | EQUATOR |
| 17 | Concise description of the nature and topic of the study Identifying the study as qualitative or indicating the approach (e.g., ethnography, grounded theory) or data collection methods (e.g., interview, focus group) is recommended | EQUATOR |
| 18 | The title provides a distilled description of the complete article and should include information that, along with the abstract, will make electronic retrieval of the article sensitive and specific. | ICMJE |
| Abstract | | |
| 19 | Does the abstract provide an accessible summary of the paper? | Wiley |
| 20 | Is the abstract clear, accessible? | Nature |
| 21 | Whether the abstract is a good summary of the article. | SAGE |
| 22 | Is it really a summary? | Elsevier |
| 23 | Does it include key findings? | Elsevier |
| 24 | Is it an appropriate length? | Elsevier |
| 25 | Do the authors summarize the main research question and key findings? | PLOS |
| 26 | Effectiveness of the article abstract and introduction | SAGE |
| 27 | Is the abstract an accurate summary of the research and results, without spin? | Hindawi |
| 28 | Is it a short and clear summary of the aims, key methods, important findings and conclusions? | Taylor and Francis |
| 29 | Does it include enough information to stand alone? | Taylor and Francis |
| 30 | Does it contain unnecessary information? | Taylor and Francis |
| 31 | The abstract is complete (thorough); essential details are presented. | Council of Science Editors |
| 32 | The results in the abstract are presented in sufficient and specific detail. | Council of Science Editors |
| 33 | The conclusions in the abstract are justified by the information in the abstract and the text. | Council of Science Editors |
| 34 | There are no inconsistencies in detail between the abstract and the text. | Council of Science Editors |
| 35 | All of the information in the abstract is present in the text. | Council of Science Editors |
| 36 | The abstract overall is congruent with the text; the abstract gives the same impression as the text. | Council of Science Editors |
| 37 | Structured summary of trial design, methods, results, and conclusions | EQUATOR |
| 38 | Provide a structured summary including, as applicable: background; objectives; data sources; study eligibility criteria, participants, and interventions; study appraisal and synthesis methods; results; limitations; conclusions and implications of key findings; systematic review registration number. | EQUATOR |
| 39 | Indicate the study’s design with a commonly used term in the title or the abstract | EQUATOR |
| 40 | Provide in the abstract an informative and balanced summary of what was done and what was found | EQUATOR |
| 41 | Structured summary of study design, methods, results, and conclusions | EQUATOR |
| 42 | Abstract Introduction – What is unique about this case and what does it add to the scientific literature? | EQUATOR |
| 43 | Abstract: The patient’s main concerns and important clinical findings. | EQUATOR |
| 44 | Abstract: The primary diagnoses, interventions, and outcomes. | EQUATOR |
| 45 | Abstract Conclusion – What are one or more “take-away” lessons from this case report? | EQUATOR |
| 46 | Provide adequate information to aid in searching and indexing | EQUATOR |
| 47 | Summarize all key information from various sections of the text using the abstract format of the intended publication or a structured summary such as: background, local problem, methods, interventions, results, conclusions | EQUATOR |
| 48 | Provide an accurate summary of the research objectives, animal species, strain and sex, key methods, principal findings, and study conclusions. | EQUATOR |
| 49 | Provide a structured summary of objectives, perspective, setting, methods (including study design and inputs), results (including base case and uncertainty analyses), and conclusions. | EQUATOR |
| 50 | Summary of key elements of the study using the abstract format of the intended publication; typically includes background, purpose, methods, results, and conclusions | EQUATOR |
| 51 | Original research, systematic reviews, and meta-analyses require structured abstracts. The abstract should provide the context or background for the study and should state the study's purpose, basic procedures (selection of study participants, settings, measurements, analytical methods), main findings (giving specific effect sizes and their statistical and clinical significance, if possible), and principal conclusions. | ICMJE |
| 52 | It should emphasize new and important aspects of the study or observations, note important limitations, and not overinterpret findings. | ICMJE |
| 53 | Clinical trial abstracts should include items that the CONSORT group has identified as essential. | ICMJE |
| 54 | The ICMJE recommends that journals publish the clinical trial registration number at the end of the abstract. | ICMJE |
| 55 | If the data have been deposited in a public repository and/or are being used in a secondary analysis, authors should state at the end of the abstract the unique, persistent data set identifier, repository name and number. | ICMJE |
| Keywords | | |
| 56 | Do the keywords accurately reflect the content? | Wiley |
| 57 | 2 to 5 key words that identify diagnoses or interventions in this case report (including "case report"). | EQUATOR |
| Introduction | | |
| 58 | Is it effective, clear and well organized? | Elsevier |
| 59 | Does it really introduce and put into perspective what follows? | Elsevier |
| 60 | Do the authors identify other literature on the topic and explain how the study relates to this previously published research? | PLOS |
| 61 | What research question(s) do the authors address? Do they make a good argument for why a question is important? | Springer |
| 62 | Are the research questions valid? | Hindawi |
| 63 | Does it clearly summarize the current state of the topic? | Taylor and Francis |
| 64 | Does it address the limitations of current knowledge in this field? | Taylor and Francis |
| 65 | Does it clearly explain why the study was necessary? | Taylor and Francis |
| 66 | Does it clearly define the aim of the study and is this consistent with the rest of the manuscript? | Taylor and Francis |
| 67 | Is the research question clear and appropriate? | Taylor and Francis |
| 68 | Methodology articles: Is there a rationale for why the new method is needed? | Taylor and Francis |
| 69 | Methodology articles: Is the new method compared to existing approaches? | Taylor and Francis |
| 70 | Review articles: Is there any content which has been previously presented in a review? | Taylor and Francis |
| 71 | The introduction builds a logical case and context for the problem statement. | Council of Science Editors |
| 72 | The problem statement is clear and well articulated. | Council of Science Editors |
| 73 | The conceptual framework is explicit and justified. | Council of Science Editors |
| 74 | The research question (research hypothesis where applicable) is clear, concise, and complete. | Council of Science Editors |
| 75 | The literature review is up-to-date. | Council of Science Editors |
| 76 | Include sufficient scientific background to understand the rationale and context for the study, and explain the experimental approach. | EQUATOR |
| 77 | Explain how the animal species and model used address the scientific objectives and, where appropriate, the relevance to human biology. | EQUATOR |
| 78 | Scientific background and explanation of rationale | EQUATOR |
| 79 | Specific objectives or hypotheses | EQUATOR |
| 80 | Describe the rationale for the review in the context of what is already known. | EQUATOR |
| 81 | Provide an explicit statement of questions being addressed with reference to participants, interventions, comparisons, outcomes, and study design (PICOS). | EQUATOR |
| 82 | Explain the scientific background and rationale for the investigation being reported | EQUATOR |
| 83 | State specific objectives, including any prespecified hypotheses | EQUATOR |
| 84 | Description of research question and justification for undertaking the trial, including summary of relevant studies (published and unpublished) examining benefits and harms for each intervention | EQUATOR |
| 85 | Explanation for choice of comparators | EQUATOR |
| 86 | Specific objectives or hypotheses | EQUATOR |
| 87 | Description of trial design including type of trial (eg, parallel group, crossover, factorial, single group), allocation ratio, and framework (eg, superiority, equivalence, noninferiority, exploratory) | EQUATOR |
| 88 | Scientific and clinical background, including the intended use and clinical role of the index test | EQUATOR |
| 89 | Study objectives and hypotheses | EQUATOR |
| 90 | Briefly summarizes why this case is unique and may include medical literature references. | EQUATOR |
| 91 | Nature and significance of the local problem | EQUATOR |
| 92 | Summary of what is currently known about the problem, including relevant previous studies | EQUATOR |
| 93 | Informal or formal frameworks, models, concepts, and/or theories used to explain the problem, any reasons or assumptions that were used to develop the intervention(s), and reasons why the intervention(s) was expected to work | EQUATOR |
| 94 | Purpose of the project and of this report | EQUATOR |
| 95 | The overall objective(s) of the guideline is (are) specifically described | EQUATOR |
| 96 | The health question(s) covered by the guideline is (are) specifically described. | EQUATOR |
| 97 | The population (patients, public, etc.) to whom the guideline is meant to apply is specifically described. | EQUATOR |
| 98 | Clearly describe the research question, research objectives and, where appropriate, specific hypotheses being tested. | EQUATOR |
| 99 | Provide an explicit statement of the broader context for the study. | EQUATOR |
| 100 | Present the study question and its relevance for health policy or practice decisions. | EQUATOR |
| 101 | Description and significance of the problem/phenomenon studied; review of relevant theory and empirical work; problem statement | EQUATOR |
| 102 | Purpose of the study and specific objectives or questions | EQUATOR |
| 103 | Provide a context or background for the study (that is, the nature of the problem and its significance). | ICMJE |
| 104 | State the specific purpose or research objective of, or hypothesis tested by, the study or observation. | ICMJE |
| 105 | Cite only directly pertinent references, and do not include data or conclusions from the work being reported. | ICMJE |
| 106 | Is the research question clear? | Publons |
| Methods | | |
| 107 | What experiments or interventions were used? | PLOS |
| 108 | Are the experiments or interventions appropriate for addressing the research question? | PLOS |
| 109 | Are conditions adequate and the right controls in place? | PLOS |
| 110 | Is there enough data to draw a conclusion? | PLOS |
| 111 | Do the authors address any possible limitations of the research? | PLOS |
| 112 | Was data collected and interpreted accurately? | PLOS |
| 113 | Do the authors follow best practices for reporting? | PLOS |
| 114 | Does the study conform to ethical guidelines? | PLOS |
| 115 | Could another researcher reproduce the study with the same methods? In other words, have the authors provided enough information to validate the study? | PLOS |
| 116 | Are the methods used appropriate? | Wiley |
| 117 | Validity: Does the manuscript have flaws which should prohibit its publication? If so, please provide details. | Nature |
| 118 | Data & methodology: validity of approach, quality of data, quality of presentation. Please note that we expect our reviewers to review all data, including the supplementary information | Nature |
| 119 | Suggested improvements: please list additional experiments or data that could help strengthening the work in a revision. | Nature |
| 120 | What methods do the authors use to answer the question? Are the methods the most current available or is there a newer more powerful method available? Does their overall strategy seem like a good one, or are there major problems with their methods? Are there other experiments that would greatly improve the quality of the manuscript? If so, are they necessary to make the work publishable? Would any different data help confirm the presented results and strengthen the paper? | Springer |
| 121 | The methodology employed during the research. | SAGE |
| 122 | Scientific Soundness: is the study correctly designed and technically sound? Are the analyses performed with the highest technical standards? Are the data robust enough to draw the conclusions? Are the methods, tools, software, and reagents described with sufficient details to allow another researcher to reproduce the results? | MDPI |
| 123 | Can a colleague reproduce the experiments and get the same outcomes? | Elsevier |
| 124 | Did the authors include proper references to previously published methodology? | Elsevier |
| 125 | Is the description of new methodology accurate? | Elsevier |
| 126 | Could or should the authors have included supplementary material? | Elsevier |
| 127 | Suggest additional experiments or analyses | Elsevier |
| 128 | Is the sample size sufficient? | Hindawi |
| 129 | Are the methods and study design appropriate for answering the research question? | Hindawi |
| 130 | Do the experiments have appropriate controls? | Hindawi |
| 131 | Is the reporting of the methods, including any equipment and materials, sufficiently detailed that the research might be reproduced? | Hindawi |
| 132 | Are the study design and methods appropriate for the research question? | Taylor and Francis |
| 133 | Is there enough detail to repeat the experiments? | Taylor and Francis |
| 134 | Is it clear how samples were collected or how participants were recruited? | Taylor and Francis |
| 135 | Is there any potential bias in the sample or in the recruitment of participants? | Taylor and Francis |
| 136 | Are the correct controls/ validation included? | Taylor and Francis |
| 137 | Are any potential confounding factors considered? | Taylor and Francis |
| 138 | Has any randomization been done correctly? | Taylor and Francis |
| 139 | Is the time-frame of the study sufficient to see outcomes? | Taylor and Francis |
| 140 | Is there sufficient power and appropriate statistics? | Taylor and Francis |
| 141 | Do you have any ethical concerns? | Taylor and Francis |
| 142 | Systematic reviews: Are the search terms and inclusion/ exclusion criteria clearly described? | Taylor and Francis |
| 143 | Systematic reviews: Are the search terms and criteria correct to ensure all the relevant articles are included? | Taylor and Francis |
| 144 | Methodology articles: Is the new method clearly described? | Taylor and Francis |
| 145 | Methodology articles: Is it possible to replicate the new method? | Taylor and Francis |
| 146 | The variables being investigated are clearly identified and presented. | Council of Science Editors |
| 147 | The research design is defined and clearly described, and is sufficiently detailed to permit the study to be replicated. | Council of Science Editors |
| 148 | The design is appropriate (optimal) for the research question. | Council of Science Editors |
| 149 | The design has internal validity; potential confounding variables or biases are addressed. | Council of Science Editors |
| 150 | The design has external validity, including subjects, settings, and conditions. | Council of Science Editors |
| 151 | The design allows for unexpected outcomes or events to occur. | Council of Science Editors |
| 152 | The design and conduct of the study are plausible. | Council of Science Editors |
| 153 | The development and content of the instrument are sufficiently described or referenced, and are sufficiently detailed to permit the study to be replicated. | Council of Science Editors |
| 154 | The measurement instrument is appropriate given the study’s variables; the scoring method is clearly defined. | Council of Science Editors |
| 155 | The psychometric properties and procedures are clearly presented and appropriate. | Council of Science Editors |
| 156 | Observers or raters were sufficiently trained. | Council of Science Editors |
| 157 | Data quality control is described and adequate. | Council of Science Editors |
| 158 | The population is defined clearly, both for subjects (participants) and stimulus (intervention), and is sufficiently detailed to permit the study to be replicated. | Council of Science Editors |
| 159 | The sampling procedures are sufficiently described. | Council of Science Editors |
| 160 | Subject samples are appropriate to the research question. | Council of Science Editors |
| 161 | Stimulus samples are appropriate to the research question. | Council of Science Editors |
| 162 | Selection bias is addressed. | Council of Science Editors |
| 163 | Data analysis procedures are sufficiently described, and are sufficiently detailed to permit the study to be replicated. | Council of Science Editors |
| 164 | Data analysis procedures conform to the research design; hypotheses, models, or theory drives the data analyses. | Council of Science Editors |
| 165 | In qualitative research that relies on words instead of numbers, basic requirements of data reliability, validity, trustworthiness, and absence of bias were fulfilled. | Council of Science Editors |
| 166 | The number of analyses is appropriate. | Council of Science Editors |
| 167 | Description of trial design (such as parallel, factorial) including allocation ratio | EQUATOR |
| 168 | Important changes to methods after trial commencement (such as eligibility criteria), with reasons | EQUATOR |
| 169 | Eligibility criteria for participants | EQUATOR |
| 170 | Settings and locations where the data were collected | EQUATOR |
| 171 | The interventions for each group with sufficient details to allow replication, including how and when they were actually administered | EQUATOR |
| 172 | Completely defined pre-specified primary and secondary outcome measures, including how and when they were assessed | EQUATOR |
| 173 | Any changes to trial outcomes after the trial commenced, with reasons | EQUATOR |
| 174 | How sample size was determined | EQUATOR |
| 175 | When applicable, explanation of any interim analyses and stopping guidelines | EQUATOR |
| 176 | Method used to generate the random allocation sequence | EQUATOR |
| 177 | Type of randomisation; details of any restriction (such as blocking and block size) | EQUATOR |
| 178 | Mechanism used to implement the random allocation sequence (such as sequentially numbered containers), describing any steps taken to conceal the sequence until interventions were assigned | EQUATOR |
| 179 | Who generated the random allocation sequence, who enrolled participants, and who assigned participants to interventions | EQUATOR |
| 180 | If done, who was blinded after assignment to interventions (for example, participants, care providers, those assessing outcomes) and how | EQUATOR |
| 181 | If relevant, description of the similarity of interventions | EQUATOR |
| 182 | Statistical methods used to compare groups for primary and secondary outcomes | EQUATOR |
| 183 | Methods for additional analyses, such as subgroup analyses and adjusted analyses | EQUATOR |
| 184 | Indicate if a review protocol exists, if and where it can be accessed (e.g., Web address), and, if available, provide registration information including registration number. | EQUATOR |
| 185 | Specify study characteristics (e.g., PICOS, length of follow-up) and report characteristics (e.g., years considered, language, publication status) used as criteria for eligibility, giving rationale. | EQUATOR |
| 186 | Describe all information sources (e.g., databases with dates of coverage, contact with study authors to identify additional studies) in the search and date last searched. | EQUATOR |
| 187 | Present full electronic search strategy for at least one database, including any limits used, such that it could be repeated. | EQUATOR |
| 188 | State the process for selecting studies (i.e., screening, eligibility, included in systematic review, and, if applicable, included in the meta-analysis). | EQUATOR |
| 189 | Describe method of data extraction from reports (e.g., piloted forms, independently, in duplicate) and any processes for obtaining and confirming data from investigators. | EQUATOR |
| 190 | List and define all variables for which data were sought (e.g., PICOS, funding sources) and any assumptions and simplifications made. | EQUATOR |
| 191 | Describe methods used for assessing risk of bias of individual studies (including specification of whether this was done at the study or outcome level), and how this information is to be used in any data synthesis. | EQUATOR |
| 192 | State the principal summary measures (e.g., risk ratio, difference in means). | EQUATOR |
| 193 | Describe the methods of handling data and combining results of studies, if done, including measures of consistency (e.g., I^2^) for each meta-analysis. | EQUATOR |
| 194 | Specify any assessment of risk of bias that may affect the cumulative evidence (e.g., publication bias, selective reporting within studies). | EQUATOR |
| 195 | Describe methods of additional analyses (e.g., sensitivity or subgroup analyses, meta-regression), if done, indicating which were pre-specified. | EQUATOR |
| 196 | Present key elements of study design early in the paper | EQUATOR |
| 197 | Describe the setting, locations, and relevant dates, including periods of recruitment, exposure, follow-up, and data collection | EQUATOR |
| 198 | (*a*) *Cohort study*—Give the eligibility criteria, and the sources and methods of selection of participants. Describe methods of follow-up | EQUATOR |
| 199 | *Case-control study*—Give the eligibility criteria, and the sources and methods of case ascertainment and control selection. Give the rationale for the choice of cases and controls | EQUATOR |
| 200 | *Cross-sectional study*—Give the eligibility criteria, and the sources and methods of selection of participants | EQUATOR |
| 201 | (*b*) *Cohort study*—For matched studies, give matching criteria and number of exposed and unexposed | EQUATOR |
| 202 | *Case-control study*—For matched studies, give matching criteria and the number of controls per case | EQUATOR |
| 203 | Clearly define all outcomes, exposures, predictors, potential confounders, and effect modifiers. Give diagnostic criteria, if applicable | EQUATOR |
| 204 | For each variable of interest, give sources of data and details of methods of assessment (measurement). Describe comparability of assessment methods if there is more than one group | EQUATOR |
| 205 | Describe any efforts to address potential sources of bias | EQUATOR |
| 206 | Explain how the study size was arrived at | EQUATOR |
| 207 | Explain how quantitative variables were handled in the analyses. If applicable, describe which groupings were chosen and why | EQUATOR |
| 208 | (*a*) Describe all statistical methods, including those used to control for confounding | EQUATOR |
| 209 | (*b*) Describe any methods used to examine subgroups and interactions | EQUATOR |
| 210 | (*c*) Explain how missing data were addressed | EQUATOR |
| 211 | (*d*) *Cohort study*—If applicable, explain how loss to follow-up was addressed | EQUATOR |
| 212 | *Case-control study*—If applicable, explain how matching of cases and controls was addressed | EQUATOR |
| 213 | *Cross-sectional study*—If applicable, describe analytical methods taking account of sampling strategy | EQUATOR |
| 214 | (*e*) Describe any sensitivity analyses | EQUATOR |
| 215 | Description of study settings (eg, community clinic, academic hospital) and list of countries where data will be collected. Reference to where list of study sites can be obtained | EQUATOR |
| 216 | Inclusion and exclusion criteria for participants. If applicable, eligibility criteria for study centres and individuals who will perform the interventions (eg, surgeons, psychotherapists) | EQUATOR |
| 217 | Interventions for each group with sufficient detail to allow replication, including how and when they will be administered | EQUATOR |
| 218 | Criteria for discontinuing or modifying allocated interventions for a given trial participant (eg, drug dose change in response to harms, participant request, or improving/worsening disease) | EQUATOR |
| 219 | Strategies to improve adherence to intervention protocols, and any procedures for monitoring adherence (eg, drug tablet return, laboratory tests) | EQUATOR |
| 220 | Relevant concomitant care and interventions that are permitted or prohibited during the trial | EQUATOR |
| 221 | Primary, secondary, and other outcomes, including the specific measurement variable (eg, systolic blood pressure), analysis metric (eg, change from baseline, final value, time to event), method of aggregation (eg, median, proportion), and time point for each outcome. Explanation of the clinical relevance of chosen efficacy and harm outcomes is strongly recommended | EQUATOR |
| 222 | Time schedule of enrolment, interventions (including any run-ins and washouts), assessments, and visits for participants. A schematic diagram is highly recommended (see Figure) | EQUATOR |
| 223 | Estimated number of participants needed to achieve study objectives and how it was determined, including clinical and statistical assumptions supporting any sample size calculations | EQUATOR |
| 224 | Strategies for achieving adequate participant enrolment to reach target sample size | EQUATOR |
| 225 | Method of generating the allocation sequence (eg, computer-generated random numbers), and list of any factors for stratification. To reduce predictability of a random sequence, details of any planned restriction (eg, blocking) should be provided in a separate document that is unavailable to those who enrol participants or assign interventions | EQUATOR |
| 226 | Mechanism of implementing the allocation sequence (eg, central telephone; sequentially numbered, opaque, sealed envelopes), describing any steps to conceal the sequence until interventions are assigned | EQUATOR |
| 227 | Who will generate the allocation sequence, who will enrol participants, and who will assign participants to interventions | EQUATOR |
| 228 | Who will be blinded after assignment to interventions (eg, trial participants, care providers, outcome assessors, data analysts), and how | EQUATOR |
| 229 | If blinded, circumstances under which unblinding is permissible, and procedure for revealing a participant’s allocated intervention during the trial | EQUATOR |
| 230 | Plans for assessment and collection of outcome, baseline, and other trial data, including any related processes to promote data quality (eg, duplicate measurements, training of assessors) and a description of study instruments (eg, questionnaires, laboratory tests) along with their reliability and validity, if known. Reference to where data collection forms can be found, if not in the protocol | EQUATOR |
| 231 | Plans to promote participant retention and complete follow-up, including list of any outcome data to be collected for participants who discontinue or deviate from intervention protocols | EQUATOR |
| 232 | Plans for data entry, coding, security, and storage, including any related processes to promote data quality (eg, double data entry; range checks for data values). Reference to where details of data management procedures can be found, if not in the protocol | EQUATOR |
| 233 | Statistical methods for analysing primary and secondary outcomes. Reference to where other details of the statistical analysis plan can be found, if not in the protocol | EQUATOR |
| 234 | Methods for any additional analyses (eg, subgroup and adjusted analyses) | EQUATOR |
| 235 | Definition of analysis population relating to protocol non-adherence (eg, as randomised analysis), and any statistical methods to handle missing data (eg, multiple imputation) | EQUATOR |
| 236 | Composition of data monitoring committee (DMC); summary of its role and reporting structure; statement of whether it is independent from the sponsor and competing interests; and reference to where further details about its charter can be found, if not in the protocol. Alternatively, an explanation of why a DMC is not needed | EQUATOR |
| 237 | Description of any interim analyses and stopping guidelines, including who will have access to these interim results and make the final decision to terminate the trial | EQUATOR |
| 238 | Plans for collecting, assessing, reporting, and managing solicited and spontaneously reported adverse events and other unintended effects of trial interventions or trial conduct | EQUATOR |
| 239 | Frequency and procedures for auditing trial conduct, if any, and whether the process will be independent from investigators and the sponsor | EQUATOR |
| 240 | Whether data collection was planned before the index test and reference standard | EQUATOR |
| 241 | were performed (prospective study) or after (retrospective study) | EQUATOR |
| 242 | Eligibility criteria | EQUATOR |
| 243 | On what basis potentially eligible participants were identified | EQUATOR |
| 244 | (such as symptoms, results from previous tests, inclusion in registry) | EQUATOR |
| 245 | Where and when potentially eligible participants were identified (setting, location and dates) | EQUATOR |
| 246 | Whether participants formed a consecutive, random or convenience series | EQUATOR |
| 247 | Index test, in sufficient detail to allow replication | EQUATOR |
| 248 | Reference standard, in sufficient detail to allow replication | EQUATOR |
| 249 | Rationale for choosing the reference standard (if alternatives exist) | EQUATOR |
| 250 | Definition of and rationale for test positivity cut-offs or result categories | EQUATOR |
| 251 | of the index test, distinguishing pre-specified from exploratory | EQUATOR |
| 252 | Definition of and rationale for test positivity cut-offs or result categories | EQUATOR |
| 253 | of the reference standard, distinguishing pre-specified from exploratory | EQUATOR |
| 254 | Whether clinical information and reference standard results were available | EQUATOR |
| 255 | to the performers/readers of the index test | EQUATOR |
| 256 | Whether clinical information and index test results were available | EQUATOR |
| 257 | to the assessors of the reference standard | EQUATOR |
| 258 | Methods for estimating or comparing measures of diagnostic accuracy | EQUATOR |
| 259 | How indeterminate index test or reference standard results were handled | EQUATOR |
| 260 | How missing data on the index test and reference standard were handled | EQUATOR |
| 261 | Any analyses of variability in diagnostic accuracy, distinguishing pre-specified from exploratory | EQUATOR |
| 262 | Intended sample size and how it was determined | EQUATOR |
| 263 | Diagnostic methods (PE, laboratory testing, imaging, surveys) | EQUATOR |
| 264 | Contextual elements considered important at the outset of introducing the intervention(s) | EQUATOR |
| 265 | Description of the intervention(s) in sufficient detail that others could reproduce it | EQUATOR |
| 266 | Specifics of the team involved in the work | EQUATOR |
| 267 | Approach chosen for assessing the impact of the intervention(s) | EQUATOR |
| 268 | Approach used to establish whether the observed outcomes were due to the intervention(s) | EQUATOR |
| 269 | Measures chosen for studying processes and outcomes of the intervention(s), including rationale for choosing them, their operational definitions, and their validity and reliability | EQUATOR |
| 270 | Description of the approach to the ongoing assessment of contextual elements that contributed to the success, failure, efficiency, and cost | EQUATOR |
| 271 | Methods employed for assessing completeness and accuracy of data | EQUATOR |
| 272 | Qualitative and quantitative methods used to draw inferences from the data | EQUATOR |
| 273 | Methods for understanding variation within the data, including the effects of time as a variable | EQUATOR |
| 274 | Systematic methods were used to search for evidence. | EQUATOR |
| 275 | The criteria for selecting the evidence are clearly described. | EQUATOR |
| 276 | The methods for formulating the recommendations are clearly described. | EQUATOR |
| 277 | The guideline has been externally reviewed by experts prior to its publication. | EQUATOR |
| 278 | A procedure for updating the guideline is provided. | EQUATOR |
| 279 | The guideline presents monitoring and/or auditing criteria. | EQUATOR |
| 280 | Provide details of housing and husbandry conditions, including any environmental enrichment. | EQUATOR |
| 281 | Describe any interventions or steps taken in the experimental protocols to reduce pain, suffering and distress. | EQUATOR |
| 282 | Report any expected or unexpected adverse events. | EQUATOR |
| 283 | Describe the humane endpoints established for the study, the signs that were monitored and the frequency of monitoring. If the study did not have humane endpoints, state this. | EQUATOR |
| 284 | For each experiment, provide brief details of study design including: The groups being compared, including control groups. If no control group has been used, the rationale should be stated. | EQUATOR |
| 285 | For each experiment, provide brief details of study design including: The experimental unit (e.g. a single animal, litter, or cage of animals). | EQUATOR |
| 286 | Specify the exact number of experimental units allocated to each group, and the total number in each experiment. Also indicate the total number of animals used. | EQUATOR |
| 287 | Explain how the sample size was decided. Provide details of any a priori sample size calculation, if done. | EQUATOR |
| 288 | Describe any criteria used for including and excluding animals (or experimental units) during the experiment, and data points during the analysis. Specify if these criteria were established a priori. If no criteria were set, state this explicitly. | EQUATOR |
| 289 | For each experimental group, report any animals, experimental units or data points not included in the analysis and explain why. If there were no exclusions, state so. | EQUATOR |
| 290 | For each analysis, report the exact value of n in each experimental group. | EQUATOR |
| 291 | State whether randomisation was used to allocate experimental units to control and treatment groups. If done, provide the method used to generate the randomisation sequence. | EQUATOR |
| 292 | Describe the strategy used to minimise potential confounders such as the order of treatments and measurements, or animal/cage location. If confounders were not controlled, state this explicitly. | EQUATOR |
| 293 | Describe who was aware of the group allocation at the different stages of the experiment (during the allocation, the conduct of the experiment, the outcome assessment, and the data analysis). | EQUATOR |
| 294 | Clearly define all outcome measures assessed (e.g. cell death, molecular markers, or behavioural changes). | EQUATOR |
| 295 | For hypothesis-testing studies, specify the primary outcome measure, i.e. the outcome measure that was used to determine the sample size. | EQUATOR |
| 296 | Provide species-appropriate details of the animals used, including species, strain and substrain, sex, age or developmental stage, and, if relevant, weight. | EQUATOR |
| 297 | Provide further relevant information on the provenance of animals, health/immune status, genetic modification status, genotype, and any previous procedures. | EQUATOR |
| 298 | For each experimental group, including controls, describe the procedures in enough detail to allow others to replicate them, including: What was done, how it was done and what was used. | EQUATOR |
| 299 | For each experimental group, including controls, describe the procedures in enough detail to allow others to replicate them, including: When and how often. | EQUATOR |
| 300 | For each experimental group, including controls, describe the procedures in enough detail to allow others to replicate them, including: Where (including detail of any acclimatisation periods). | EQUATOR |
| 301 | For each experimental group, including controls, describe the procedures in enough detail to allow others to replicate them, including: Why (provide rationale for procedures). | EQUATOR |
| 302 | Describe characteristics of the base case population and subgroups analysed, including why they were chosen. | EQUATOR |
| 303 | State relevant aspects of the system(s) in which the decision(s) need(s) to be made. | EQUATOR |
| 304 | Describe the perspective of the study and relate this to the costs being evaluated. | EQUATOR |
| 305 | Describe the interventions or strategies being compared and state why they were chosen. | EQUATOR |
| 306 | State the time horizon(s) over which costs and consequences are being evaluated and say why appropriate. | EQUATOR |
| 307 | Report the choice of discount rate(s) used for costs and outcomes and say why appropriate. | EQUATOR |
| 308 | Describe what outcomes were used as the measure(s) of benefit in the evaluation and their relevance for the type of analysis performed. | EQUATOR |
| 309 | Single study-based estimates: Describe fully the design features of the single effectiveness study and why the single study was a sufficient source of clinical effectiveness data. | EQUATOR |
| 310 | Synthesis-based estimates: Describe fully the methods used for identification of included studies and synthesis of clinical effectiveness data. | EQUATOR |
| 311 | If applicable, describe the population and methods used to elicit preferences for outcomes. | EQUATOR |
| 312 | Single study-based economic evaluation: Describe approaches used to estimate resource use associated with the alternative interventions. Describe primary or secondary research methods for valuing each resource item in terms of its unit cost. Describe any adjustments made to approximate to opportunity costs. | EQUATOR |
| 313 | Model-based economic evaluation: Describe approaches and data sources used to estimate resource use associated with model health states. Describe primary or secondary research methods for valuing each resource item in terms of its unit cost. Describe any adjustments made to approximate to opportunity costs. | EQUATOR |
| 314 | Report the dates of the estimated resource quantities and unit costs. Describe methods for adjusting estimated unit costs to the year of reported costs if necessary. Describe methods for converting costs into a common currency base and the exchange rate. | EQUATOR |
| 315 | Describe and give reasons for the specific type of decisionanalytical model used. Providing a figure to show model structure is strongly recommended. | EQUATOR |
| 316 | Describe all structural or other assumptions underpinning the decision-analytical model. | EQUATOR |
| 317 | Describe all analytical methods supporting the evaluation. This could include methods for dealing with skewed, missing, or censored data; extrapolation methods; methods for pooling data; approaches to validate or make adjustments (such as half cycle corrections) to a model; and methods for handling population heterogeneity and uncertainty. | EQUATOR |
| 318 | Qualitative approach (e.g., ethnography, grounded theory, case study, phenomenology, narrative research) and guiding theory if appropriate; identifying the research paradigm (e.g., postpositivist, constructivist/interpretivist) is also recommended; rationale | EQUATOR |
| 319 | Researchers’ characteristics that may influence the research, including personal attributes, qualifications/experience, relationship with participants, assumptions, and/or presuppositions; potential or actual interaction between researchers’ characteristics and the research questions, approach, methods, results, and/or transferability | EQUATOR |
| 320 | Setting/site and salient contextual factors; rationale | EQUATOR |
| 321 | How and why research participants, documents, or events were selected; criteria for deciding when no further sampling was necessary (e.g., sampling saturation); rationale | EQUATOR |
| 322 | Documentation of approval by an appropriate ethics review board and participant consent, or explanation for lack thereof; other confidentiality and data security issues | EQUATOR |
| 323 | Types of data collected; details of data collection procedures including (as appropriate) start and stop dates of data collection and analysis, iterative process, triangulation of sources/methods, and modification of procedures in response to evolving study findings; rationale | EQUATOR |
| 324 | Description of instruments (e.g., interview guides, questionnaires) and devices (e.g., audio recorders) used for data collection; if/how the instrument(s) changed over the course of the study | EQUATOR |
| 325 | Number and relevant characteristics of participants, documents, or events included in the study; level of participation (could be reported in results) | EQUATOR |
| 326 | Methods for processing data prior to and during analysis, including transcription, data entry, data management and security, verification of data integrity, data coding, and anonymization/deidentification of excerpts | EQUATOR |
| 327 | Process by which inferences, themes, etc., were identified and developed, including the researchers involved in data analysis; usually references a specific paradigm or approach; rationale | EQUATOR |
| 328 | Techniques to enhance trustworthiness and credibility of data analysis (e.g., member checking, audit trail, triangulation); rationale | EQUATOR |
| 329 | The Methods section should aim to be sufficiently detailed such that others with access to the data would be able to reproduce the results. | ICMJE |
| 330 | The Methods section should include a statement indicating that the research was approved by an independent local, regional or national review body (e.g., ethics committee, institutional review board). | ICMJE |
| 331 | Clearly describe the selection of observational or experimental participants (healthy individuals or patients, including controls), including eligibility and exclusion criteria and a description of the source population. | ICMJE |
| 332 | Ensure correct use of the terms sex (when reporting biological factors) and gender (identity, psychosocial or cultural factors), and, unless inappropriate, report the sex and/or gender of study participants, the sex of animals or cells, and describe the methods used to determine sex and gender. | ICMJE |
| 333 | If the study was done involving an exclusive population, for example in only one sex, authors should justify why, except in obvious cases, (e.g., prostate cancer).” | ICMJE |
| 334 | Authors should define how they determined race or ethnicity and justify their relevance. | ICMJE |
| 335 | Authors should use neutral, precise, and respectful language to describe study participants and avoid the use of terminology that might stigmatize participants. | ICMJE |
| 336 | Specify the study's main and secondary objectives–usually identified as primary and secondary outcomes. | ICMJE |
| 337 | Identify methods, equipment (give the manufacturer's name and address in parentheses), and procedures in sufficient detail to allow others to reproduce the results. | ICMJE |
| 338 | Give references to established methods, including statistical methods; provide references and brief descriptions for methods that have been published but are not well-known; describe new or substantially modified methods, give the reasons for using them, and evaluate their limitations | ICMJE |
| 339 | Identify precisely all drugs and chemicals used, including generic name(s), dose(s), and route(s) of administration. | ICMJE |
| 340 | Identify appropriate scientific names and gene names. | ICMJE |
| 341 | Was the approach appropriate? | Publons |
| 342 | Is the study design, methods and analysis appropriate to the question being studied? | Publons |
| 343 | Are the methods described clearly enough for other researchers to replicate? | Publons |
| Results | | |
| 344 | Do the results support the conclusions? | PLOS |
| 345 | Do the data provide enough evidence for the authors’ conclusions? | PLOS |
| 346 | Are the necessary data points provided? | PLOS |
| 347 | Have the authors provided a sufficient amount of data and information for other researchers to recreate the analyses? | PLOS |
| 348 | Were the results analyzed and interpreted correctly? Does the evidence support the authors’ conclusions? | Springer |
| 349 | Suggest improvements in the way data is shown | Elsevier |
| 350 | Write concisely and precisely which changes you recommend | Elsevier |
| 351 | Make clear the need for changes/updates | Elsevier |
| 352 | Are the results presented clearly and accurately? | Taylor and Francis |
| 353 | Do the results presented match the methods? | Taylor and Francis |
| 354 | Have all the relevant data been included? | Taylor and Francis |
| 355 | Is there any risk of patients or participants being identified? | Taylor and Francis |
| 356 | Is the data described in the text consistent with the data in the figures and tables? | Taylor and Francis |
| 357 | Systematic reviews: If a meta-analysis has been done, were previous studies combined appropriately? | Taylor and Francis |
| 358 | Case reports: Does the diagnosis appear to be correct? | Taylor and Francis |
| 359 | Case reports: Was the treatment reasonable for the diagnosis? | Taylor and Francis |
| 360 | Case reports: Are the treatment and outcomes clearly described? | Taylor and Francis |
| 361 | Review articles: Is it a balanced and unbiased overview of current understanding? | Taylor and Francis |
| 362 | Results are organized in a way that is easy to understand. | Council of Science Editors |
| 363 | Results are presented effectively; the results are contextualized. | Council of Science Editors |
| 364 | The results are complete. | Council of Science Editors |
| 365 | The amount of data presented is sufficient and appropriate. | Council of Science Editors |
| 366 | For each group, the numbers of participants who were randomly assigned, received intended treatment, and were analysed for the primary outcome | EQUATOR |
| 367 | For each group, losses and exclusions after randomisation, together with reasons | EQUATOR |
| 368 | Dates defining the periods of recruitment and follow-up | EQUATOR |
| 369 | Why the trial ended or was stopped | EQUATOR |
| 370 | A table showing baseline demographic and clinical characteristics for each group | EQUATOR |
| 371 | For each group, number of participants (denominator) included in each analysis and whether the analysis was by original assigned groups | EQUATOR |
| 372 | For each primary and secondary outcome, results for each group, and the estimated effect size and its precision (such as 95% confidence interval) | EQUATOR |
| 373 | For binary outcomes, presentation of both absolute and relative effect sizes is recommended | EQUATOR |
| 374 | Results of any other analyses performed, including subgroup analyses and adjusted analyses, distinguishing pre-specified from exploratory | EQUATOR |
| 375 | All important harms or unintended effects in each group | EQUATOR |
| 376 | Give numbers of studies screened, assessed for eligibility, and included in the review, with reasons for exclusions at each stage, ideally with a flow diagram. | EQUATOR |
| 377 | For each study, present characteristics for which data were extracted (e.g., study size, PICOS, follow-up period) and provide the citations. | EQUATOR |
| 378 | Present data on risk of bias of each study and, if available, any outcome level assessment. | EQUATOR |
| 379 | For all outcomes considered (benefits or harms), present, for each study: (a) simple summary data for each intervention group (b) effect estimates and confidence intervals, ideally with a forest plot. | EQUATOR |
| 380 | Present results of each meta-analysis done, including confidence intervals and measures of consistency. | EQUATOR |
| 381 | Present results of any assessment of risk of bias across studies. | EQUATOR |
| 382 | Give results of additional analyses, if done (e.g., sensitivity or subgroup analyses, meta-regression). | EQUATOR |
| 383 | (a) Report numbers of individuals at each stage of study—eg numbers potentially eligible, examined for eligibility, confirmed eligible, included in the study, completing follow-up, and analysed | EQUATOR |
| 384 | (b) Give reasons for non-participation at each stage | EQUATOR |
| 385 | (c) Consider use of a flow diagram | EQUATOR |
| 386 | (a) Give characteristics of study participants (eg demographic, clinical, social) and information on exposures and potential confounders | EQUATOR |
| 387 | (b) Indicate number of participants with missing data for each variable of interest | EQUATOR |
| 388 | (c) *Cohort study*—Summarise follow-up time (eg, average and total amount) | EQUATOR |
| 389 | *Cohort study*—Report numbers of outcome events or summary measures over time | EQUATOR |
| 390 | *Case-control study—*Report numbers in each exposure category, or summary measures of exposure | EQUATOR |
| 391 | *Cross-sectional study—*Report numbers of outcome events or summary measures | EQUATOR |
| 392 | (*a*) Give unadjusted estimates and, if applicable, confounder-adjusted estimates and their precision (eg, 95% confidence interval). Make clear which confounders were adjusted for and why they were included | EQUATOR |
| 393 | (*b*) Report category boundaries when continuous variables were categorized | EQUATOR |
| 394 | (*c*) If relevant, consider translating estimates of relative risk into absolute risk for a meaningful time period | EQUATOR |
| 395 | Report other analyses done—eg analyses of subgroups and interactions, and sensitivity analyses | EQUATOR |
| 396 | Flow of participants, using a diagram | EQUATOR |
| 397 | Baseline demographic and clinical characteristics of participants | EQUATOR |
| 398 | Distribution of severity of disease in those with the target condition | EQUATOR |
| 399 | Distribution of alternative diagnoses in those without the target condition | EQUATOR |
| 400 | Time interval and any clinical interventions between index test and reference standard | EQUATOR |
| 401 | Cross tabulation of the index test results (or their distribution) | EQUATOR |
| 402 | by the results of the reference standard | EQUATOR |
| 403 | Estimates of diagnostic accuracy and their precision (such as 95% confidence intervals) | EQUATOR |
| 404 | Any adverse events from performing the index test or the reference standard | EQUATOR |
| 405 | De-identified patient specific information | EQUATOR |
| 406 | Primary concerns and symptoms of the patient | EQUATOR |
| 407 | Medical, family, and psychosocial history including relevant genetic information | EQUATOR |
| 408 | Relevant past interventions and their outcomes | EQUATOR |
| 409 | Describe significant physical examination (PE) and important clinical findings. | EQUATOR |
| 410 | Historical and current information from this episode of care organized as a timeline (figure or table). | EQUATOR |
| 411 | Diagnostic challenges | EQUATOR |
| 412 | Diagnosis (including other diagnoses considered) | EQUATOR |
| 413 | Prognostic characteristics when applicable | EQUATOR |
| 414 | Types of therapeutic intervention (pharmacologic, surgical, preventive). | EQUATOR |
| 415 | Administration of therapeutic intervention (dosage, strength, duration). | EQUATOR |
| 416 | Changes in therapeutic interventions with explanations. | EQUATOR |
| 417 | Clinician- and patient-assessed outcomes if available. | EQUATOR |
| 418 | Important follow-up diagnostic and other test results. | EQUATOR |
| 419 | Intervention adherence and tolerability. (How was this assessed?) | EQUATOR |
| 420 | Adverse and unanticipated events | EQUATOR |
| 421 | The patient should share their perspective on the treatment(s) they received. | EQUATOR |
| 422 | Initial steps of the intervention(s) and their evolution over time (*e.g.*, time-line diagram, flow chart, or table), including modifications made to the intervention during the project | EQUATOR |
| 423 | Details of the process measures and outcome | EQUATOR |
| 424 | Contextual elements that interacted with the intervention(s) | EQUATOR |
| 425 | Observed associations between outcomes, interventions, and relevant contextual elements | EQUATOR |
| 426 | Unintended consequences such as unexpected benefits, problems, failures, or costs associated with the intervention(s). | EQUATOR |
| 427 | Details about missing data | EQUATOR |
| 428 | The guideline development group includes individuals from all the relevant professional groups. | EQUATOR |
| 429 | The views and preferences of the target population (patients, public, etc.) have been sought. | EQUATOR |
| 430 | The health benefits, side effects, and risks have been considered in formulating the recommendations. | EQUATOR |
| 431 | For each experiement conducted, including independent replications, report: Summary/descriptive statistics for each experimental group, with a measure of variability where applicable (e.g. mean and SD, or median and range). | EQUATOR |
| 432 | For each experiement conducted, including independent replications, report: If applicable, the effect size with a confidence interval. | EQUATOR |
| 433 | Report the values, ranges, references, and, if used, probability distributions for all parameters. Report reasons or sources for distributions used to represent uncertainty where appropriate. Providing a table to show the input values is strongly recommended. | EQUATOR |
| 434 | For each intervention, report mean values for the main categories of estimated costs and outcomes of interest, as well as mean differences between the comparator groups. If applicable, report incremental cost-effectiveness ratios. | EQUATOR |
| 435 | Single study-based economic evaluation: Describe the effects of sampling uncertainty for the estimated incremental cost and incremental effectiveness parameters, together with the impact of methodological assumptions (such as discount rate, study perspective). | EQUATOR |
| 436 | Model-based economic evaluation: Describe the effects on the results of uncertainty for all input parameters, and uncertainty related to the structure of the model and assumptions. | EQUATOR |
| 437 | If applicable, report differences in costs, outcomes, or costeffectiveness that can be explained by variations between subgroups of patients with different baseline characteristics or other observed variability in effects that are not reducible by more information. | EQUATOR |
| 438 | Main findings (e.g., interpretations, inferences, and themes); might include development of a theory or model, or integration with prior research or theory | EQUATOR |
| 439 | Evidence (e.g., quotes, field notes, text excerpts, photographs) to substantiate analytic findings | EQUATOR |
| 440 | Do not repeat all the data in the tables or figures in the text; emphasize or summarize only the most important observations. | ICMJE |
| 441 | Provide data on all primary and secondary outcomes identified in the Methods Section. | ICMJE |
| 442 | Give numeric results not only as derivatives (for example, percentages) but also as the absolute numbers from which the derivatives were calculated. | ICMJE |
| 443 | Separate reporting of data by demographic variables, such as age and sex, facilitate pooling of data for subgroups across studies and should be routine, unless there are compelling reasons not to stratify reporting, which should be explained. | ICMJE |
| Discussion | | |
| 444 | Do the authors discuss any limitations of the study? | PLOS |
| 445 | If the author is disagreeing significantly with the current academic consensus, do they have a substantial case? If not, what would be required to make their case credible? | Wiley |
| 446 | Comment on general logic and on justification of interpretations and conclusions | Elsevier |
| 447 | Has previous research by the authors and others been discussed and have those results been compared to the current results? | Hindawi |
| 448 | Are limitations of the research acknowledged? | Hindawi |
| 449 | Do the authors logically explain the findings? | Taylor and Francis |
| 450 | Do the authors compare the findings with current findings in the research field? | Taylor and Francis |
| 451 | Are the implications of the findings for future research and potential applications discussed? | Taylor and Francis |
| 452 | Are any limitations of the study discussed? | Taylor and Francis |
| 453 | Are any contradictory data discussed? | Taylor and Francis |
| 454 | Review articles: Does it focus on recent advances in research? | Taylor and Francis |
| 455 | Review articles: Is the interpretation and presentation of results of previous studies accurate and precise? | Taylor and Francis |
| 456 | Interpretations of the results are appropriate; the conclusions are accurate (not misleading). | Council of Science Editors |
| 457 | Alternative interpretations for the findings are considered. | Council of Science Editors |
| 458 | Statistical differences are distinguished from meaningful differences. | Council of Science Editors |
| 459 | Personal perspectives or values related to interpretations are discussed. | Council of Science Editors |
| 460 | Practical significance or theoretical implications are discussed; guidance for future studies is offered. | Council of Science Editors |
| 461 | The study limitations are discussed. | Council of Science Editors |
| 462 | Trial limitations, addressing sources of potential bias, imprecision, and, if relevant, multiplicity of analyses | EQUATOR |
| 463 | Generalisability (external validity, applicability) of the trial findings | EQUATOR |
| 464 | Interpretation consistent with results, balancing benefits and harms, and considering other relevant evidence | EQUATOR |
| 465 | Summarize the main findings including the strength of evidence for each main outcome; consider their relevance to key groups (e.g., healthcare providers, users, and policy makers). | EQUATOR |
| 466 | Discuss limitations at study and outcome level (e.g., risk of bias), and at review-level (e.g., incomplete retrieval of identified research, reporting bias). | EQUATOR |
| 467 | Provide a general interpretation of the results in the context of other evidence, and implications for future research. | EQUATOR |
| 468 | Summarise key results with reference to study objectives | EQUATOR |
| 469 | Discuss limitations of the study, taking into account sources of potential bias or imprecision. Discuss both direction and magnitude of any potential bias | EQUATOR |
| 470 | Give a cautious overall interpretation of results considering objectives, limitations, multiplicity of analyses, results from similar studies, and other relevant evidence | EQUATOR |
| 471 | Discuss the generalisability (external validity) of the study results | EQUATOR |
| 472 | Study limitations, including sources of potential bias, statistical uncertainty, and generalisability | EQUATOR |
| 473 | Implications for practice, including the intended use and clinical role of the index test | EQUATOR |
| 474 | Strengths and limitations in your approach to this case. | EQUATOR |
| 475 | Discussion of the relevant medical literature. | EQUATOR |
| 476 | The rationale for your conclusions. | EQUATOR |
| 477 | The primary “take-away” lessons from this case report (without references) in a one paragraph conclusion. | EQUATOR |
| 478 | Key findings, including relevance to the rationale and specific aims | EQUATOR |
| 479 | Particular strengths of the project | EQUATOR |
| 480 | Nature of the association between the intervention(s) and the outcomes | EQUATOR |
| 481 | Comparison of results with findings from other publications | EQUATOR |
| 482 | Impact of the project on people and systems | EQUATOR |
| 483 | Reasons for any differences between observed and anticipated outcomes, including the influence of context | EQUATOR |
| 484 | Costs and strategic trade-offs, including opportunity costs | EQUATOR |
| 485 | Limits to the generalizability of the work | EQUATOR |
| 486 | Factors that might have limited internal validity such as confounding, bias, or imprecision in the design, methods, measurement, or analysis | EQUATOR |
| 487 | Efforts made to minimize and adjust for limitations | EQUATOR |
| 488 | The target users of the guideline are clearly defined. | EQUATOR |
| 489 | The strengths and limitations of the body of evidence are clearly described. | EQUATOR |
| 490 | There is an explicit link between the recommendations and the supporting evidence. | EQUATOR |
| 491 | The recommendations are specific and unambiguous. Describe which options are appropriate in which situations and in which population groups, as informed by the body of evidence. | EQUATOR |
| 492 | The different options for management of the condition or health issue are clearly presented. | EQUATOR |
| 493 | The guideline describes facilitators and barriers to its application. | EQUATOR |
| 494 | The guideline provides advice and/or tools on how the recommendations can be put into practice. | EQUATOR |
| 495 | The potential resource implications of applying the recommendations have been considered. | EQUATOR |
| 496 | Interpret the results, taking into account the study objectives and hypotheses, current theory and other relevant studies in the literature. | EQUATOR |
| 497 | Comment on the study limitations including potential sources of bias, limitations of the animal model, and imprecision associated with the results. | EQUATOR |
| 498 | Comment on whether, and how, the findings of this study are likely to generalise to other species or experimental conditions, including any relevance to human biology (where appropriate). | EQUATOR |
| 499 | Summarise key study findings and describe how they support the conclusions reached. Discuss limitations and the generalisability of the findings and how the findings fit with current knowledge. | EQUATOR |
| 500 | Short summary of main findings; explanation of how findings and conclusions connect to, support, elaborate on, or challenge conclusions of earlier scholarship; discussion of scope of application/generalizability; identification of unique contribution(s) to scholarship in a discipline or field | EQUATOR |
| 501 | Trustworthiness and limitations of findings | EQUATOR |
| 502 | Begin the discussion by brieﬂy summarizing the main ﬁndings, and explore possible mechanisms or explanations for these ﬁndings. | ICMJE |
| 503 | Emphasize the new and important aspects of your study and put your findings in the context of the totality of the relevant evidence. | ICMJE |
| 504 | State the limitations of your study, and explore the implications of your ﬁndings for future research and for clinical practice or policy. | ICMJE |
| 505 | Discuss the influence or association of variables, such as sex and/or gender, on your findings, where appropriate, and the limitations of the data. | ICMJE |
| 506 | Distinguish between clinical and statistical significance | ICMJE |
| 507 | State new hypotheses when warranted, but label them clearly. | ICMJE |
| 508 | Avoid claiming priority or alluding to work that has not been completed. | ICMJE |
| 509 | Provide a discussion of the importance or significance of the work, including a statement of how it adds, enhances or contradicts current beliefs and a statement of the work's potential impact on scientific and/or clinical practice—who will the work impact, and how? | Publons |
| 510 | Discuss the strengths and weaknesses of the work. Do the methods appear appropriate to the questions posed? If within your expertise, this includes sample size considerations, technical aspects and statistics. Are the conclusions supported by the data? Are the data sufficient and analyzed to support both the authors' conclusions as well as their generalizations? | Publons |
| Conclusion | | |
| 511 | Do the conclusions overreach? | PLOS |
| 512 | Are the conclusions consistent with the evidence and arguments presented? Do they address the main question posed? | Wiley |
| 513 | Do the data support the conclusions? | Wiley |
| 514 | Are the key messages short, accurate and clear? | Wiley |
| 515 | Are the conclusions and data interpretation robust, valid and reliable? | Nature |
| 516 | Whether the argument is clear and logical and the conclusions presented are supported by the results or evidence presented. | SAGE |
| 517 | Is the manuscript technically sound, and the data support the conclusion? | Wolters Kluwer Medicine |
| 518 | Comment on importance, validity and generality of conclusions | Elsevier |
| 519 | Requesting toning down of unjustified claims and generalizations | Elsevier |
| 520 | Request removal of redundancies and summaries | Elsevier |
| 521 | Do the results support the conclusions? | Hindawi |
| 522 | Are the conclusions supported by the data presented? | Taylor and Francis |
| 523 | Case reports: Are the conclusions reasonable and not attempting to generalize to wider population? | Taylor and Francis |
| 524 | Review articles: Has it a valuable contribution to the research field? | Taylor and Francis |
| 525 | Opinion articles: Is the opinion of the author well-argued? | Taylor and Francis |
| 526 | The conclusions are clearly stated; key points stand out. | Council of Science Editors |
| 527 | The conclusions follow from the design, methods, and results; justification of conclusions is well articulated. | Council of Science Editors |
| 528 | Usefulness of the work | EQUATOR |
| 529 | Sustainability | EQUATOR |
| 530 | Potential for spread to other contexts | EQUATOR |
| 531 | Implications for practice and for further study in the field | EQUATOR |
| 532 | Suggested next steps | EQUATOR |
| 533 | Key recommendations are easily identifiable. | EQUATOR |
| 534 | Link the conclusions with the goals of the study but avoid unqualified statements and conclusions not adequately supported by the data. | ICMJE |
| 535 | Are the conclusions appropriate? | Publons |
| References | | |
| 536 | Check the references in the manuscript. | PLOS |
| 537 | Does this manuscript reference previous literature appropriately? | Nature |
| 538 | Accuracy of references. | SAGE |
| 539 | Check, accuracy, number and citation appropriateness | Elsevier |
| 540 | Are there any inappropriate citations, for example, not supporting the claim being made or too many citations to the authors' own articles? | Hindawi |
| 541 | Are there any key references missing? | Taylor and Francis |
| 542 | Do the authors cite the initial discoveries where suitable? | Taylor and Francis |
| 543 | Are there places where the authors cite a review but should cite the original paper? | Taylor and Francis |
| 544 | Do the cited studies represent current knowledge? | Taylor and Francis |
| 545 | Review articles: Are any recent or important references missing? | Taylor and Francis |
| 546 | Opinion articles: Is the opinion based on current knowledge, or if it makes a big leap from current knowledge then is this logical? What supports the opinion presented? | Taylor and Francis |
| 547 | The number of references is appropriate and their selection is judicious. | Council of Science Editors |
| 548 | The review of the literature is well integrated. | Council of Science Editors |
| 549 | The references are mainly primary sources. | Council of Science Editors |
| 550 | Ideas are acknowledged appropriately (scholarly attribution) and accurately. | Council of Science Editors |
| 551 | The literature is analyzed and critically appraised. | Council of Science Editors |
| 552 | Reference citations are complete and accurate. | Council of Science Editors |
| 553 | References to papers accepted but not yet published should be designated as “in press” or “forthcoming.” Information from manuscripts submitted but not accepted should be cited in the text as “unpublished observations” with written permission from the source. | ICMJE |
| 554 | Authors should avoid citing articles in predatory or pseudo-journals. | ICMJE |
| 555 | Authors should provide direct references to original research sources whenever possible. | ICMJE |
| 556 | References should be verified using either an electronic bibliographic source, such as PubMed, or print copies from original sources. | ICMJE |
| 557 | References should be numbered consecutively in the order in which they are first mentioned in the text. Identify references in text, tables, and legends by Arabic numerals in parentheses. | ICMJE |
| 558 | References cited only in tables or figure legends should be numbered in accordance with the sequence established by the first identification in the text of the particular table or figure. | ICMJE |
| 559 | The titles of journals should be abbreviated according to the style used for MEDLINE (www.ncbi.nlm.nih.gov/nlmcatalog/journals). | ICMJE |
| 560 | References should follow the standards summarized in the NLM’s International Committee of Medical Journal Editors (ICMJE) Recommendations for the Conduct, Reporting, Editing and Publication of Scholarly Work in Medical Journals: Sample References webpage and detailed in the NLM’s Citing Medicine, 2nd edition. | ICMJE |
| Statistics | | |
| 561 | Is the statistical analysis adequate? If you do not have the expertise to consider the statistics, make sure you mention this in your report. | PLOS |
| 562 | Appropriate use of statistics and treatment of uncertainties (if applicable): all error bars should be defined in the corresponding figure legends. | Nature |
| 563 | Has the statistical analysis been performed appropriately and rigorously? | Wolters Kluwer Medicine |
| 564 | Are any statistical tests used appropriate and correctly reported? | Hindawi |
| 565 | The assumptions underlying the use of statistics are fulfilled by the data, such as measurement properties of the data and normality of distributions. | Council of Science Editors |
| 566 | Statistical tests are appropriate (optimal). | Council of Science Editors |
| 567 | If statistical analysis involves multiple tests or comparisons, proper adjustment of significance level for chance outcomes was applied. | Council of Science Editors |
| 568 | Power issues are considered in statistical studies with small sample sizes. | Council of Science Editors |
| 569 | The assumptions underlying the use of statistics are considered, given the data collected. | Council of Science Editors |
| 570 | The statistics are reported correctly and appropriately. | Council of Science Editors |
| 571 | Measures of functional significance, such as effect size or proportion of variance accounted for, accompany hypothesis-testing analyses. | Council of Science Editors |
| 572 | Provide details of the statistical methods used for each analysis, including software used. | EQUATOR |
| 573 | Describe any methods used to assess whether the data met the assumptions of the statistical approach, and what was done if the assumptions were not met. | EQUATOR |
| 574 | Describe statistical methods with enough detail to enable a knowledgeable reader with access to the original data to judge its appropriateness for the study and to verify the reported results. | ICMJE |
| 575 | When possible, quantify findings and present them with appropriate indicators of measurement error or uncertainty (such as confidence intervals). | ICMJE |
| 576 | References for the design of the study and statistical methods should be to standard works when possible (with pages stated). | ICMJE |
| 577 | Define statistical terms, abbreviations, and most symbols. | ICMJE |
| 578 | Specify the statistical software package(s) and versions used. Distinguish prespecified from exploratory analyses, including subgroup analyses. | ICMJE |
| 579 | Are the methods of statistical analysis and level of significance appropriate? | Publons |
| Tables/Figures | | |
| 580 | Are the figures and tables clear and readable?. | PLOS |
| 581 | Are the figure and table captions complete and accurate? | PLOS |
| 582 | Are the axes labeled correctly? | PLOS |
| 583 | Is the presentation appropriate for the type of data being presented? | PLOS |
| 584 | Do the figures and tables support the findings? | PLOS |
| 585 | If the paper includes tables or figures, what do they add to the paper? Do they aid understanding or are they superfluous? | Wiley |
| 586 | Comment on the number of figures, tables and schemes | Elsevier |
| 587 | Comment on any footnotes | Elsevier |
| 588 | Comment on figures, their quality and readability | Elsevier |
| 589 | Assess completeness of legends, headers and axis labels | Elsevier |
| 590 | Comment on need for colour in figures | Elsevier |
| 591 | Are the figures and tables clear and do they accurately represent the results? | Hindawi |
| 592 | Are data presented in a clear and appropriate manner? | Taylor and Francis |
| 593 | Is the presentation of tables and figures consistent with the description in text? | Taylor and Francis |
| 594 | Do the figure legends and table headings clearly explain what is shown? | Taylor and Francis |
| 595 | Do the figures and tables include measures of uncertainty, such as standard error or confidence intervals, where required as well as the sample size? | Taylor and Francis |
| 596 | Do you have any concerns about the manipulation of data? | Taylor and Francis |
| 597 | Tables, graphs, or figures are used judiciously and agree with the text. | Council of Science Editors |
| 598 | The data reported are accurate (e.g., numbers add up) and appropriate; tables and figures are used effectively and agree with the text. | Council of Science Editors |
| 599 | Restrict tables and figures to those needed to explain the argument of the paper and to assess supporting data. | ICMJE |
| 600 | Use graphs as an alternative to tables with many entries; do not duplicate data in graphs and tables. | ICMJE |
| 601 | Tables capture information concisely and display it efficiently; they also provide information at any desired level of detail and precision. | ICMJE |
| 602 | Number tables consecutively in the order of their first citation in the text and supply a title for each. | ICMJE |
| 603 | Titles in tables should be short but self-explanatory, containing information that allows readers to understand the table's content without having to go back to the text. | ICMJE |
| 604 | Give each column a short or an abbreviated heading. | ICMJE |
| 605 | Authors should place explanatory matter in footnotes, not in the heading. Explain all nonstandard abbreviations in footnotes, and use symbols to explain information if needed. | ICMJE |
| 606 | Identify statistical measures of variations, such as standard deviation and standard error of the mean. | ICMJE |
| 607 | If you use data from another published or unpublished source, obtain permission and acknowledge that source fully. | ICMJE |
| 608 | Additional tables containing backup data too extensive to publish in print may be appropriate for publication in the electronic version of the journal, deposited with an archival service, or made available to readers directly by the authors. An appropriate statement should be added to the text to inform readers that this additional information is available and where it is located. | ICMJE |
| 609 | Digital images of manuscript illustrations should be submitted in a suitable format for print publication. | ICMJE |
| 610 | For radiological and other clinical and diagnostic images, as well as pictures of pathology specimens or photomicrographs, send high-resolution photographic image files. Before-and-after images should be taken with the same intensity, direction, and color of light. | ICMJE |
| 611 | Letters, numbers, and symbols on figures should therefore be clear and consistent throughout, and large enough to remain legible when the figure is reduced for publication. | ICMJE |
| 612 | Figures should be made as self-explanatory as possible, since many will be used directly in slide presentations. Titles and detailed explanations belong in the legends—not on the illustrations themselves. | ICMJE |
| 613 | Photomicrographs should have internal scale markers. Symbols, arrows, or letters used in photomicrographs should contrast with the background. Explain the internal scale and identify the method of staining in photomicrographs. | ICMJE |
| 614 | Figures should be numbered consecutively according to the order in which they have been cited in the text. | ICMJE |
| 615 | If a figure has been published previously, acknowledge the original source and submit written permission from the copyright holder to reproduce it. Permission is required irrespective of authorship or publisher except for documents in the public domain. | ICMJE |
| 616 | In the manuscript, legends for illustrations should be on a separate page, with Arabic numerals corresponding to the illustrations. When symbols, arrows, numbers, or letters are used to identify parts of the illustrations, identify and explain each one clearly in the legend | ICMJE |
| Ethical Concerns | | |
| 617 | Is there necessary ethical approval and/or consent and was the research ethical? | Hindawi |
| 618 | Plagiarism | PLOS |
| 619 | Case reports: As far as possible, is the patient anonymous? | Taylor and Francis |
| 620 | Review articles: Is it too focused on the author’s own research? | Taylor and Francis |
| 621 | The study and results are original research | Wolters Kluwer Medicine |
| 622 | The paper has performed experiments with the highest ethical standards | Wolters Kluwer Medicine |
| 623 | The paper has not been previously published in another journal | Wolters Kluwer Medicine |
| 624 | There are no instances of plagiarism. | Council of Science Editors |
| 625 | Ideas and materials of others are correctly attributed. | Council of Science Editors |
| 626 | Prior publication by the author(s) of substantial portions of the data or study is appropriately acknowledged. | Council of Science Editors |
| 627 | There is no apparent conflict of interest. | Council of Science Editors |
| 628 | There is an explicit statement of approval by an institutional review board (IRB) for studies directly involving human subjects or data about them. | Council of Science Editors |
| 629 | The number of authors appears to be appropriate given the study. | Council of Science Editors |
| 630 | Sources and types of financial, material, and other support | EQUATOR |
| 631 | Role of study sponsor and funders, if any, in study design; collection, management, analysis, and interpretation of data; writing of the report; and the decision to submit the report for publication, including whether they will have ultimate authority over any of these activities | EQUATOR |
| 632 | Plans for seeking research ethics committee/institutional review board (REC/IRB) approval | EQUATOR |
| 633 | Plans for communicating important protocol modifications (eg, changes to eligibility criteria, outcomes, analyses) to relevant parties (eg, investigators, REC/IRBs, trial participants, trial registries, journals, regulators) | EQUATOR |
| 634 | Who will obtain informed consent or assent from potential trial participants or authorised surrogates, and how (see Item 32) | EQUATOR |
| 635 | Additional consent provisions for collection and use of participant data and biological specimens in ancillary studies, if applicable | EQUATOR |
| 636 | How personal information about potential and enrolled participants will be collected, shared, and maintained in order to protect confidentiality before, during, and after the trial | EQUATOR |
| 637 | Financial and other competing interests for principal investigators for the overall trial and each study site | EQUATOR |
| 638 | Statement of who will have access to the final trial dataset, and disclosure of contractual agreements that limit such access for investigators | EQUATOR |
| 639 | Provisions, if any, for ancillary and post-trial care, and for compensation to those who suffer harm from trial participation | EQUATOR |
| 640 | Plans for investigators and sponsor to communicate trial results to participants, healthcare professionals, the public, and other relevant groups (eg, via publication, reporting in results databases, or other data sharing arrangements), including any publication restrictions | EQUATOR |
| 641 | Authorship eligibility guidelines and any intended use of professional writers | EQUATOR |
| 642 | Plans, if any, for granting public access to the full protocol, participant-level dataset, and statistical code | EQUATOR |
| 643 | Model consent form and other related documentation given to participants and authorised surrogates | EQUATOR |
| 644 | Plans for collection, laboratory evaluation, and storage of biological specimens for genetic or molecular analysis in the current trial and for future use in ancillary studies, if applicable | EQUATOR |
| 645 | Sources of funding and other support; role of funders | EQUATOR |
| 646 | The patient should give informed consent. (Provide if requested.) | EQUATOR |
| 647 | Ethical aspects of implementing and studying the intervention(s) and how they were addressed, including, but not limited to, formal ethics review and potential conflict(s) of interest | EQUATOR |
| 648 | Sources of funding that supported this work. Role, if any, of the funding organization in the design, implementation, interpretation, and reporting | EQUATOR |
| 649 | The views of the funding body have not influenced the content of the guideline. | EQUATOR |
| 650 | Competing interests of guideline development group members have been recorded and addressed. | EQUATOR |
| 651 | Provide the name of the ethical review committee or equivalent that has approved the use of animals in this study, and any relevant licence or protocol numbers (if applicable). If ethical approval was not sought or granted, provide a justification. | EQUATOR |
| 652 | Declare any potential conflicts of interest, including financial and non-financial. If none exist, this should be stated. | EQUATOR |
| 653 | List all funding sources (including grant identifier) and the role of the funder(s) in the design, analysis and reporting of the study. | EQUATOR |
| 654 | Describe how the study was funded and the role of the funder in the identification, design, conduct, and reporting of the analysis. Describe other non-monetary sources of support. | EQUATOR |
| 655 | Describe any potential for conflict of interest of study contributors in accordance with journal policy. In the absence of a journal policy, we recommend authors comply with International Committee of Medical Journal Editors recommendations. | EQUATOR |
| 656 | Potential sources of influence or perceived influence on study conduct and conclusions; how these were managed | EQUATOR |
| 657 | Sources of funding and other support; role of funders in data collection, interpretation, and reporting | EQUATOR |
| 658 | If humans, human tissues or animals are involved, was ethics approval gained and was the study ethical? | Publons |
| Significance/Relevance | | |
| 659 | If the journal selects based on advance in the field does the study demonstrate this advance? | PLOS |
| 660 | What is the main question addressed by the research? Is it relevant and interesting? | Wiley |
| 661 | How original is the topic? What does it add to the subject area compared with other published material? | Wiley |
| 662 | Key results: the outstanding features of the work | Nature |
| 663 | Will the results advance your field in some way? If so, how much? Does the importance of the advance match the standards of the journal? | Springer |
| 664 | Will other researchers be interested in reading the study? If so, what types of researchers? Do they match the journal’s audience? Is there an alternative readership that the paper would be more suitable for? | Springer |
| 665 | Relevance to the publication | SAGE |
| 666 | Significance of the research within the field. | SAGE |
| 667 | Significance: Are the results interpreted appropriately? Are they significant? Are all conclusions justified and supported by the results? Are hypotheses and speculations carefully identified as such? | MDPI |
| 668 | Interest to the Readers: Are the conclusions interesting for the readership of the Journal? Will the paper attract a wide readership, or be of interest only to a limited number of people? (please see the Aims and Scope of the journal) | MDPI |
| 669 | Overall Merit: Is there an overall benefit to publishing this work? Does the work provide an advance towards the current knowledge? Do the authors have addressed an important long-standing question with smart experiments? | MDPI |
| 670 | Is the research original, novel and important to the field? | Elsevier |
| 671 | Ask yourself whether the manuscript should be published at all | Elsevier |
| 672 | Opinion articles: Does the article add to the discussion on a research topic? | Taylor and Francis |
| 673 | The study is relevant to the mission of the journal or its audience. | Council of Science Editors |
| 674 | The study addresses important problems or issues; the study is worth doing. | Council of Science Editors |
| 675 | The study adds to the literature already available on the subject. | Council of Science Editors |
| 676 | The study has generalizability because of the selection of subjects, setting, and educational intervention or materials. | Council of Science Editors |
| 677 | Does the study challenge existing paradigms or add to existing knowledge? | Publons |
| 678 | Does it develop novel concepts? | Publons |
| 679 | Does it matter? | Publons |
| Originality | | |
| 680 | Originality and significance: If the conclusions are not original, please provide relevant references. On a more subjective note, do you feel that the results presented are of immediate interest to many people in your own discipline, or to people from several disciplines? | Nature |
| 681 | Originality of the work conducted. It is also important to consider whether the author has ever published a substantially similar paper elsewhere. | SAGE |
| 682 | Originality/Novelty: Is the question original and well defined? Do the results provide an advance in current knowledge? | MDPI |
| 683 | Is the study innovative or original? | Publons |
| 684 | Provide a brief comment on the originality of the work, including if you know of relevant prior work. | Publons |
| Reporting | | |
| 685 | The paper adheres to the EQUATOR Network reporting guidelines | Wolters Kluwer Medicine |
| Presentation | | |
| 686 | Is the language clear and understandable? | Hindawi |
| 687 | Writing quality & clarity | PLOS |
| 688 | Is the paper well written? Is the text clear and easy to read? | Wiley |
| 689 | Is the paper an appropriate length? | Wiley |
| 690 | Should the authors consider resubmitting to the same journal after language improvements? | Wiley |
| 691 | Does the manuscript fit together well? Does it clearly describe what was done, why it was done, and what the results mean? | Springer |
| 692 | Is the manuscript written well and easy to read? If the manuscript has many mistakes, you can suggest that the authors have it checked by a native English speaker. If the language quality is so poor that it is difficult to understand, you can ask that the manuscript be corrected before you review it. | Springer |
| 693 | Technical accuracy. | SAGE |
| 694 | Structure of the paper overall, communication of main points and flow of argument. | SAGE |
| 695 | Quality of written language and structure of the article. | SAGE |
| 696 | Whether the paper is internally consistent. | SAGE |
| 697 | Quality of Presentation: Is the article written in an appropriate way? Are the data and analyses presented appropriately? Are the highest standards for presentation of the results used? | MDPI |
| 698 | English Level: Is the English language appropriate and understandable? | MDPI |
| 699 | Is the manuscript presented in an intelligible fashion and written in standard English? | Wolters Kluwer Medicine |
| 700 | Suggest changes in organization and point authors to appropriate citations | Elsevier |
| 701 | Has the appropriate structure and language been used? | Elsevier |
| 702 | List separately suggested changes in style, grammar and other small changes | Elsevier |
| 703 | Check presentation consistency | Elsevier |
| 704 | The text is well written and easy to follow. | Council of Science Editors |
| 705 | The vocabulary is appropriate. | Council of Science Editors |
| 706 | The content is complete and fully congruent. | Council of Science Editors |
| 707 | The manuscript is well organized. | Council of Science Editors |
| 708 | Use only standard abbreviations | ICMJE |
| 709 | Avoid abbreviations in the title of the manuscript. | ICMJE |
| 710 | Measurements of length, height, weight, and volume should be reported in metric units (meter, kilogram, or liter) or their decimal multiples. Temperatures should be in degrees Celsius. Blood pressures should be in millimeters of mercury, unless other units are specifically required by the journal. Journals vary in the units they use for reporting hematologic, clinical chemistry, and other measurements. | ICMJE |
| 711 | Could presentation of the results be improved and do they answer the question? | Publons |
| 712 | Address the quality of the presentation – is it clear, readable, within our guidelines? | Publons |
| Other  (Accessibility/ Article Category/ Data Availability/ Reviewer Expertise/ Journal Scope/ Terminology/ Software Availability) | | |
| 713 | The accessibility of the paper to a broad readership. | SAGE |
| 714 | Whether the work meets with the article types accepted by the journal. | SAGE |
| 715 | Does the manuscript adhere to standards in this field for data availability? | Wolters Kluwer Medicine |
| 716 | Please indicate any particular part of the manuscript, data, or analyses that you feel is outside the scope of your expertise, or that you were unable to assess fully. | Nature |
| 717 | Review articles: Is it understandable for non-expert readers? | Taylor and Francis |
| 718 | Registration number and name of trial registry | EQUATOR |
| 719 | Where the full trial protocol can be accessed, if available | EQUATOR |
| 720 | Sources of funding and other support (such as supply of drugs), role of funders | EQUATOR |
| 721 | Describe sources of funding for the systematic review and other support (e.g., supply of data); role of funders for the systematic review. | EQUATOR |
| 722 | Give the source of funding and the role of the funders for the present study and, if applicable, for the original study on which the present article is based | EQUATOR |
| 723 | Trial identifier and registry name. If not yet registered, name of intended registry | EQUATOR |
| 724 | All items from the World Health Organization Trial Registration Data Set | EQUATOR |
| 725 | Date and version identifier | EQUATOR |
| 726 | Names, affiliations, and roles of protocol contributors | EQUATOR |
| 727 | Name and contact information for the trial sponsor | EQUATOR |
| 728 | Composition, roles, and responsibilities of the coordinating centre, steering committee, endpoint adjudication committee, data management team, and other individuals or groups overseeing the trial, if applicable | EQUATOR |
| 729 | Registration number and name of registry | EQUATOR |
| 730 | Where the full study protocol can be accessed | EQUATOR |
| 731 | Provide a statement indicating whether a protocol (including the research question, key design features, and analysis plan) was prepared before the study, and if and where this protocol was registered. | EQUATOR |
| 732 | Provide a statement describing if and where study data are available. | EQUATOR |
| 733 | Does the paper fit the standards and scope of the journal it is being considered for? | Publons |
| 734 | Comment on if it matches our audience and our journal-targeted topics – does this match and why/why not? This part is best sent to the editor only. | Publons |
