# Supplementary material for: A scoping review on biomedical journal peer review guides for reviewers
Source: PLoS One. 2021 May 20;16(5):e0251440. doi: 10.1371/journal.pone.0251440 (PMC8136639; doi:10.1371/journal.pone.0251440)
Supplement: S4 File — (DOCX) [file pone.0251440.s004.docx]

**S4 File.** Quality assessment of included publications (n = 65)

|  | *Study description* | *Introduction and aims* | *Method and data* | *Data analysis* | *Ethics and bias* | *Results* | *Implications and usefulness* | *References* |
| --- | --- | --- | --- | --- | --- | --- | --- | --- |
| Ades et al. (2013) | 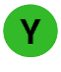 | 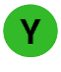 | 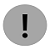 | 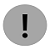 | 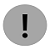 | 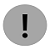 | 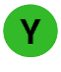 | 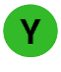 |
| Alam (2015) | 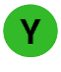 | 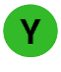 | 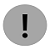 | 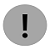 | 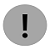 | 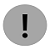 | 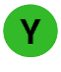 | 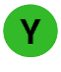 |
| Alexander (2005) | 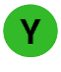 | 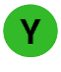 | 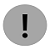 | 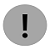 | 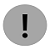 | 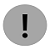 | 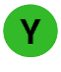 | 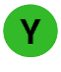 |
| Alexandrov et al. (2009) | 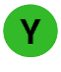 | 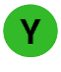 | 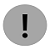 | 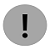 | 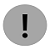 | 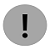 | 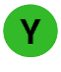 | 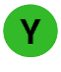 |
| Allen and Ho (2017) | 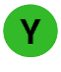 | 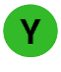 | 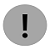 | 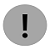 | 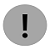 | 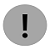 | 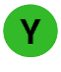 | 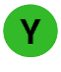 |
| Allen (2014) | 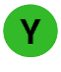 | 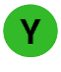 | 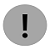 | 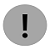 | 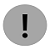 | 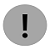 | 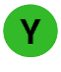 | 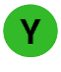 |
| Altieri and Pawlik (2020) | 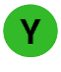 | 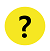 | 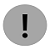 | 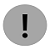 | 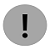 | 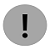 | 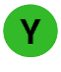 | 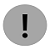 |
| Annesley (2013) | 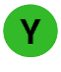 | 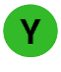 | 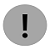 | 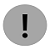 | 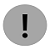 | 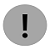 | 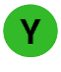 | 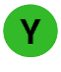 |
| Benos et al. (2003) | 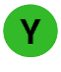 | 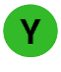 | 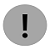 | 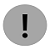 | 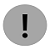 | 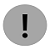 | 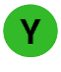 | 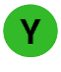 |
| Brand (2012) | 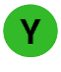 | 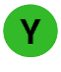 | 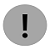 | 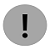 | 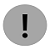 | 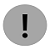 | 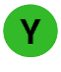 | 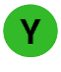 |
| Brown et al. (2017) | 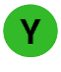 | 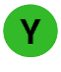 | 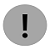 | 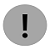 | 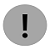 | 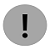 | 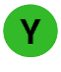 | 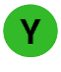 |
| Cantor et al. (2009) | 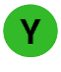 | 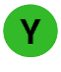 | 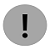 | 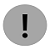 | 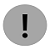 | 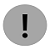 | 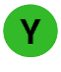 | 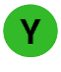 |
| Christenbery (2011) | 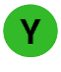 | 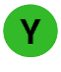 | 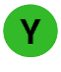 | 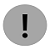 |  |  |  |  |
| Crigger (1998) |  |  |  |  |  |  |  |  |
| Currie et al. (2016) |  |  |  |  |  |  |  |  |
| Del Mar and Hoffmann (2015) |  |  |  |  |  |  |  |  |
| Dhillon (2021) |  |  |  |  |  |  |  |  |
| Duchesne (2008) |  |  |  |  |  |  |  |  |
| Duff et al. (2009) |  |  |  |  |  |  |  |  |
| Einarson and Koren (2012) |  |  |  |  |  |  |  |  |
| England and Cheng (2019) |  |  |  |  |  |  |  |  |
| Estrada et al. (2006) |  |  |  |  |  |  |  |  |
| Genter (2020) |  |  |  |  |  |  |  |  |
| Halder et al. (2011) |  |  |  |  |  |  |  |  |
| Heddle and Ness (2009) |  |  |  |  |  |  |  |  |
| Hill (2016) |  |  |  |  |  |  |  |  |
| Hunter (2020) |  |  |  |  |  |  |  |  |
| Kelly et al. (2014) |  |  |  |  |  |  |  |  |
| Kocak et al. (2020) |  |  |  |  |  |  |  |  |
| Kotis and Chung (2014) |  |  |  |  |  |  |  |  |
| Kottner and Norman (2016) |  |  |  |  |  |  |  |  |
| Kyrgidis and Triaridis (2010) |  |  |  |  |  |  |  |  |
| Lapin (2020) |  |  |  |  |  |  |  |  |
| Lazarides et al. (2020) |  |  |  |  |  |  |  |  |
| Lippi (2018) |  |  |  |  |  |  |  |  |
| Marušic et al. (2005) |  |  |  |  |  |  |  |  |
| Moher (2015) |  |  |  |  |  |  |  |  |
| Oerther and Watson (2019) |  |  |  |  |  |  |  |  |
| Pai (2020) |  |  |  |  |  |  |  |  |
| Paice (2001) |  |  |  |  |  |  |  |  |
| Pietrzak (2010) |  |  |  |  |  |  |  |  |
| Provenzale and Stanley (2005) |  |  |  |  |  |  |  |  |
| Rosenfeld (2010) |  |  |  |  |  |  |  |  |
| Rostami et al. (2011) |  |  |  |  |  |  |  |  |
| Rutkowski and Cairone (2009) |  |  |  |  |  |  |  |  |
| Salasche (1997) |  |  |  |  |  |  |  |  |
| Sasson et al. (2021) |  |  |  |  |  |  |  |  |
| Schuttpelz-Brauns et al. (2010) |  |  |  |  |  |  |  |  |
| Seals and Tanaka (2000) |  |  |  |  |  |  |  |  |
| Simpson (2008) |  |  |  |  |  |  |  |  |
| Small and Eisner (2019) |  |  |  |  |  |  |  |  |
| Smith et al. (2018) |  |  |  |  |  |  |  |  |
| Smolčić and Simundić (2014) |  |  |  |  |  |  |  |  |
| Son and Kim (2021) |  |  |  |  |  |  |  |  |
| Stahel and Moore (2016) |  |  |  |  |  |  |  |  |
| Stenfors et al. (2020) |  |  |  |  |  |  |  |  |
| Stone et al. (2018) |  |  |  |  |  |  |  |  |
| Sucato and Holland-Hall (2018) |  |  |  |  |  |  |  |  |
| Sylvia and Herbel (2001) |  |  |  |  |  |  |  |  |
| Talanow (2014) |  |  |  |  |  |  |  |  |
| Tandon (2014) |  |  |  |  |  |  |  |  |
| Tullu and Karande (2020) |  |  |  |  |  |  |  |  |
| Venne (2014) |  |  |  |  |  |  |  |  |
| Walker (1997) |  |  |  |  |  |  |  |  |
| Wilson (2002) |  |  |  |  |  |  |  |  |

Y, yes; N, no; ?, unclear; !; not applicable.

The quality assessment tool is modified from Hawker et al.’s study. The modified quality assessment tool is appended below as Appendix 1.

**Reference**

Hawker, S., Payne, S., Kerr, C., Hardey, M., & Powell, J. (2002). Appraising the Evidence: Reviewing Disparate Data Systematically. Qual Health Res, 12(9), 1284-1299. doi:10.1177/1049732302238251

**<Appendix 1 > Modified Quality Assessment Tool**

1. *Study description*. Did they provide a clear description of the study?

| Yes | No | Unclear | Not Applicable |
| --- | --- | --- | --- |

1. *Introduction and aims*. Was there a good background section and clear statement of the aims of the research?

| Yes | No | Unclear | Not Applicable |
| --- | --- | --- | --- |

1. *Method and data*. Is the method appropriate and clearly explained?

| Yes | No | Unclear | Not Applicable |
| --- | --- | --- | --- |

1. *Data analysis*. Was the description of the data analysis sufficiently rigorous?

| Yes | No | Unclear | Not Applicable |
| --- | --- | --- | --- |

1. *Ethics and bias*. Have ethical issues been addressed and has necessary ethical approval been gained?

| Yes | No | Unclear | Not Applicable |
| --- | --- | --- | --- |

1. *Results*. Is there a clear statement of the findings?

| Yes | No | Unclear | Not Applicable |
| --- | --- | --- | --- |

1. *Implications and usefulness*. Are the findings or opinions important and useful to policy or practice?

| Yes | No | Unclear | Not Applicable |
| --- | --- | --- | --- |

1. *References*. Are the findings or opinions up to date and on current knowledge?

| Yes | No | Unclear | Not Applicable |
| --- | --- | --- | --- |
