# Supplementary material for: A scoping review on biomedical journal peer review guides for reviewers
Source: PLoS One. 2021 May 20;16(5):e0251440. doi: 10.1371/journal.pone.0251440 (PMC8136639; doi:10.1371/journal.pone.0251440)
Supplement: Attachment — Submitted filename: PONE-D-20-36943_reviewercomments.pdf [file pone.0251440.s005.pdf]

PLOS ONE

A scoping review on biomedical journal peer review guides for reviewers

--Manuscript Draft--

**Manuscript Number:**	PONE-D-20-36943
**Article Type:**	Research Article
**Full Title:**	A scoping review on biomedical journal peer review guides for reviewers
**Short Title:**	Journal review guides for reviewers
**Corresponding Author:**	Lin Ang<br>Korea Institute of Oriental Medicine<br>Daejeon, KOREA, REPUBLIC OF
**Keywords:**	guide for reviewer; peer review; review guidelines; scoping review
**Abstract:**	Background: Peer review is widely used in academic field to assess a manuscript's significance and to improve its quality for publication. This scoping review will assess existing peer review guidelines and/or checklists intended for reviewers of biomedical journals and provide an overview on the review guidelines.<br>Methods: PubMed, Embase, and Allied and Complementary Medicine (AMED) databases were searched for review guidelines from the date of inception until April 2020. There was no date restriction nor article type restriction. In addition to the database search, websites of journal publishers and Council of Science Editors were additionally hand-searched.<br>Results: Of 12,757 database article records and 21 website records, 51 articles and 11 websites met inclusion criteria for the review (62 records in total). From the included records, a total of 1,197 checklist items were identified. Methods, Results, and Discussion related items were found to be the highly discussed in reviewer guidelines.<br>Conclusion: This review identified existing literature on peer review guidelines and provided an overview of the current state of peer review guides. Review guidelines were varying by journals and publishers. This calls for the need to use uniform review standards for transparent and standardized peer review.
**Order of Authors:**	Eunhye Song
	Lin Ang
**Additional Information:**	
**Question**	**Response**
**Financial Disclosure**<br><br>Enter a financial disclosure statement that describes the sources of funding for the work included in this submission. Review the submission guidelines for detailed requirements. View published research articles from *PLOS ONE* for specific examples.<br><br>This statement is required for submission and **will appear in the published article** if the submission is accepted. Please make sure it is accurate.	This work was supported by Korea Institute of Oriental Medicine (KSN20132102). The funder had no role in study design, data collection and analysis, decision to publish, or preparation of the manuscript.

Powered by Editorial Manager® and ProduXion Manager® from Aries Systems Corporation

**Unfunded studies**<br>Enter: *The author(s) received no specific funding for this work.*<br><br>**Funded studies**<br>Enter a statement with the following details:<br>• Initials of the authors who received each award<br>• Grant numbers awarded to each author<br>• The full name of each funder<br>• URL of each funder website<br>• Did the sponsors or funders play any role in the study design, data collection and analysis, decision to publish, or preparation of the manuscript?<br>• **NO** - Include this sentence at the end of your statement: *The funders had no role in study design, data collection and analysis, decision to publish, or preparation of the manuscript.*<br>• **YES** - Specify the role(s) played.<br><br>* typeset	
**Competing Interests**<br><br>Use the instructions below to enter a competing interest statement for this submission. On behalf of all authors, disclose any competing interests that could be perceived to bias this work—acknowledging all financial support and any other relevant financial or non-financial competing interests.<br><br>This statement **will appear in the published article** if the submission is accepted. Please make sure it is accurate. View published research articles from *PLOS ONE* for specific examples.	The authors have declared that no competing interests exist.

**NO authors have competing interests**<br><br>Enter: *The authors have declared that no competing interests exist.*<br><br>**Authors with competing interests**<br><br>Enter competing interest details beginning with this statement:<br><br>*I have read the journal's policy and the authors of this manuscript have the following competing interests: [insert competing interests here]*<br><br>* typeset	
**Ethics Statement**<br><br>Enter an ethics statement for this submission. This statement is required if the study involved:<br><br>• Human participants<br>• Human specimens or tissue<br>• Vertebrate animals or cephalopods<br>• Vertebrate embryos or tissues<br>• Field research<br><br>Write "N/A" if the submission does not require an ethics statement.<br><br>General guidance is provided below. Consult the submission guidelines for detailed instructions. **Make sure that all information entered here is included in the Methods section of the manuscript.**	N/A

**Format for specific study types**<br><br>**Human Subject Research (involving human participants and/or tissue)**<br>• Give the name of the institutional review board or ethics committee that approved the study<br>• Include the approval number and/or a statement indicating approval of this research<br>• Indicate the form of consent obtained (written/oral) or the reason that consent was not obtained (e.g. the data were analyzed anonymously)<br><br>**Animal Research (involving vertebrate animals, embryos or tissues)**<br>• Provide the name of the Institutional Animal Care and Use Committee (IACUC) or other relevant ethics board that reviewed the study protocol, and indicate whether they approved this research or granted a formal waiver of ethical approval<br>• Include an approval number if one was obtained<br>• If the study involved *non-human primates*, add *additional details* about animal welfare and steps taken to ameliorate suffering<br>• If anesthesia, euthanasia, or any kind of animal sacrifice is part of the study, include briefly which substances and/or methods were applied<br><br>**Field Research**<br><br>Include the following details if this study involves the collection of plant, animal, or other materials from a natural setting:<br>• Field permit number<br>• Name of the institution or relevant body that granted permission	
**Data Availability**<br><br>Authors are required to make all data underlying the findings described fully available, without restriction, and from the time of publication. PLOS allows rare exceptions to address legal and ethical concerns. See the PLOS Data Policy and FAQ for detailed information.	Yes - all data are fully available without restriction

A Data Availability Statement describing where the data can be found is required at submission. Your answers to this question constitute the Data Availability Statement and **will be published in the article**, if accepted.<br><br>**Important:** Stating 'data available on request from the author' is not sufficient. If your data are only available upon request, select 'No' for the first question and explain your exceptional situation in the text box.<br><br>Do the authors confirm that all data underlying the findings described in their manuscript are fully available without restriction?	
**Describe where the data may be found in full sentences. If you are copying our sample text, replace any instances of XXX with the appropriate details.**<br><br>• If the data are **held or will be held in a public repository**, include URLs, accession numbers or DOIs. If this information will only be available after acceptance, indicate this by ticking the box below. For example: *All XXX files are available from the XXX database (accession number(s) XXX, XXX.*).<br>• If the data are all contained **within the manuscript and/or Supporting Information files**, enter the following: *All relevant data are within the manuscript and its Supporting Information files.*<br>• If neither of these applies but you are able to provide **details of access elsewhere**, with or without limitations, please do so. For example:<br><br>*Data cannot be shared publicly because of [XXX]. Data are available from the XXX Institutional Data Access / Ethics Committee (contact via XXX) for researchers who meet the criteria for access to confidential data.*<br><br>*The data underlying the results presented in the study are available from (include the name of the third party*	All relevant data are within the manuscript and its Supporting Information files.

and contact information or URL).<br>• This text is appropriate if the data are owned by a third party and authors do not have permission to share the data.<br><br>* typeset	
Additional data availability information:	

Manuscript Click here to access/download;Manuscript;Scoping review_manuscript.docx

A scoping review on biomedical journal peer review guides for reviewers

Eunhye Song,[1] Lin Ang,[2,3] Ji-Yeun Park,[4] Eun-Young Jun,[5] Kyeong Han Kim,[6] Jihee Jun,[2] Sunju Park,[7,*] Myeong Soo Lee [2,3,*]

1 Global Strategy Division, Korea Institute of Oriental Medicine, Daejeon, Korea

2 Clinical Medicine Division, Korea Institute of Oriental Medicine, Daejeon, Korea

3 Korean Convergence Medicine, University of Science and Technology, Daejeon, Korea

4 Department of Bioconvergence Sciences, College of Korean Medicine, Daejeon University, Daejeon, Korea

5 Department of Nursing, Daejeon University, Daejeon, Korea

6 Department of Preventive Medicine, College of Korean Medicine, Woosuk University, Jeonju, Republic of Korea

7 Department of Preventive Medicine, College of Korean Medicine, Daejeon University, Daejeon, Korea

* Corresponding authors:

sjpark@dju.kr (Sunju Park); drmslee@gmail.com (Myeong Soo Lee)

Short running title: Journal review guides for reviewers

Abstract

Background: Peer review is widely used in academic ~~field~~ to assess a manuscript's significance and to improve its quality for publication. This scoping review will assess existing peer review guidelines and/or checklists intended for reviewers of biomedical journals and provide an overview on the review guidelines.

Methods: PubMed, Embase, and Allied and Complementary Medicine (AMED) databases were searched for review guidelines from the date of inception until April 2020. There was no date restriction nor article type restriction. In addition to the database search, websites of journal publishers and Council of Science Editors were additionally hand-searched.

Results: Of 12,757 database article records and 21 website records, 51 articles and 11 websites met inclusion criteria for the review (62 records in total). From the included records, a total of 1,197 checklist items were identified. Methods, Results, and Discussion related items were found to be the highly discussed in reviewer guidelines.

Conclusion: This review identified existing literature on peer review guidelines and provided an overview of the current state of peer review guides. Review guidelines were varying by journals and publishers. This calls for the need to use uniform review standards for transparent and standardized peer review.

Protocol registration: The protocol for this study has been registered at Research Registry (www.researchregistry.com): reviewregistry881.

Keywords: guide for reviewer, peer review, review guidelines, scoping review

1. Background

In academic ~~field,~~ peer review is a fundamental part of assessing a manuscript's potential significance to the field and improving its methodological rigor and its overall quality for publishing in scholarly journals. Editors depend on reviewers to make their editorial decisions since the reviewers have expertise in the subject areas of submissions they are reviewing. In terms of writing a paper, there have been many efforts to address inadequate reporting of researches and to achieve the standard in scientific writing through transparent reporting of researches [1]. Besides these guidelines, almost all journals, if not all, provide guidelines for authors at their websites. In recent years, guidelines and checklists have been disseminated by journals and publishers for each of the major study types to ensure consistent and complete reporting [2]. However, guidelines for reviewers are not easily discovered. In a survey of 116 health research journals on the McMaster list, Hirst and Altman found only 41 journals (35%) to be providing online instructions to peer reviewers [3]. Ensuring consistent and complete reviewing ~~are~~ also needed. Publishers like Elsevier, SAGE, Springer Nature, and Wiley provide openly available guidelines for reviewers to assist them through the review process, in order to maintain and support standards in reviewing. Yet, not all publishers provide such guidance to their reviewers openly.

Scoping reviews are similar to systematic reviews in the way they systematically examine evidence and the way they are conducted, but they differ from each other in their purpose. The main differences relate to critical appraisal or risk of bias, results synthesis, and study inclusion. Critical appraisal or risk of bias assessment is necessary for systematic reviews, while it is optional for scoping reviews. Systematic reviews synthesize findings from each included studies using meta-analysis or meta-synthesis, while scoping reviews do not synthesize findings [4, 5]. Scoping reviews include more papers broadly to provide an overview of the existing literature regardless of their type or quality [6]. Systematic reviews are used as references for policymaking or clinical practice as they are suitable for clearly defined questions relating to an intervention efficacy, but scoping reviews are descriptive and suitable for examining evidence in a broader sense such as identifying characteristics of the evidence on a topic [7, 8] or identifying available evidence in a field [4].

Currently, there are no widely used guidelines for peer reviewing manuscripts. This may cause lead to difficulty in reviewing so reviewers may be uncertain about what are expected from them. This scoping review will determine existing peer review guidelines and/or checklists intended for reviewers of biomedical journals. The purpose of this study is to determine whether there are standards on how reviewers of biomedical journals should perform peer reviews and write their review reports, and to provide an overview on the review guidelines.

2. Materials and methods

A scoping review on the manuscript peer review guidelines for reviewers of biomedical journals were conducted to identify available evidence on peer review guidelines for reviewers and to determine what is known about how to peer review. In order to improve the methodological and reporting quality of this scoping review, the checklist of PRISMA Extension for Scoping Reviews (PRISMA-ScR), developed in 2018 by the EQUATOR Network was used (S1 File)[7]

2.1. Registration

The protocol for this study has been registered at Research Registry with its unique identifying number, reviewregistry881 in April 2020.

2.2. Databases and search strategy

PudMed, Embase, and Allied and Complementary Medicine Database (AMED) were searched from the date of inception until April 2020. In the search, there was no date restriction. Reviewer guidelines by some of the major publishers and journals were additionally searched online and collected.

For comprehensive search on peer review guidelines in biomedical field, the following search terms were used: “reviewing”, “reviewer”, “peer review”, “peer reviewer”, “journal”, “manuscript”, “guide”, “guidelines”, “how to”, “lesson”, “tips”, “advice”, “recommendation”, “report form”, and “checklist”. The complete search strategy can be found in S2.

For grey literature, websites of major biomedical and complementary medicine journal publishers including the following were searched: Elsevier (www.elsevier.com), Springer (www.springer.com), Nature (www.nature.com), Wiley (www.wiley.com), Wolters Kluwer (www.wolterskluwer.com), Taylor and Francis (www.tandfonline.com), SAGE (www.sagepub.com), PLOS (www.plos.org), and

MDPI (www.mdpi.com), Hindawi (www.hindawi.com), Thieme (www.thieme.com), Mary Ann Liebert (www.liebertpub.com), Cognizant Communication Corporation, InnoVision Health Media, World Scientific Publishing, BioMed Central, S. Karger AG, Lippincott Williams and Wilkins. In addition, the website of Council of Science Editors (www.councilscienceeditors.org) was also searched.

2.3. Inclusion and exclusion criteria

The inclusion criteria for this study were any article relating to guidelines or checklists for peer reviewing. All types of articles in English were eligible for inclusion in this study without restrictions on date or study design. Thus, editorial materials such as editorial and tutorials were included. Review checklists on websites of above mentioned publishers and Council of Science Editors were also included.

The exclusion criteria were statistical review, conference abstracts, non-English articles, checklists for authors, review process, reviewer/editor roles or behaviors, assessing quality or reliability, review challenges or issues, and not relating to manuscript peer review. Website resources by the publishers of SCIE CAM journals that do not provide reviewer guidelines were not eligible.

2.4. Outcomes

The primary outcomes were review criteria or checklist items such as a list of questions to consider during peer review. The secondary outcomes were reviewer guidelines such as instructions or recommendations on peer review structure, review contents, reviewer behavior, review ethics, review resources, and review management.

2.5. Study selection and data extraction

A data extraction form was developed for this scoping review for data charting purposes. The data

extracted from the included articles were first author’s last name, publication year, article type, guidance criteria, and number of checklist items. The review guidance criteria were also developed for grouping the contents of reviewer guidelines to provide a clear overview of available guidelines for reviewers.

Two independent review authors searched the databases and assessed the eligibility of the searched records. Any discrepancies on article inclusion were discussed with a third review author and resolved on consensus.

All checklist items for reviewing manuscripts were extracted into an Excel spreadsheet. Each item was identified and categorized by its relevant section in manuscript review. The section categories used were title, abstract, introduction, methods, results, discussion, conclusion, references, tables and figures, presentations, and others.

3. Results

3.1. Search results

After screening and excluding articles and other records that fell under exclusion criteria, a total of 62 records (51 articles on peer review guidelines and 11 publisher and council websites with reviewer guidelines) were included for this study (Fig. 1). Out of 62 records, 16 articles did not provide a checklist as in what to look for in the manuscript and only provided guidance for reviewers as in how to review. The 51 included articles and the 11 websites contained 926 items and 271 items respectively (S3 File), which amounted to 1197 items in total.

Fig. 1. Flow diagram for the review.

3.2. Characteristics of included articles and websites

The descriptive characteristics of the included articles and websites are summarized in Table 1 and Table 2 respectively. Article types of the included studies were mostly editorial materials (n=40; 78.4%) such as tutorial, editorial, special articles, perspective, interview, special contribution, publishing series and commentary. Other types included were review articles (n=9; 17.6%), research article (n=1; 2.0%), and guidelines (n=1; 2.0%). Among the included articles that provided information on how to peer review, 35 articles (68.6%) provided a checklist questions or criteria, but 16 articles (31.4%) did not provide a checklist questions or criteria.

Table 1. Characteristics of included articles – Database search results (51 articles, 926 items).

Author & Year	Article Type	Guidance Criteria[#]	No. of Checklist Items
Ades, 2013 [9]	Tutorial	C	42
Alam, 2015 [2]	Editorial	A, C, M, R, S	NP

Alexander, 2005 [10]	Editorial	C, S	19
Alexandrov, 2009 [11]	Special Article	A, C, M, RE, S	7
Allen, 2017 [12]	Editor's Perspective	A, C, M, R, S	10
Allen, 2014 [13]	Scientific Perspectives	A, C, M, RE, S	21
Altieri, 2020 [14]	Interview	B, C, R, S	NP
Annesley, 2013 [15]	Tutorial*	A, C, S	NP
Benos, 2003 [16]	A Personal View	A, C, M, R, RE, SE	14
Brand, 2012 [17]	Editorial	A, C, RE, S	31
Cantor, 2009 [18]	Editorial	B, C, RE, S	NP
Christenbery, 2011 [19]	Education	A, C, M, O, RE, SE	36
Crigger, 1998 [20]	Editorial	A, M, RE	NP
Currie, 2016 [21]	Special contribution	C, M, RE, S, SE	NP
Del Mar, 2015 [22]	Tutorial	A, C, M, RE, S, SE	NP
Duchesne, 2008 [23]	Research	C	70
Duff, 2009 [24]	Commentary	A, B, C, M, O, RE, S	NP
Einarson, 2012 [25]	Review*	A, C, S	NP
England, 2019 [26]	Review	C	14
Estrada, 2006 [27]	Editorial*	A, M, S	11
Halder, 2011 [28]	Review	A, B, C, M, R, RE, S	NP
Heddle, 2009 [29]	Clinical research focus	A, C, M, RE, S	16
Hill, 2016 [30]	Editorial*	A, C, RE, S	8
Kotsis, 2014 [31]	Review*	C, M, RE, S	37
Kottner, 2016 [32]	Editorial	A, C, M, R, RE, S	NP
Kyrgidis, 2010 [33]	Review	C, R, RE	16
Lippi, 2018 [34]	Perspective	A, C, M, RE, S	20
Marusic, 2005 [35]	Guidelines	A, C, M, RE, S	23
Moher, 2015 [36]	Tutorial	A, C, S	NP
Oerther, 2019 [37]	Editorial	A, B, C, M, RE, S	31
Paice, 2001 [38]	Medical publishing series	B, C, M, RE, S	13
Pietrzak, 2010 [39]	Special editorial	B, C, M, RE, S	NP
Provenzale, 2005 [40]	Perspective	A, C, RE	35
Rosenfeld, 2010 [41]	Special Article	A, C, M, RE, S	57
Rostami, 2011 [42]	Medical education	C, M, R, RE, S	18
Rutkowski, 2009 [43]	Editorial	A, C, M, RE, S	NP
Salasche, 1997 [44]	Editorial	B, C, M, RE, S	NP
Schuttpelz-Brauns, 2010 [45]	Position paper	A, C, S	9
Seals, 2000 [46]	Innovation and ideas	C, S	54
Simpson, 2008 [47]	Tutorial*	A, C, M, S	37
Smith, 2019 [48]	Review	C, M, S	34

Smolcic, 2014 [49]	Research integrity corner	A, C, M, RE, S	65
Stahel, 2016 [50]	Tutorial	A, C, M, O, R, S	18
Stone, 2018 [51]	Editorial	C, S	9
Sucato, 2018 [52]	Review	A, C, M, R, RE, S	40
Sylvia, 2001 [53]	Special article	A, B, C, R, RE, S	13
Talanow, 2014 [54]	Editorial	C	10
Tandon, 2014 [55]	Review	C, M, S	37
Venne, 2015 [56]	Professional Issues	A, C, M, RE, S	NP
Walker, 1997 [57]	Tutorial*	C	40
Wilson, 2002 [58]	Review*	C	11

A: Attitude; B: Becoming a Reviewer; C: Contents; M: Management; R: Resources; RE: Reviewer Ethics; S: Structure; SE: Study Ethics; O: Other.

NP: not provided.

* Article type not reported; assumed article type added.

Table 2. Characteristics of included websites – Hand search results (11 websites, 271 items).

Website Source	Organization Type	No. of Checklist Items
Council of Science Editors	Council	76
Elsevier	Publisher	29
Hindawi	Publisher	14
MDPI	Publisher	7
Nature	Publisher	10
PLOS	Publisher	27
SAGE	Publisher	15
Springer	Publisher	7
Taylor and Francis	Publisher	64
Wiley	Publisher	14
Wolters Kluwer Medicine	Publisher	8

3.3. Categories of reviewer guidelines

The suggestions and instructions for reviewers provided in the included ~~articles~~ were grouped into 9 categories: structure, contents, attitude, management, becoming a reviewer, resources, reviewer ethics,

study ethics, and others (Fig. 2). Articles were determined to fit into one or more of the categories as appropriate. Structure category was assigned to articles with guidelines on how to organize or write the reviewer comments. Contents category was assigned to articles with guidelines on how to assess significance, relevance, and soundness of contents like introduction, methodology, results, discussion, and conclusion. Attitude category was assigned to articles with guidelines on how to behave toward authors or how to keep the positive and constructive tone. Management category was assigned to articles with guidelines on how to manage or deal with reviewing time. Becoming-a-reviewer category was assigned to articles with guidelines on how to sign up to become a reviewer. Resources category was assigned for 11 articles with guidelines on how to access or refer to some resources related to reviewing. Reviewer ethics category was assigned for 30 articles with guidelines on how to deal with any possible reviewer ethics. Study ethics category was assigned for 4 articles with guidelines on how to deal with ethical concerns in manuscripts the reviewers review. Others category was assigned for 3 articles with guidelines on signing reviews for increased transparency and reviewer recognition.

Fig. 2. Frequency of reviewer guides by category (n=51).

3.4. Categories of reviewer checklist items

The checklist items were extracted from the checklists from the included articles and web pages, and the items were grouped into 17 categories (Table 3). Top 3 categories with a high number of items were Methods (334 items, 27.9%), Results (125 items, 10.4%), and Discussion (110 items, 9.2%) from article items and website items combined. Looking into them by articles and websites, the top 3 among article items were also Methods (274 items, 29.6%), Results (103 items, 11.1%), and Discussion (92 items, 9.9%); the top 3 among website items differed slightly that besides Methods (60 items, 22.1%) and Results (22 items, 8.1%), Presentation related items were among the top 3 with 8.1% (22 items).

The grouped categories of the identified checklist items and their numbers are shown in a graphical representation (Fig. 3). In the figure, the darkness of color and the size of diamond shape are associated with the number of items for each category.

Table 3. Extracted checklist items by categories (1197 items; 17 categories).

Category	Article Items		Website Items		Total	
	**n**	**%**	**n**	**%**	**n**	**%**
Title	20	2.2	8	3.0	28	2.3
Abstract	44	4.8	18	6.6	62	5.2
Keywords	4	0.4	1	0.4	5	0.4
Introduction	75	8.1	18	6.6	93	7.8
**Methods**	**279**	**29.6**	**60**	**22.1**	**334**	**27.9**
**Results**	**107**	**11.1**	**22**	**8.1**	**125**	**10.4**
**Discussion**	**91**	**9.9**	18	6.6	**110**	**9.2**
Conclusion	33	3.6	17	6.3	50	4.2
References	39	4.2	17	6.3	56	4.7
Statistics	29	3.2	11	4.1	41	3.4
Tables/Figures	56	6.3	19	7.0	77	6.4
Ethical Concerns	42	5.1	13	4.8	60	5.0
Significance/Relevance	43	4.6	18	6.6	61	5.1
Originality	16	1.7	3	1.1	19	1.6
Reporting	3	0.3	1	0.4	4	0.3
**Presentation**	37	4.0	**22**	**8.1**	59	4.9
Other*	8	0.9	5	1.8	13	1.1
**Total**	**926**	**100.0**	**272**	**100.0**	**1197**	**100.0**

* Other: Accessibility, Article Category, Data Availability, Journal Scope, Reviewer Expertise, Software Availability, and Terminology.

Bold values indicates top 3 categories among Articles, Websites, and Total (Articles & Websites) items.

Fig. 3. Graphical representation of the review (1197 items; 17 categories).

4. Discussion

From an extensive range of sources, the scoping review produced a comprehensive list of items for conducting peer review. Review guidelines are important for high quality review, especially since editors rely on reviewers and peer reviewer recommendations have an influence on editorial decisions [59]. Such influence of reviewer recommendations on editorial decisions becomes an issue when the quality of peer review is doubtful or when reviewer bias is present [60]. In order to prevent reviewer bias and better improve the peer review quality, the journals and publishers published and posted reviewer guidelines.

Categories with the two highest frequencies were Contents (96.1%) and Structure (78.4%). These are considered important parts of peer review that many included articles were highly focused on them. They reflect the quality of the articles inside (contents) and out (structure). However, bias frequently occurred reviewing them which eventually affects editors and editorial decisions [61, 62].

Although the reporting guidelines by the EQUATOR network are now widely accepted by major publishers and journals, they are most used by authors in their reporting as the main purpose of those guidelines was to improve the reporting by authors. For this reason, guidelines by publishers or journals placed much less significance on reporting guidelines for reviewers.

These days, being transparent and open about peer review process has gained more attention and is a critical issue [63]. Although reviewers claim that their review reports were not affected by being open peer review, open peer review journals struggles to get reviewers [64]. Manuscript peer reviews could be improved by increasing the consistency of ratings, narrative comments, and recommendations [65]. Having standards in peer reviewing could make reviewers more at ease and simplify their review. An uniform review guideline checklist would support the transparent peer review to be more transparent and standardized. It would also help authors to better understand their insufficient parts of the manuscript.

Limitation of this study was inclusion of mainly editorial materials. Most included studies being editorial materials places concerns for the guidelines and checklists suggested, since editorials are generally not peer reviewed. Scoping reviews cover a wide range of article types that quality assessment is not mandatory to perform [4, 6]. In this study, the articles on "how to review" were mostly editorial materials, including editorials, tutorials, and special contributions, and the generally used quality assessment tools could not be applied to them and quality assessment were not performed. In addition, there were limitations in extracting more grey literatures. Many journals do not provide reviewer checklists openly and they cannot be accessed by anyone. Thus checklists provided by publishers were only considered.

5. Conclusion

This scoping review explored peer reviewer guidelines in biomedical journals. It identified the extent of existing literature on peer review guidelines and provided an overview of peer review guidelines. It demonstrated the current state of peer review guidelines that they were not uniform which may imply the need for more uniform guidelines. This review could provide grounds for future studies related to reviewer guidelines.

Declarations

Ethics approval and consent to participate

Not applicable

Consent for publication

Not applicable

Availability of data and materials

All data generated or analyzed during this study are included in this published article and its supplementary information files.

Competing interests

The authors declare that they have no competing interests.

Funding

This work was supported by Korea Institute of Oriental Medicine (KSN20132102). The funder had no role in study design, data collection and analysis, decision to publish, or preparation of the manuscript.

Authors' contributions

MSL and SP conceptualized the review. ES wrote the draft. ES and LA performed the search. ES, LA, and JJ analyzed the data. J-YP, E-YJ, KHK, SP, and MSL critically reviewed and edited the manuscript. All authors read and approved the final manuscript.

Acknowledgements

Not applicable

References

1. Simera I, Altman DG. Reporting medical research. International journal of clinical practice. 2013;67(8):710-6. Epub 2013/05/10. doi: 10.1111/ijcp.12168. PubMed PMID: 23656235.

2. Alam M. How to review a manuscript. Dermatologic surgery : official publication for American Society for Dermatologic Surgery [et al]. 2015;41(8):883-8. Epub 2015/07/29. doi: 10.1097/dss.0000000000000421. PubMed PMID: 26218722.

3. Hirst A, Altman DG. Are peer reviewers encouraged to use reporting guidelines? A survey of 116 health research journals. PloS one. 2012;7(4):e35621. Epub 2012/05/05. doi: 10.1371/journal.pone.0035621. PubMed PMID: 22558178; PubMed Central PMCID: PMCPMC3338712 member of several reporting guideline development groups including CONSORT, PRISMA, STROBE, ARRIVE and STRICTA. AH is employed as a research fellow by the EQUATOR Network. This does not alter the authors' adherence to all PLoS ONE policies on sharing data and materials.

4. Munn Z, Peters MDJ, Stern C, Tufanaru C, McArthur A, Aromataris E. Systematic review or scoping review? Guidance for authors when choosing between a systematic or scoping review approach. BMC Medical Research Methodology. 2018;18(1):143. doi: 10.1186/s12874-018-0611-x.

5. Lockwood C, dos Santos KB, Pap R. Practical Guidance for Knowledge Synthesis: Scoping Review Methods. Asian Nursing Research. 2019;13(5):287-94. doi: https://doi.org/10.1016/j.anr.2019.11.002.

6. Peters MD, Godfrey CM, Khalil H, McInerney P, Parker D, Soares CB. Guidance for conducting systematic scoping reviews. International journal of evidence-based healthcare. 2015;13(3):141-6. Epub 2015/07/03. doi: 10.1097/xeb.0000000000000050.

7. Tricco AC, Lillie E, Zarin W, O'Brien KK, Colquhoun H, Levac D, et al. PRISMA Extension for Scoping Reviews (PRISMA-ScR): Checklist and Explanation. Annals of internal medicine. 2018;169(7):467-73. doi: 10.7326/m18-0850.

8. Sucharew H, Macaluso M. Methods for Research Evidence Synthesis: The Scoping Review Approach. J Hosp Med. 2019;7:3. doi: doi:10.12788/jhm.3248

9. Ades AE, Caldwell DM, Reken S, Welton NJ, Sutton AJ, Dias S. Evidence synthesis for decision making 7: a reviewer's checklist. Medical decision making : an international journal of the Society for Medical Decision Making. 2013;33(5):679-91. doi: 10.1177/0272989x13485156. PubMed PMID: 23804511; PubMed Central PMCID: PMCPMC3704204.

10. Alexander GR. A guide to reviewing manuscripts. Maternal and child health journal. 2005;9(1):113-7. doi: 10.1007/s10995-005-2423-y.

11. Alexandrov AV, Hennerici MG, Norrving B. Suggestions for reviewing manuscripts. Cerebrovascular diseases (Basel, Switzerland). 2009;28(3):243-6. doi: 10.1159/000228588.

12. Allen LA, Ho PM. Peer Review of a Manuscript Submission: A How-To Guide for Effective and Efficient Commentary. Circulation Cardiovascular quality and outcomes. 2017;10(12). doi: 10.1161/circoutcomes.117.004446.

13. Allen TW. Conducting Proper Peer Review for a Journal. Bariatric Surgical Practice and

Patient Care. 2014;9(1):18-20. doi: 10.1089/bari.2014.9967.

14. Altieri MS, Pawlik TM. Mentor of the Month Series: How to Review a Manuscript from an Editor's Perspective? Journal of gastrointestinal surgery : official journal of the Society for Surgery of the Alimentary Tract. 2020. doi: 10.1007/s11605-020-04567-w.

15. Annesley TM. Writing an Effective Manuscript Review: The 6 “Be's” to Success. Clinical chemistry. 2013;59(7):1028-35. doi: 10.1373/clinchem.2013.208280.

16. Benos DJ, Kirk KL, Hall JE. How to review a paper. Advances in physiology education. 2003;27(1-4):47-52. doi: 10.1152/advan.00057.2002.

17. Brand RA. Reviewing for clinical orthopaedics and related research. Clinical orthopaedics and related research. 2012;470(9):2622-5. doi: 10.1007/s11999-012-2447-8.

18. Cantor GH, Caswell JL, Crissman JW, Gillette DM, Gunson DE, Hogenesch H, et al. Veterinary Pathology and Peer Review. Veterinary Pathology. 2009;46(2):173-5. doi: 10.1354/vp.46-2-173.

19. Christenbery TL. Manuscript peer review: a guide for advanced practice nurses. Journal of the American Academy of Nurse Practitioners. 2011;23(1):15-22. doi: 10.1111/j.1745-7599.2010.00572.x.

20. Crigger NJ. What We Owe The Author: rethinking editorial peer review. Nursing ethics. 1998;5(5):451-8. doi: 10.1177/096973309800500508.

21. Currie G, McCuaig C, Di Prospero L. Systematically Reviewing a Journal Manuscript: A Guideline for Health Reviewers. Journal of medical imaging and radiation sciences. 2016;47(2):129-38.e3. doi: 10.1016/j.jmir.2016.01.005.

22. Del Mar C, Hoffmann TC. A guide to performing a peer review of randomised controlled trials. BMC Med. 2015;13:248. doi: 10.1186/s12916-015-0471-8.

23. Duchesne S, Jannin P. Proposing a manuscript peer-review checklist. NeuroImage. 2008;39(4):1783-7. doi: 10.1016/j.neuroimage.2007.09.065.

24. Duff K, O'Bryant SE, Westervelt HJ, Sweet JJ, Reynolds CR, van Gorp WG, et al. On becoming a peer reviewer for a neuropsychology journal. Archives of clinical neuropsychology : the official journal of the National Academy of Neuropsychologists. 2009;24(3):201-7. doi: 10.1093/arclin/acp031.

25. Einarson TR, Koren G. To accept or reject? A guide to peer reviewing of medical journal papers. Journal of population therapeutics and clinical pharmacology = Journal de la therapeutique des populations et de la pharmacologie clinique. 2012;19(2):e328-33.

26. England JR, Cheng PM. Artificial Intelligence for Medical Image Analysis: A Guide for Authors and Reviewers. AJR American journal of roentgenology. 2019;212(3):513-9. doi: 10.2214/ajr.18.20490.

27. Estrada C, Kalet A, Smith W, Chin MH. How to be an outstanding reviewer for the Journal of General Internal Medicine … and other journals. Journal of general internal medicine. 2006;21(3):281. doi: 10.1111/j.1525-1497.2006.00354.x.

28. Halder N, Ramsay R, Tyrer P, Casey P. Peer reviewing made easy. Advances in Psychiatric

Treatment. 2011;17(2):150-7. doi: 10.1192/apt.bp.109.007294.

29. Heddle NM, Ness PM. Reviewing manuscripts: tips and responsibilities. Transfusion. 2009;49(11):2265-8. doi: 10.1111/j.1537-2995.2009.02390.x.

30. Hill JA. How to Review a Manuscript. Journal of electrocardiology. 2016;49(2):109-11. doi: 10.1016/j.jelectrocard.2016.01.001.

31. Kotsis SV, Chung KC. Manuscript rejection: how to submit a revision and tips on being a good peer reviewer. Plastic and reconstructive surgery. 2014;133(4):958-64. doi: 10.1097/prs.0000000000000002.

32. Kottner J, Norman I. How to peer review and revise manuscripts submitted for publication in academic nursing journals. International journal of nursing studies. 2016;64:A1-a3. doi: 10.1016/j.ijnurstu.2016.09.011.

33. Kyrgidis A, Triaridis S. Methods and Biostatistics: a concise guide for peer reviewers. Hippokratia. 2010;14(Suppl 1):13-22.

34. Lippi G. How do I peer-review a scientific article?-a personal perspective. Annals of translational medicine. 2018;6(3):68. doi: 10.21037/atm.2017.12.15.

35. Marusić M, Sambunjak D, Marusić A. Guide for peer reviewers of scientific articles in the Croatian Medical Journal. Croat Med J. 2005;46(2):326-32.

36. Moher D. Optimal strategies to consider when peer reviewing a systematic review and meta-analysis. BMC Medicine. 2015;13(1):274. doi: 10.1186/s12916-015-0509-y.

37. Oerther S, Watson R. Emerging nursing scholars guide to peer reviewing an academic manuscript. Nursing open. 2019;6(4):1284-8. doi: 10.1002/nop2.368.

38. Paice E. How to write a peer review. Hospital medicine (London, England : 1998). 2001;62(3):172-5. doi: 10.12968/hosp.2001.62.3.2392.

39. Pietrzak WS. A practical guide to effective journal peer reviewing. The Journal of craniofacial surgery. 2010;21(3):631-6. doi: 10.1097/SCS.0b013e3181d8403c.

40. Provenzale JM, Stanley RJ. A systematic guide to reviewing a manuscript. AJR American journal of roentgenology. 2005;185(4):848-54. doi: 10.2214/ajr.05.0782.

41. Rosenfeld RM. How to review journal manuscripts. Otolaryngology--head and neck surgery : official journal of American Academy of Otolaryngology-Head and Neck Surgery. 2010;142(4):472-86. doi: 10.1016/j.otohns.2010.02.010..

42. Rostami K, Khadjooi K, Abasaeed-Elhag R, Ishaq S. How to evaluate a manuscript for publication? Gastroenterology and hepatology from bed to bench. 2011;4(2):58-62.

43. Rutkowski JL, Cairone JV. How to review scientific manuscripts and clinical case reports for Journal of Oral Implantology. The Journal of oral implantology. 2009;35(6):310-4. doi: 10.1563/aaid-joi-d-09-00053r1.1.

44. Salasche SJ. How to "peer review" a medical journal manuscript. Dermatologic surgery : official publication for American Society for Dermatologic Surgery [et al]. 1997;23(6):423-8. doi: 10.1111/j.1524-4725.1997.tb00081.x.

45. Schüttpelz-Brauns K, Stosch C, Matthes J, Himmelbauer M, Herrler A, Bachmann C, et al. Recommendations for reviewing a manuscript for the GMS Zeitschrift für Medizinische Ausbildung. GMS Zeitschrift fur medizinische Ausbildung. 2010;27(5):Doc75. doi: 10.3205/zma000712.

46. Seals DR, Tanaka H. Manuscript peer review: a helpful checklist for students and novice referees. Advances in physiology education. 2000;23(1):52-8. doi: 10.1152/advances.2000.23.1.S52.

47. Simpson KJ. Reviewing an Original Research Manuscript for the International Journal of Exercise Science: A Guide for Students and Professionals. International journal of exercise science. 2008;1(2):43-9.

48. Smith DV, Stokes LB, Marx K, Aitken SL. Navigating manuscript assessment: The new practitioner's guide to primary literature peer review. Journal of oncology pharmacy practice : official publication of the International Society of Oncology Pharmacy Practitioners. 2019;25(1):94-100. doi: 10.1177/1078155218761222.

49. Smolčić V, Simundić AM. Peer-review policy and guidelines for Biochemia Medica Journal. Biochemia medica. 2014;24(3):321-8. doi: 10.11613/bm.2014.034.

50. Stahel PF, Moore EE. How to review a surgical paper: a guide for junior referees. BMC Med. 2016;14:29. doi: 10.1186/s12916-016-0578-6.

51. Stone WJ, Navalta JW, Lyons TS, Schafer MA. From the Editors: A Guide for Peer Review in the Field of Exercise Science. International journal of exercise science. 2018;11(1):1112-9.

52. Sucato GS, Holland-Hall C. Reviewing Manuscripts: A Systematic Approach. Journal of pediatric and adolescent gynecology. 2018;31(5):441-5. doi: 10.1016/j.jpag.2018.06.005.

53. Sylvia LM, Herbel JL. Manuscript peer review--a guide for health care professionals. Pharmacotherapy. 2001;21(4):395-404. doi: 10.1592/phco.21.5.395.34493.

54. Talanow R. JRCR - updated author and reviewer guidelines for 2014. J Radiol Case Rep. 2014;8(6):39-40. doi: 10.3941/jrcr.v8i6.2292.

55. Tandon R. How to review a scientific paper. Asian journal of psychiatry. 2014;11:124-7. Epub 2014/09/25. doi: 10.1016/j.ajp.2014.08.007.

56. Venne V. Reviewing manuscripts for the Journal of Genetic Counseling: practical suggestions. Journal of genetic counseling. 2015;24(2):189-92. doi: 10.1007/s10897-014-9802-8.

57. Walker BF. How to critique an article on therapy. Australasian chiropractic & osteopathy : journal of the Chiropractic & Osteopathic College of Australasia. 1997;6(2):52-7.

58. Wilson JR. Responsible authorship and peer review. Science and engineering ethics. 2002;8(2):155-74. doi: 10.1007/s11948-002-0016-3.

59. Vintzileos AM, Ananth CV, Odibo AO, Chauhan SP, Smulian JC, Oyelese Y. The relationship between a reviewer’s recommendation and editorial decision of manuscripts submitted for publication in obstetrics. American Journal of Obstetrics and Gynecology. 2014;211(6):703.e1-.e5. doi: 10.1016/j.ajog.2014.06.053.

60. Glonti K, Cauchi D, Cobo E, Boutron I, Moher D, Hren D. A scoping review on the roles and tasks of peer reviewers in the manuscript review process in biomedical journals. BMC Med.

2019;17(1):118. doi: 10.1186/s12916-019-1347-0.

61. Coronel R, Opthof T. The role of the reviewer in editorial decision-making. Cardiovascular Research. 1999;43(2):261-4. doi: 10.1016/S0008-6363(99)00177-7.

62. Lee CJ, Sugimoto CR, Zhang G, Cronin B. Bias in peer review. Journal of the American Society for Information Science and Technology. 2013;64(1):2-17. doi: 10.1002/asi.22784.

63. Saxe JP. Opening the Black Box of Peer Review. Cell stem cell. 2019;24(6):843-8. doi: 10.1016/j.stem.2019.05.017.

64. Ross-Hellauer T, Deppe A, Schmidt B. Survey on open peer review: Attitudes and experience amongst editors, authors and reviewers. PloS one. 2017;12(12):e0189311. doi: 10.1371/journal.pone.0189311.

65. Shattell MM, Chinn P, Thomas SP, Cowling WR, 3rd. Authors' and editors' perspectives on peer review quality in three scholarly nursing journals. Journal of nursing scholarship : an official publication of Sigma Theta Tau International Honor Society of Nursing. 2010;42(1):58-65. doi: 10.1111/j.1547-5069.2009.01331.x.

Supplementary information

S1 File. PRISMA extension for scoping reviews checklist.

S2 File. Search strategy.

S3 File. Included review checklist items.

Fig.1

Click here to access/download;Figure;FIg.1.docx

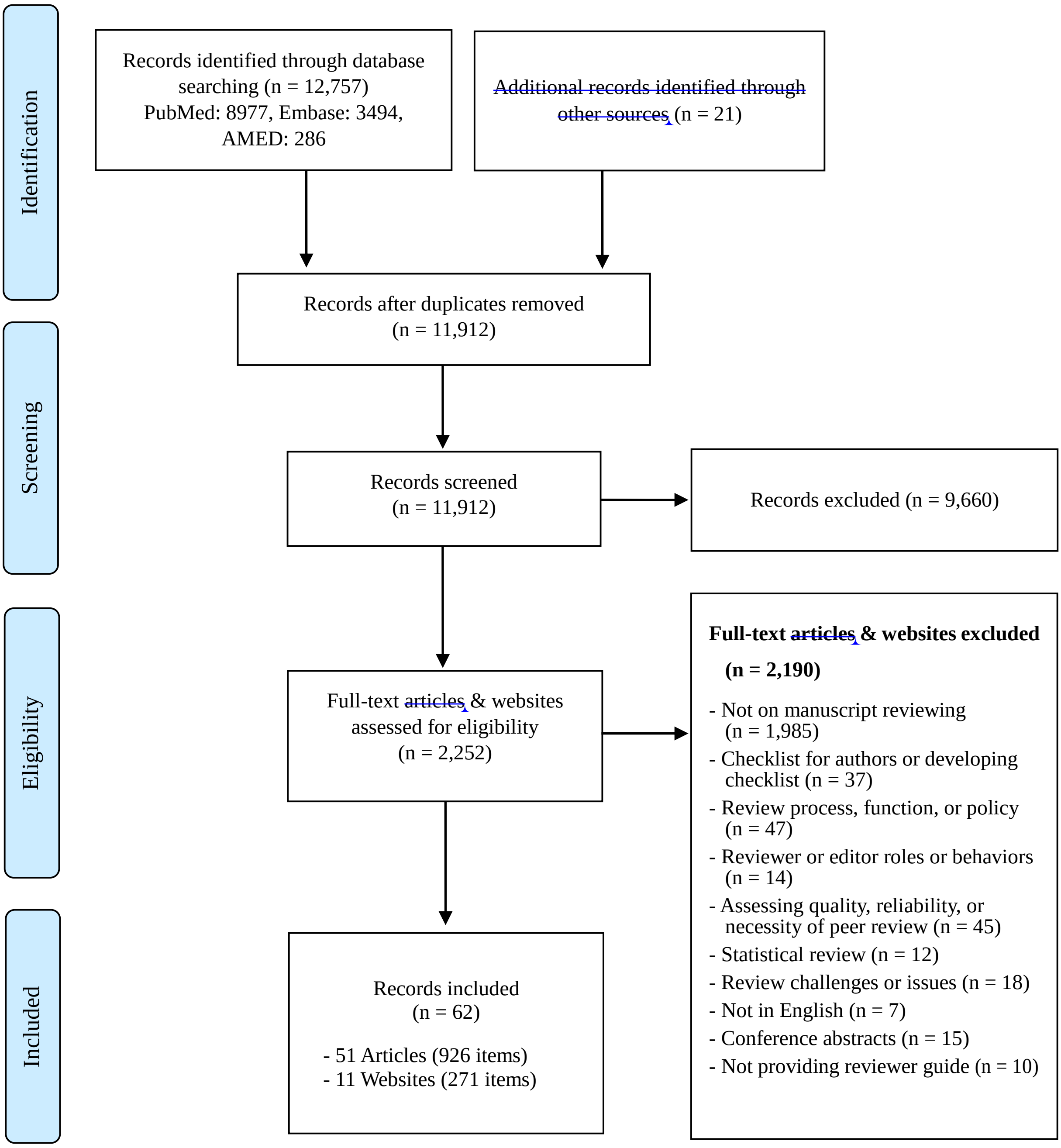

Fig.2

Click here to access/download;Figure;FIg.2.docx

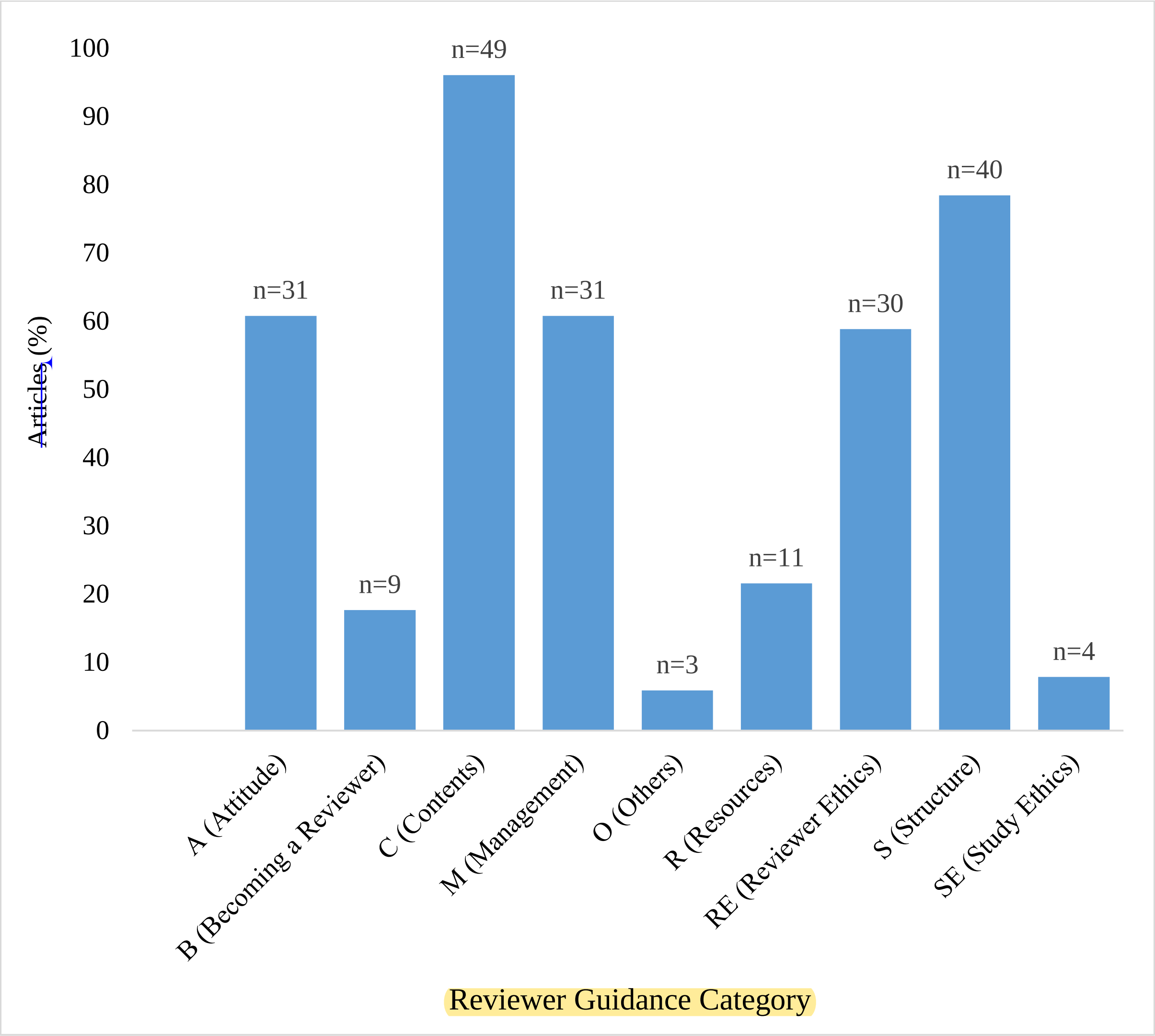

Fig.3

Click here to access/download;Figure;Fig.3.tif

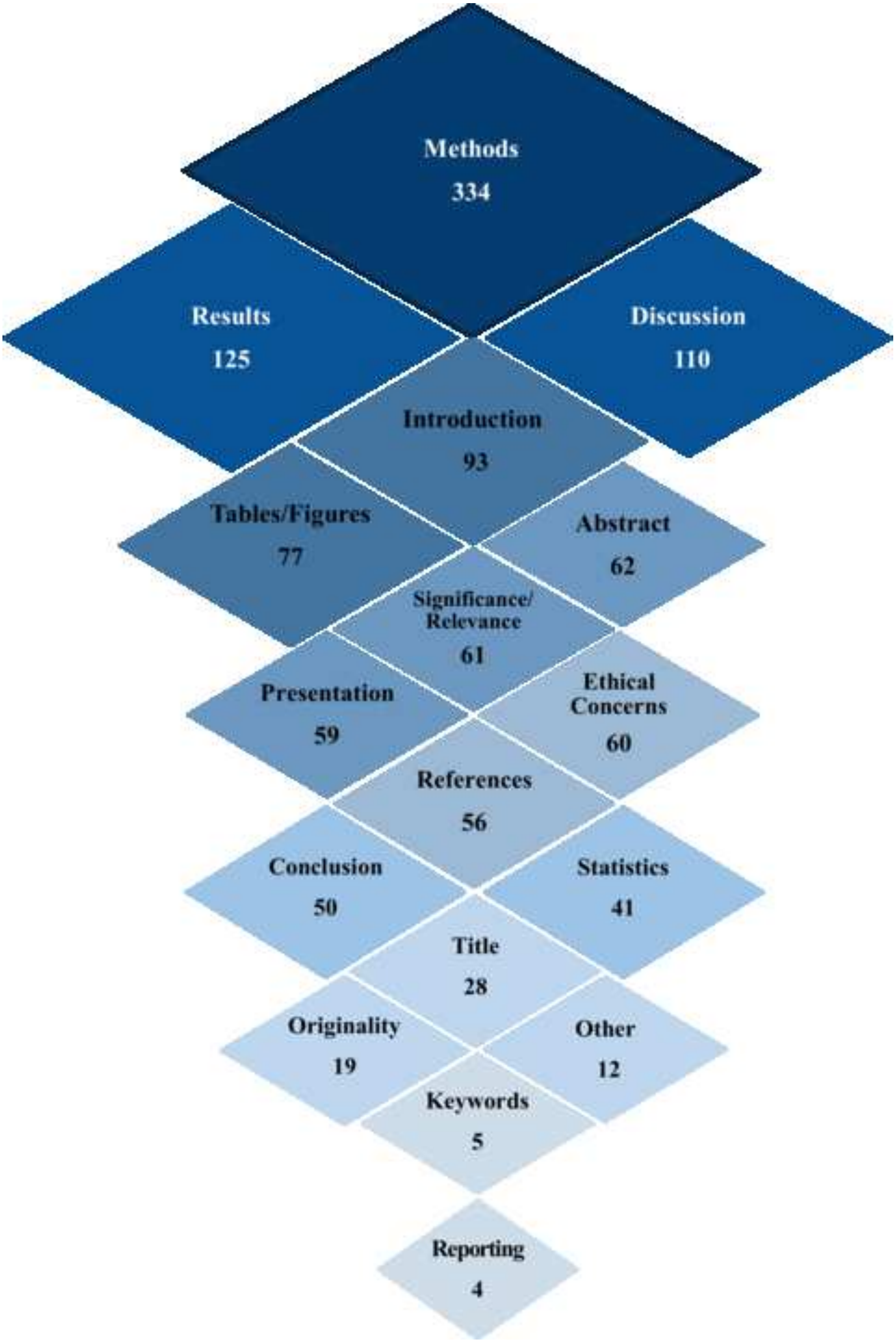

Click here to access/download
Supporting Information
S2_File_Search strategy.docx

S2 Search strategy

Click here to access/download
Supporting Information
S1_File-PRISMA-ScR.docx

S3 Included items

Click here to access/download
Supporting Information
S3_File_Included review checklist items.docx
