# Supplementary material for: A scoping review on biomedical journal peer review guides for reviewers
Source: PLoS One. 2021 May 20;16(5):e0251440. doi: 10.1371/journal.pone.0251440 (PMC8136639; doi:10.1371/journal.pone.0251440)
Supplement: Attachment — Submitted filename: PLOS one Song and colleagues 2021 .pdf [file pone.0251440.s007.pdf]

Song and colleagues address a critical issue in medical scholarship ecosystem, namely, the role of peer review. Their scoping review addresses a practical point about the availability of peer review guidance/checklist for manuscripts. The scoping review identified a large number of reports that met the scoping reviewer's inclusion criteria. The authors provide some information about the included documents. While I think the scoping review provides some utility for readers, I think it needs more rigor and interpretation before it can be more meaningful to readers.

I am delighted that the authors registered their scoping review and used PRISMA-ScR to aim in reporting their scoping review.

A main concern of mine is with the output of the results. While the authors provide a long list of peer review guidance (Table 1), as a reader I'm lost. What usefulness to readers is an editorial with guidance criteria A,C,M,R,S (e.g., Alam, 2015)? If I go to Figure 2, I can try and unpack what this means. For example, I can see that A is 'attitude'. But from a reader's point of view what does this mean for peer reviewing? One way of solving this issue is to include a box with a clear glossary of explanation for each criterion in Figure 2 – this information should be included in the Methods section. The authors also need to describe how these criteria were selected and developed – please include this in the methods section as well.

The other aspect of Table 1 that concerns me is that it appears each row is equivalent to each other. There is no evidence-based hierarchy. For example, is an editorial with no checklist items as informative/evidence based as a guideline (e.g., Marusic, 2005). Should there no be a vetting and organization of all the guidance documents included? For this reason, I do wonder whether there is a need for the authors to complete some form of validity assessment of all of the included documents. You as authors, and readers more generally, need some way of separating the wheat from the chaff. The results of the scoping review are also silent on whether there is overlap between the guidance documents. This point relates to the discussion. I have included a recent systematic review, also about checklists. The authors might find it helpful, particularly in how they re-imagine their discussion.

I found the discussion unhelpful. A more useful discussion would be a more in-depth interpretation of the included checklists/guidance. For example, do items overlap, are some checklists/guidance more helpful than others; should a new one be developed; is there merit in getting a the various groups involved and developed a more standardized checklist/guidance? The discussion is silent on whether the better guidance should be evidence based?

Was the search strategy peer reviewed (PRESS; McGowan J, Sampson M, Salzwedel DM, Cogo E, Foerster V, Lefebvre C. PRESS Peer Review of Electronic Search Strategies: 2015 Guideline Statement. J Clin Epidemiol. 2016 Jul;75:40-6). I also wonder whether the authors should have searched other sources, such as YouTube (we did this in the attached systematic review)? For example, is there merit on including Publons Academy? There may be merit (face validity) is examining potential resources that might exist by the International Committee of Medical Journal Editors; the World Association of Medical Editors; I'm not convinced excluding

searching the EQUATOR library of reporting guidelines is reasonable. To be honest I do not know how many reporting guidelines make explicit mention of peer reviewers. I know that CONSORT explicitly mentions this “We developed CONSORT 2010 to assist authors in writing reports of randomized controlled trials, editors and peer reviewers in reviewing manuscripts for publication, and readers in critically appraising published articles.” as does the forthcoming PRISMA update “In order to achieve this, we encourage authors, editors and peer-reviewers to adopt the guideline”. It is possible to acknowledge this in the limitations of your scoping review.
