# Supplementary material for: A scoping review on biomedical journal peer review guides for reviewers
Source: PLoS One. 2021 May 20;16(5):e0251440. doi: 10.1371/journal.pone.0251440 (PMC8136639; doi:10.1371/journal.pone.0251440)
Supplement: Attachment — Submitted filename: Response to reviewers.docx [file pone.0251440.s009.docx]

Response to reviewers

Once again, we are grateful to the reviewers for their additional comments and suggestions to better improve our manuscript. Our point by point responses are as follows:

Reviewer #1:

1. One thing I noticed that should be corrected prior to publication, is the renaming of the "article type" column in Table 1. I think it should be relabeled to be "publication type."

Thank you for pointing the missed-out correction. We have now corrected it to “publication type.”

Reviewer #2:

1. I did forget to ask the authors as to whether they searched the website of the International Congress on Peer Review and Scientific Publication (https://peerreviewcongress.org/ ). Even as a measure of face validity, it would be reasonable to search. I assume the search strategy used by the authors picked up the journal Research Integrity and Peer review (https://researchintegrityjournal.biomedcentral.com/ )?

Thank you for your comments. We have searched peerreviewcongress.org.

For *Research Integrity and Peer Review* (journal), the journal is on PubMed so they were searched together with others when we did the search.

2. In the Methods section – 2.4 Outcomes, the authors state “The criteria were developed together by the authors and agreed by all authors”. I appreciate the criteria were developed together by the authors. I think this requires some unpacking. Was this based on anecdotal experiences of the authors as peer reviewers; evidence-based publications to inform the criteria or some other way? My point here is that a reader interested in replicating your methods needs details.

Thank you for bringing this to our attention. We have revised the sentence to provide more details for readers:

“Before going through the screening, after the search of literature, the authors randomly pre-screened 10 publications to look for possible review guidance criteria, and based on the discussion by all authors with experience as peer reviewers, the criteria were developed.”

3. Please consider adding this randomized trial (protocol) in the discussion (Speich B, Schroter S, Briel M, Moher D, Puebla I, Clark A, Maia Schlüssel M, Ravaud P, Boutron I, Hopewell S. Impact of a short version of the CONSORT checklist for peer reviewers to improve the reporting of randomised controlled trials published in biomedical journals: study protocol for a randomised controlled trial. BMJ Open. 2020 Mar 19;10(3):e035114. doi: 10.1136/bmjopen-2019-035114. PMID: 32198306; PMCID: PMC7103787). It is an example of a standardized checklist for peer reviewing randomized trials.

Thank you for suggesting an additional relevant paper which would enrich our discussion further. It is an important study and add to the evidence. We have added the protocol of the randomized controlled trial. We have added the below text in our discussion:

“This kind of attempt is still in progress as seen in a study protocol of randomized controlled trial by Speich et al.[87]. This trial aims to evaluate the effectiveness or the need of providing a short version of the CONSORT checklist and its explanation to peer reviewers for them to check during peer review.”
